# The Crystal Chemistry and Topology of Modular Structures. III. 2D and 3D Zeolites Containing Tetrahedral Layers with the Apophyllite-Type Topology

**DOI:** 10.3390/molecules30224477

**Published:** 2025-11-20

**Authors:** Sergey M. Aksenov, Nikita V. Chukanov, Ramiza K. Rastsvetaeva, Dmitry Yu. Pushcharovsky, Dina V. Deyneko, Galina O. Kalashnikova, Ivan G. Tananaev, Peter C. Burns

**Affiliations:** 1 Laboratory of Arctic Mineralogy and Material Sciences, FRC Kola Science Centre RAS, Apatity 184209, Murmansk Region, Russia; 2 Geological Institute, FRC Kola Science Centre RAS, 14 Fersman Str., Apatity 184209, Murmansk Region, Russia; 3 FRC Problems of Chemical Physics and Medicinal Chemistry RAS, Chernogolovka 142432, Moscow Region, Russia; nikchukanov@yandex.ru; 4 Kurchatov Crystallography and Photonics Complex, NRC Kurchatov Institute, Moscow 119333, Russia; rast.crys@gmail.com; 5 Department of Geology, Moscow State University, Moscow 199991, Russia; dmitp01@mail.ru; 6 Department of Chemistry, Moscow State University, Moscow 199991, Russia; deynekomsu@gmail.com; 7 Centre of Nanotechnology, FRC Kola Science Centre RAS, Apatity 184209, Murmansk Region, Russia; g.kalashnikova@ksc.ru; 8 I.V. Tananaev Institute of Chemistry and Technology of Rare Elements and Mineral Raw Materials, FRC Kola Science Centre RAS, Apatity 184209, Murmansk Region, Russia; geokhi@mail.ru; 9 Department of Civil and Environmental Engineering and Earth Science, University of Notre Dame, Notre Dame, IN 46556, USA; pburns@nd.edu

**Keywords:** 2D zeolite, apophyllite, tetrahedral net, rhodesite, günterblassite, topology, modularity

## Abstract

Materials of the 2D zeolite class retain local catalytically active sites and the stability of traditional zeolites but with layered structures. Synthetic and naturally occurring single- and multilayer apophyllite-related compounds are prototypes of advanced industrial materials for use in various technologies. Their surface chemistry allows for functionalization, and these layers serve as fundamental building blocks for zeolitic frameworks. The discovery of the first triple-layer silicate, günterblassite, provided a critical link that established a fundamental crystal–chemical relationship between layered and framework structures in a wide range of micro- and mesoporous minerals and synthetic materials. The most prominent topic in the development of 2D zeolites remains the synthesis and structural characterization of these 2D zeolite structures This review offers a comprehensive overview of the current state of 2D and 3D zeolites constructed based on apophyllite-type layers. In accordance with the terms of modular crystal chemistry, we present a straightforward classification scheme based on the topological and symmetrical distinctions of the layers and provide ways for their stacking, creating a valuable basis for understanding the modular assembly of advanced porous materials.

## 1. Introduction

Inorganic zeolites are characterized by 3D microporous structures [1,2] and play key roles in industrial catalysis, adsorption, sensing, ion exchange processes, etc. [3,4,5,6,7,8,9]. Their widespread use is due to the powerful combination of acidity, high hydrothermal stability, and molecular sieving capabilities. However, the small effective sizes of their pores and channels impose significant limitations on mass transfer, leading to diffusion barriers that reduce catalytic efficiency, accelerate coking, and shorten catalyst lifetimes [10,11]. These problems were partially solved by the synthesis of two-dimensional (2D) zeolites [11,12,13,14,15,16,17]. Two-dimensional zeolites are porous materials prepared from lamellar precursors, in which the crystalline nano layers are joined weakly in one specific direction. Due to the absence of a covalent bond, the stacking sequence can be manipulated and the structural diversity can be controlled [13,18]. The synthesis of these silica-based materials has promoted considerable interest, and since the original disclosure, there has been impressive progress in the development of many new mesoporous solids based on a similar mechanism of templating [19,20].

Materials of the 2D zeolite class retain local catalytically active sites and the stability of traditional zeolites but with layered structures. Moreover, layered silicates can be used as precursors for new types of framework zeolites by converting Si–OH groups into Si–O–Si bonds via a topotactic reaction, wherein the topology of the silicate layer remains unchanged and the bonds within the layer are not broken or rearranged during the condensation process [21,22].

The first layered zeolite precursor, MCM-22P, was prepared by Mobil researchers in 1990 [23,24]. MCM-22P was later used for the synthesis of the MCM-22 molecular sieve [25], which consists of stacked layers that contain two-dimensional 10-membered ring channels. The RUB-24 and RUB-41 zeotypes were synthesized by structural conversion of RUB-18 and RUB-39 layered silicates, respectively [26,27]. The layered (alumino) silicate precursor PREFER [28] has been used for synthesis of the **FER**-type zeolite. Moreover, a similar approach has been recently applied for the synthesis of the silica zeolite ZEO-3, which is characterized by a multidimensional, interconnected system of extra-large pores. It was synthesized by the unprecedented one-dimensional to three-dimensional (1D-to-3D) topotactic condensation of a chain silicate [29].

Currently, study of layered zeolites is one of the most active and promising areas of development in the realm of porous and hierarchical materials. Investigations in this area have extended the fundamental concept of framework structures and provided a new platform for the synthesis of highly active materials with large tunable pore structures and architectures. Many unexpected types of materials and phenomena have been revealed, including new synthetic methods using tailored bifunctional structure-directing agents (SDAs) [30,31] and unprecedented top–down synthesis methods represented by the ADOR (“assembly–disassembly–organization–reassembly”) strategy [32,33]. A classification system and extended concept of zeolite structures integrating various 2D and traditional 3D shapes has also been proposed [34].

The topology of a zeolite is defined by its primary structure. Different types of zeolite monolayer assemblies are secondary structures. Based on the literature data, 15 different single-layered forms have been identified [34] and the layered forms used for different topologies have been described. It serves as a classification tool for recognizing and validating known materials. Recent results in the synthesis, characterization, and application of 2D zeolites of **MWW** and **MFI** types, which are the most versatile and correspond to almost all known forms of 2D zeolites, highlighted some basic open problems corresponding, in particular, to the absence of the layer precursor for **MFI**-type zeolite [16].

The most prominent topic in the development of 2D zeolites remains the synthesis and structural characterization of these 2D zeolite structures [35]. Apophyllite-related materials are of great industrial and technological interest because they are analogs of naturally occurring single-layer silicates, and their reactions with chlorosilanes yield apophyllite-based organosilicate polymers on both sides of the silicate layer, which possess a unique combination of hydrophobic and hydrophilic properties [36,37,38]. In particular, hydrosilylation reactions with this Si–H group and Karstedt’s catalyst were performed to append functional groups of differing size and polarity, including the first reported synthesis of a glycosylated silicon sheet polymer derived from a naturally occurring silicate [38]. A similar mesoporous silica was produced by folding the single-layered polysilicate kanemite (NaHSi_2_O_5_ 3H_2_O) sheet structures, which are called FSMs (folded sheets mesoporous materials) [19,39]. The discovery of the first triple-layer silicate günterblassite [40,41] led to the unearthing of the fundamental crystal–chemical relationship between microporous silicate minerals and synthetic materials, containing silicate layers of apophyllite-type topology [42].

This review continues our studies of microporous modular materials [43,44,45,46,47,48,49] and aims to provide a comprehensive overview of the current state of 2D/3D zeolites based on apophyllite-type layers. Possible classification schemes are given, based on the topologically and symmetrically distinct apophyllite-type layers.

## 2. Polymorphism of Tetrahedral Layers

Silicate tetrahedral anions exhibit wide polymorphism induced by the different types of the [SiO_4_]^4−^ tetrahedra linkages [50,51,52,53,54,55]. The polycondensation of silicon–oxygen tetrahedra with the formation of high-dimension chain-ring structures can be described as follows [56]:

*i*(SiO_4_)^4−^ = (Si*_i_*O_3*i*+1–*j*_)^2(*i*+1−*j*)−^ + (*I* – 1 + *j*)O^2−^, (1)

where *i* is the polyanion size (the number of silicon atoms) and *j* is the number of intramolecular closures of Si–O–Si silicon–oxygen bonds. These reactions lead to the formation of chain (*j* = 0) and various cyclic (or ring, *j* ≥ 1) structures. The concentration (activity) of oxygen ions is considered a characteristic of the basicity of the silicate melt [57] and plays an important role in the evaluation of the degree of polymerization of the silicon–oxygen matrix and the size distribution of anionic complexes [56].

The general basis of structural polymorphism of silicate polyanions has been shown in detail [51,52,58], and, in particular, for silicates with the ratio Si:O = 2.5, the different possible examples of tetrahedral anions are known [58]. In accordance with the stoichiometry of tetrahedral anions [49,59], all unbranched tetrahedral single layers [55], unbranched double chains and tubular chains [54], as well as double rings [51], are formed only by one sort of [Si O[2]3 O[1]]-tetrahedron (with three [2]-connected “bridging” and one [1]-connected “apical” oxygens). As the result, they are characterized by the same general formula [Si_2*n*_O_5*n*_].

The concept of polymorphism in silicon–oxygen tetrahedral polyanions with identical composition (*T*:O ratios) was first formulated by N.V. Belov [50]. The development of these concepts in the early and mid-1980s led to the creation of a comprehensive and coherent classification of silicates and their structural analogs—phosphates, germanates, and others—revealing over 100 tetrahedral complexes when considering their most striking features (the number of tetrahedra in rings, the periodicity of chains in bands, the shape of rings in layers, etc.) [50,51,52,58,60,61,62]. Within this framework, the structural diversity of silicate polyanions with the general formula *T*_2_O_5_, characteristic of many types of silicate layers, became particularly evident.

Layered silicates are among the most common minerals in nature, constituting over 10% of the Earth’s crust. They are typical of sedimentary strata (kaolinite, illite, smectites, etc.), metamorphic rocks (muscovite, biotite, chlorites, talc), granites (biotite), granitic pegmatites (muscovite, biotite, lepidolite), as well as basic and alkaline rocks (chlorites, serpentines, biotite, vermiculite, etc.) [63,64,65,66].

Minerals of the layered silicate class exhibit significant structural diversity and demonstrate many crystal–chemical phenomena that complicate actual structures. This is primarily because tetrahedra can combine into layers, forming nets containing various types of rings (loops): four-membered, five-membered, six-membered, eight-membered, and twelve-membered rings, and others [50,51,52,54,55,60]. Such diversity of possible topologies is due to the origin. In particular, the structures of the most common minerals, including rock-forming minerals (e.g., micas, smectites, chlorites, serpentines and related compounds [66]) are characterized by the layers containing only six-membered rings. Nets of tetrahedra containing other types of rings can only be constructed by combining rings of same or different sizes. Meanwhile, minerals containing networks with different ring types are relatively rare but exhibit considerable structural diversity.

Variations in silicon–oxygen motifs can be conveniently visualized using graph theory [67,68,69,70], where each tetrahedron is represented as a graph node, and the presence of an edge between two nodes indicates that these tetrahedra share oxygen atoms. The symbol of such a graph can be written as a sequence $p_{1}^{r_{1}}p_{2}^{r_{2}}...p_{n}^{r_{n}}$, where *p*_1_, *p*_2_, … *p_n_* represent the number of nodes in a ring, and *r*_1_, *r*_2_, … *r_n_* denote the number of rings of the corresponding type in the graph. Thus, the symbol for the network in members of the mica, chlorite, and kaolinite–serpentine groups, talc, etc. [64], whose tetrahedral layers consist solely of six-membered rings, can be written as 6^1^, while the [Si_5_O_14_] layer in the structure of molybdophyllite, Pb_8_Mg_9_(Si_10_O_28_)(CO_3_)_5_(OH)_8_O_2_·H_2_O, britvinite, Pb_15_Mg_9_(Si_10_O_28_)(BO_3_)_4_(CO_3_)_2_(OH)_12_O_2_, and related minerals [71,72] composed exclusively of twelve-membered rings, corresponds to the notation 12^1^. The network symbol for the cavansite structure [73,74], which contains two types of rings (e.g., four- and eight-membered rings in a 1:1 ratio), is represented as 4^1^8^1^, whereas the net with three types of rings (four-, six-, and eight-membered) in a 1:1:1 ratio, as in the structures of sazhinite-(La), Na_3_La[Si_6_O_15_]·2H_2_O, or sazhinite-(Ce), is denoted as 4^1^6^1^8^1^ [75]. Topologically related unbranched single layers of sazhinite have a close relationship with those in armstrongite CaZr[Si_6_O_15_]·3H_2_O [76], dalyite K_2_Zr[Si_6_O_15_] [77], as well as synthetic compounds NaNd[Si_6_O_13_(OH)_2_] [78], α-K_3_Nd[Si_6_O_15_] [79], K_3_Eu[Si_6_O_15_]·2H_2_O [80,81], and Rb_1.66_Cs_1.34_Tb[Si_5.43_Ge_0.57_O_15_]·H_2_O, which can be described as an alteration of the xonotlite-like bands formed by eight-membered rings, with the bands formed by six- and four-membered rings. All these structures comprise silicate sheets which can be considered as the result of the condensation of wollastonite-like chains [82]. Representatives of the family of hydrous alkali silicates with the general formula *A*_3_*REE*[Si_6_O_15_]·2H_2_O (where *A* = Na, K, H_3_O; *REE* = La, Eu) contain silicon–oxygen layers of the type of 4^1^5^1^6^1^8^2^ with four types of rings [83].

A two-dimensional graph, as described in the previous paragraph, does not account for the orientation of tetrahedra relative to the plane of the net. Tetrahedral layers characterized by identical net topology but differing in the orientation of their tetrahedra are referred to as “geometric stereoisomers” [84,85,86]. These differences in tetrahedral orientation further increase the diversity of layered tetrahedral silicon–oxygen motifs. Additionally, SiO_4_ tetrahedra can be substituted by chemically different *T*O_4_ tetrahedra (*T* = Be^2+^, B^3+^, Al^3+^, etc.) or even *M*O*_n_* polyhedra [where *M* represents cations with high force characteristics, such as Ti^4+^, Zr^4+^, Nb^5+^, etc., with *n* equal to five (semi-octahedron = tetragonal pyramid) or six (octahedron)], thereby expanding the number of chemical and heteropolyhedral analogs [49,59,87,88].

### 2.1. Stoichiometry of Single-Layer Tetrahedral Anions

Any tetrahedral layer can be viewed as the condensation of tetrahedral chains. The stoichiometry of an isolated unbranched single tetrahedral layer (uB—“unbranched”), formed by a single cation type, can be expressed as follows [51]:

(2) uB,1∞2TPnpØ3np−∑1nlinpVT+3VØ−VØ∑1nli,

where **P** is the periodicity of the single chain forming the layer; *n* is the chain multiplicity (i.e., the number of chains linked together in the layer); *p* = **P**; *l* is the number of linkages between adjacent chains in the repeat period of the anions (*l* ≤ *nP*); *V_T_* is the charge of the *T*-cation; and $V_{Ø}$ is the charge of the Ø-anion.

### 2.2. Formula Generation Function

The *T*:Ø ratio in the layer formula (where *T* is the tetrahedrally coordinated atom and Ø is the anionic ligand) depends on the number of graph nodes, their local environment (each node can be connected to two, three, or four neighboring nodes), and the ratio of physically distinct nodes. However, the parameters **P**, *n*, and *l* are not the only ones that can be used to distinguish between topologically different layers [51].

The stoichiometry of silicon–oxygen layers can be determined using the formula generation function proposed by F. Hawthorne [53,88]:

*F*_(*i*,*j*,…)_ = *f*(*i*,*j*,…), (3)

where *i*, *j*,… are special indices corresponding to different topological features of the structure, in particular, the orientation of tetrahedra relative to the layer plane. In its general form, it is expressed as follows:

*F*_[*N*(*h*+*i*+*k*+*l*+*n*)+*j*+*m*]_ = *T*_[*N*(*h*+*i*+*k*+*l*+*n*)+*j*+*m*]_Ø_[3*Nh*+(*N*+2)*i*+(4−*N*)*j*+2.5*Nk*+(1.5N+1)*l*+(3−0.5*N*)*m*+2*Nn*]_, (4)

where *N* = 1 or 2 for single- and double-layer silicates, respectively.

A further development of this approach is the “structural hierarchy” of layered silicates based on the ratio of *T*-cations to the total number of Ø-ligands (*T*:Ø) in the layered anion formula, which relies on the topological features of tetrahedral networks [55].

## 3. Nets of the Apophyllite-Type: Topology and Structural Isomerism

**Figure 1:**
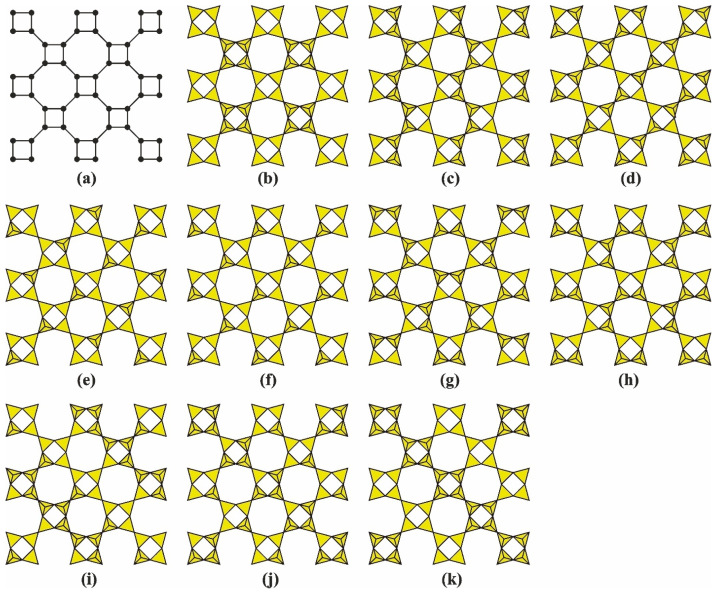
Schematic representation of a net with 4^1^8^1^ topology (**a**) and geometric stereoisomers of [Si_4_O_10_] layers in the structures of representatives of the apophyllite group [107] (**b**), cavansite [73] (**c**), seidite-(Ce) [108] (**d**), mountainite [101] (**e**), shlykovite [109] (**f**), carletonite [102,103] (**g**), **KNARSI** [110] (**h**), **VSH**-12Cs [104] (**i**), K_2_Sb(Si_4_O_10_)(OH) [105] (**j**), and KEu_2_(Si_4_O_10_)F [106] (**k**).

Of particular interest are minerals and synthetic compounds containing tetrahedral nets [*T*_4_Ø_10_]*^q^*^−^ with the topology of the apophyllite-type sheet (Figure 1a) [42,51,52], composed of four- and eight-membered rings of tetrahedra in a 1:1 ratio (thus, the symbol of such a net is [4^1^8^1^) [67]. Each node in such a net is surrounded by three neighboring nodes and participates in the formation of one four-membered ring and two eight-membered rings, so the Schläfli symbol [89] for the nodes of the apophyllite-type graph is (4.8^2^) [51,90].

### 3.1. Topology and Structural Isomerism of the Single-Layer Apophyllite-Type Net

In most compounds containing layered tetrahedral polyanions with apophyllite-type topology, Si is most common among the possible *T* atoms. However, such single layers can also be formed by different types of atoms (including those with different valence states), such as Si^4+^ and Be^2+^, B^3+^, or As^5+^ in gadolinite supergroup minerals [91,92] as well as P^5+^ and Be^2+^ [93,94,95]. Additionally, such layers can incorporate tetrahedra centered by P^5+^ and Zn^2+^/Cu^2+^ in kipushite, (Cu,Zn)_5_Zn(PO_4_)_2_(OH)_6_·H_2_O [96] and veszelyite, (Cu,Zn)_2_Zn(PO_4_)_2_·2H_2_O [97]; As^5+^ and Cu^2+^ in philipsburgite, (Cu,Zn)_6_(AsO_4_,PO_4_)_2_(OH)_6_·H_2_O [98]; and S^6+^ and Zn^2+^ in belousovite, KZn(SO_4_)Cl [99] and its synthetic analogs [100]. All these compounds are characterized by the isolated tetrahedral layers connected via additional cations without the formation of microporous structures.

Despite the extensive heterovalent isomorphism in tetrahedra, silicate, and aluminosilicate polyanions (with the predominance of Si^4+^ among *T* atoms), they represent the most chemically diverse family, and are characterized by a wide structural variability of layered motifs [51,52,55].

Nets of this type can differ in the orientation of tetrahedra, forming various structural isomers (Figure 1b–k) [42,69]. Each isomer has its own translation period and local layer symmetry. The orientation of tetrahedra can be conveniently described using a **ud**-matrix (**u**—“up”, **d**—“down”). Each row of this matrix describes the sequence of tetrahedra orientations in a single chain of tetrahedra from left to right, while the columns represent the alternation of chains from bottom to top [42]. The matrix dimensions (*m* × *n*), where *m* is the number of rows and *n* is the number of columns, depend on the translation period of the chains and their quantity. Structurally characterized representatives with apophyllite-type layers belonging to different stereoisomers are listed in Table 1.

For minerals, simple isomers described by a (2 × 4) matrix are typical. Among natural single-layer representatives, the most complex isomer is found in mountainite [101]; its silicon–oxygen layer is described by a (4 × 8) matrix. The same matrix (4 × 8) applies to the isomer in the structure of carletonite [102,103], although this layer is part of a more complex double-layer polyanion. The isomers in the structures of **VSH**-12Cs and **VSH**-12LiX [104], as well as K_2_Sb(Si_4_O_10_)(OH) [105] and KEu_2_(Si_4_O_10_)F [106], are also described by this matrix.

**Table 1:** Minerals and synthetic compounds with silicate layers displaying apophyllite-type topology.

Mineral/Compound	Symmetry of the Layer	The Ud-Matrix Description of the Layer	References
Apophyllite-related compounds	*P*4/*nmm*	$\left[\begin{array}{c} \begin{array}{cccc} u & u & d & d \end{array}\\ \begin{array}{cccc} u & u & d & d \end{array} \end{array}\right]$	[111]
Gillespite-group minerals			[112]
KSr_4_[Si_4_O_6_(OH)_4_]_2_(OH)_9_ (**AES**-18)			[113]
Cavansite	*Pmna*	$\left[\begin{array}{c} \begin{array}{cccc} u & d & d & u \end{array}\\ \begin{array}{cccc} u & d & d & u \end{array} \end{array}\right]$	[73]
Na_2_(VO)(Si_4_O_10_)(H_2_O)_4_ (**VSH**-13Na)			[104]
Seidite-(Ce) *	$P\bar{4}m2$	$\left[\begin{array}{c} \begin{array}{cccc} d & u & u & d \end{array}\\ \begin{array}{cccc} u & d & d & u \end{array} \end{array}\right]$	[108]
Shlykovite	*Pm*	$\left[\begin{array}{c} \begin{array}{cccc} d & d & u & d \end{array}\\ \begin{array}{cccc} u & d & d & d \end{array} \end{array}\right]$	[109]
Cryptophyllite			[109]
KNa_3_(UO_2_)_2_(Si_4_O_10_)(H_2_O)_4_ (**KNARSI**)	*P*2/*m*	$\left[\begin{array}{c} \begin{array}{cccc} d & u & u & u \end{array}\\ \begin{array}{cccc} d & u & d & d \end{array} \end{array}\right]$	[110]
Na_4_(UO_2_)_2_(Si_4_O_10_)(H_2_O)_4_ (**NARSI**)			[114]
KNa_3_(UO_2_)_2_(Si_4_O_10_)(H_2_O)_4_ (**USH**-1)			[115]
Mountainite	*Pma*2	$\left[\begin{array}{cccccccc} u & d & d & u & d & d & d & d\\ d & u & d & d & d & d & u & d\\ d & d & d & d & u & d & d & u\\ d & d & u & d & d & u & d & d \end{array}\right]$	[101]
Carletonite *	*P*4/*nbm*	$\left[\begin{array}{cccccccc} u & d & u & u & d & u & d & d\\ u & d & u & u & d & u & d & d\\ d & u & d & d & u & d & u & u\\ d & u & d & d & u & d & u & u \end{array}\right]$	[102]
Cs_2_(VO)(Si_4_O_10_)(H_2_O)*_x_* (**VSH**-12Cs)	*Pmc*2_1_	$\left[\begin{array}{cccccccc} d & d & d & u & u & u & u & d\\ u & d & d & d & d & u & u & u\\ u & u & u & d & d & d & d & u\\ d & u & u & u & u & d & d & d \end{array}\right]$	[104]
Li_2_(VO)(Si_4_O_10_)(H_2_O)*_x_* (**VSH**-12LiX)			[104]
K_2_Sb(Si_4_O_10_)(OH)	*P*2_1_/*m*	$\left[\begin{array}{cccccccc} d & u & d & u & d & d & u & u\\ u & u & d & u & d & u & d & d\\ d & d & u & u & d & u & d & u\\ d & u & d & d & u & u & d & u \end{array}\right]$	[105]
KEu_2_(Si_4_O_10_)F	*Pmma*	$\left[\begin{array}{cccccccc} u & u & d & d & d & d & u & u\\ u & u & u & u & d & d & d & d\\ d & d & u & u & u & u & d & d\\ d & d & d & d & u & u & u & u \end{array}\right]$	[106]

* In the crystal structures of seidite-(Ce) [108] and carletonite [102], the layer with the apophyllite-type topology is part of the double-layer silicate anion.

### 3.2. Structural Isomerism as a Factor in Combining Layers into Multilayer Polyanions

The orientation of tetrahedra relative to the layer plane facilitates the combination of these layers into double-, triple-, or multilayer polyanionic motifs. In general, regardless of the topology of the silicon–oxygen layer, the stoichiometry of double-layer polyanions can be obtained using Equation (4) for *N* = 2 [53]. Specifically, for double-layer apophyllite-type networks, it takes the form

*F*_2[*k*,*l*]_ = *T*_[2(*k*+*l*)]_Ø_[5*k*+4]_, (5)

where *k* is the number of nodes in the network with a **d-**configuration, and *l* is the number of nodes with a **u-**configuration (Table 1). For example, for a shlykovite-type layer, where *k* = 3 and *l* = 1, this would result in the following equation:

*F*_[3,1]_ = *T*_[2(3+1)]_Ø_[5×3+4]_ = [*T*_8_Ø_19_], (6)

which fully corresponds to the stoichiometry of the rhodesite double layer [116].

In multilayer polyanions (*N* > 2), the ratio of tetrahedral cations to anions depends on the specific mode of layer combinations. For a framework (*N* = ∞) (i.e., a framework where all oxygen atoms are bridging), the stoichiometry is as follows:

(7) { }∞3TnØ2nnVT+2VØ.

J.V. Smith thoroughly examined the isomerism of apophyllite-type layers (characterizing them using Schläfli symbols, as well as determining their symmetry and maximum possible symmetry) and identified 30 types that are capable of forming frameworks [90]. This revealed combination modes that produce microporous frameworks with wide channels.

## 4. Condensation of Apophyllite-Type Layers

As noted above, the varying orientation of tetrahedra relative to the tetrahedral layer plane, which determines the formation of structural isomers, creates prerequisites for their combination into multilayer tetrahedral polyanions (up to zeolite-type tetrahedral frameworks). Among compounds containing such multilayer polyanions, the most representative are those based on layers with apophyllite-type topology [42,51,55,90].

To date, structures have been identified that contain modules of condensed layers of apophyllite, shlykovite, mountainite, and seidite types. These layers either combine directly through layer-by-layer (topotactic) stacking or via the linking adjacent layers by additional tetrahedra. Furthermore, several compounds feature tetrahedral frameworks in which layers with apophyllite-type topology can also be distinguished.

### 4.1. Multilayer Polyanions Based on Shlykovite- and Mountainite-Type Layers

The most representative family consists of silicates containing tetrahedral layers [Si_4_O_9_(OH)]^3−^ and [Si_4_O_10_]^4−^, found in the crystal structures of shlykovite, KCa[Si_4_O_9_(OH)]·3H_2_O and cryptophyllite, K_2_Ca[Si_4_O_10_]·5H_2_O [109,117] (Figure 1e). The general composition of such a layer can be represented as [*T*_4_O^br^_6_Ø^ap^_4_]*^n^*^−^, where some part of silicon in the *T*Ø_4_ tetrahedra may be substituted with aluminum, and apical Ø^ap^ vertices may be protonated; O^br^ denotes bridging oxygen atoms between two *T*Ø_4_ tetrahedra. Such layers were first discovered as a part of double layers in the structures of minerals belonging to the rhodesite group and their synthetic analogs [51]. The discovery of shlykovite and cryptophyllite confirmed the general topological and stereoisomeric features of these layers, as well as the possibility of their condensation to form multilayer silicate polyanions.

**Figure 2:**
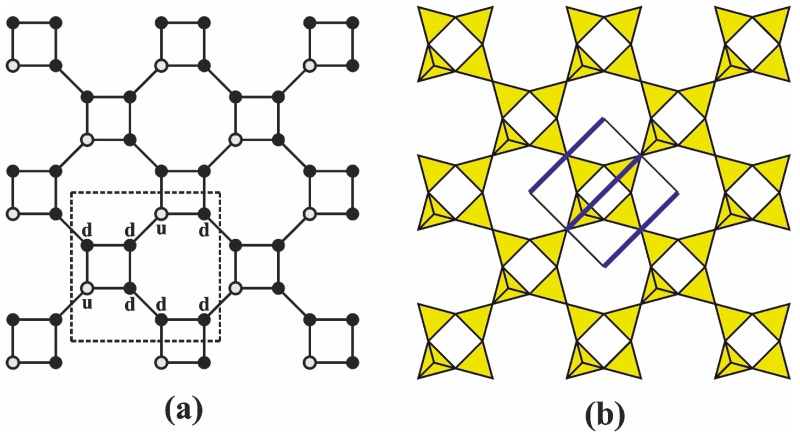
The shlykovite-type layer: graph-stereoisomerism (dashed line indicates nodes, which form the orientation matrix) (**a**) and corresponding tetrahedral layer (the mirror plane symmetry operations are shown in dark blue (**b**).

As mentioned earlier, polar shlykovite-type layers, corresponding to a 4^1^8^1^ net with apophyllite-type topology, are characterized by *Pm* symmetry (Figure 2, Table 1). The specific tetrahedral orientation within such a layer can be described by the following (2 × 4) matrix:

(8) $$\left[\begin{array}{c} \begin{array}{cccc} d & d & u & d \end{array}\\ \begin{array}{cccc} u & d & d & d \end{array} \end{array}\right].$$

**Figure 3:**
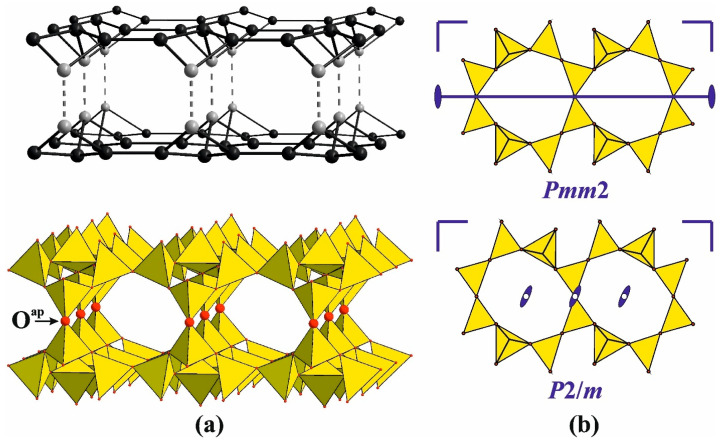
Combination of shlykovite-type layers forming a rhodesite-type module (**a**) and the possible symmetry of a two-layer rhodesite-type polyanion (**b**).

The formation of double-layer polyanions can be understood as the polycondensation of shlykovite-type layers through free O^ap^ vertices of tetrahedra inverted in opposite directions, leading to the formation of rhodesite-type double layers (Figure 3a). The stoichiometry of such double layers is [*T*_8_Ø_19_] = [*T*_8_O^br^_13_Ø^ap^_6_].

There are two distinct ways that shlykovite layers can be combined, and these result in two types of layers with different symmetry [118]. The layers can be linked by either of the following methods:

1. By reflection across a plane, preserving the overall polar motif of the shlykovite layer, with the double-layer symmetry becoming *Pmm*2.
2. Via a twofold rotation axis perpendicular to the existing mirror plane of the original layer, rendering the double layer nonpolar with *P*2/*m* symmetry (Figure 3b).

**Figure 4:**
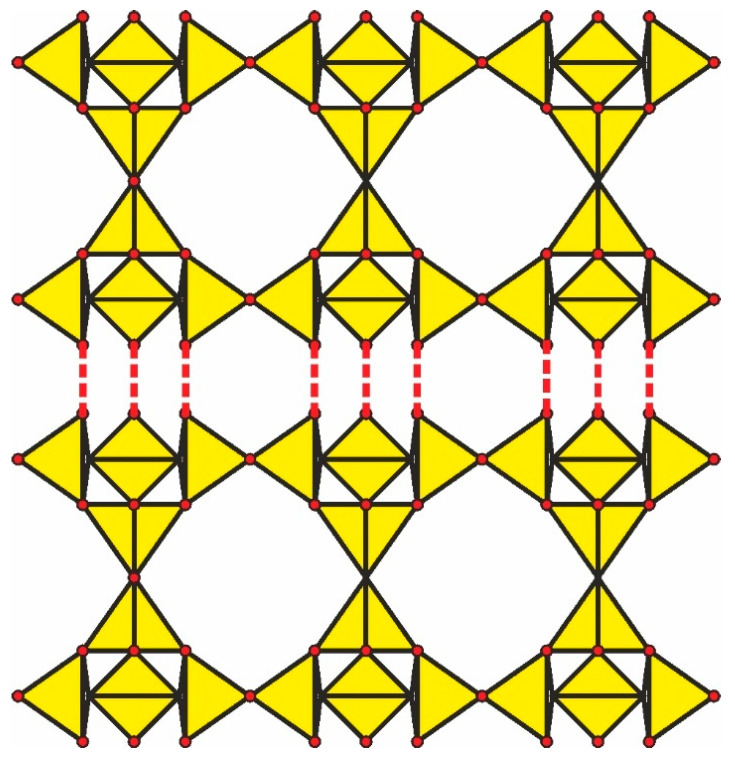
A possible path for the direct topotactic condensation (red dashed lines) of double-layer rhodesite-type silicate anions [119].

Further direct condensation of shlykovite layers is improbable (or extremely rare), so polycondensation mechanisms were not considered for a long time. Possible transformation pathways, using the synthetic K,Sr-analog of rhodesite (AES-19) [119] as a model, involved the extraction of alkaline earth elements from the prototype structure followed by topotactic condensation of rhodesite-type double layers (Figure 4). The IUPAC compendium of chemical terminology offered the following definition: “Topotactic transition: a transition in which the crystal lattice of the product phase shows one or more crystallographically equivalent, orientational relationships to the crystal lattice of the parent phase”. This means that the topology of the silicate layer (the [*T*O_4_]-tetrahedra connectivity pattern) stays the same and the bonds within the layer need not be broken or rearranged during the condensation process [21].

Based on the assumptions described above, a hypothetical framework structure model was proposed [119], but to date, it has not been realized in either natural or synthetic compounds.

### 4.2. Silicates with Triple-Layered Apophyllite-Type Complexes: The Günterblassite Group

**Figure 5:**
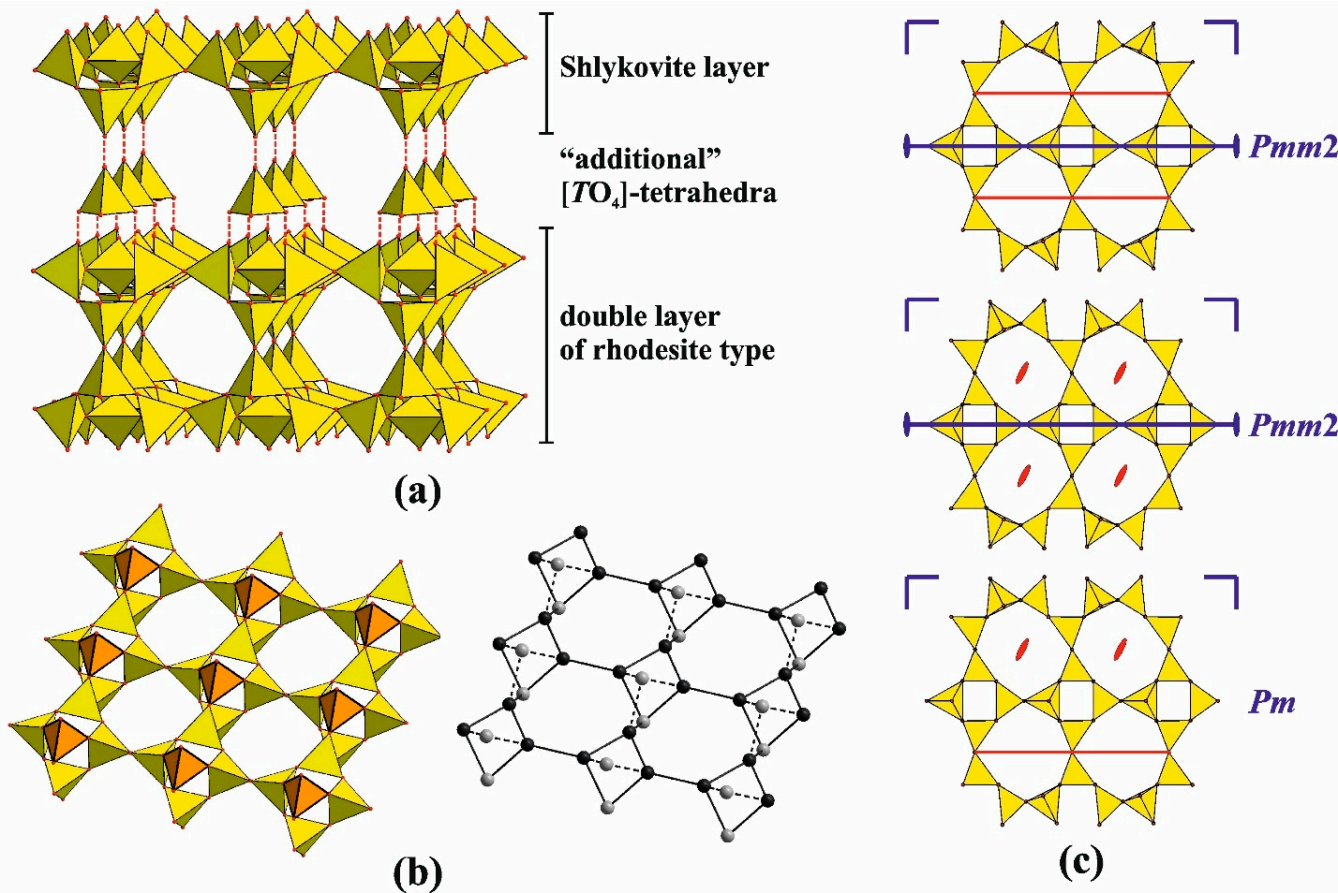
Linkage of the rhodesite-type double layer and shlykovite-type single layer via an “additional” [*T*O_4_] tetrahedron (**a**); the structure of the central layer (the “additional” tetrahedra are highlighted in orange) (**b**) and the possible symmetry of three-layer günterblassite-type polyanions (the local symmetry between adjacent shlykovite-type layers is shown in red) (**c**).

The crystal structure solution of günterblassite, (K,Ca,Ba,Na,□)_3_Fe[(Si,Al)_13_O_25_(OH,O)_4_]·7H_2_O, [40,41] revealed for the first time a triple-layered polyanion that retains the fundamental topology of the apophyllite-type. This structure forms through the insertion of an additional *T*O_4_ tetrahedron between single [*T*_4_O^br^_6_Ø^ap^_4_] layers of the shlykovite-/cryptophyllite-type and double [*T*_8_O^br^_13_Ø^ap^_6_] layers of the rhodesite-type, resulting in a triple [*T*_13_Ø_29_] = [*T*^add^*T*_12_O^br^_23_Ø^ap^_6_] layer of the günterblassite-type (Figure 5a).

The triple-layered silicate polyanion of the günterblassite-type comprises two outer shlykovite-type layers and a central layer (Figure 5b). The central layer, in isolation, forms a branched [*T*_5_Ø_11_] = [*T*^add^*T*_4_O^br^_9_Ø^ap^_2_] layer of the shlykovite-type, where an additional [*T*^add^O_4_] tetrahedron caps a triangular “platform” created by three apical Ø^ap^ vertices of tetrahedra in the same **d**-orientation (Figure 2a and Figure 5b). The polar character (symmetry *Pmm*2) is preserved because the additional tetrahedron shares the same orientation as the underlying tetrahedron. Stacking of outer layers can occur via multiple pathways (Figure 5c), all maintaining overall polarity.

Subsequent discoveries of umbrianite, K_7_Na_2_Ca_2_[Al_3_Si_10_O_29_]F_2_Cl_2_ [120], and hillesheimite, (K,Ca,□)_2_(Mg,Fe,Ca,□)_2_[(Si,Al)_13_O_23_(OH)_6_](OH)·8H_2_O [121], which contain similar triple-layered tetrahedral packages in distinct geological settings, validated the günterblassite model and demonstrated the natural prevalence of this layer condensation mechanism.

### 4.3. Transition from 2D to 3D Frameworks: Apophyllite-Type Net in Zeolite Architectures

The günterblassite condensation mechanism (insertion of additional tetrahedra) suggests a pathway toward silicates with extended tetrahedral blocks (with more than three shlykovite-type layers) and, ultimately, zeolite-like frameworks.

#### 4.3.1. Topological Characteristics of Framework Structures

**Figure 6:**
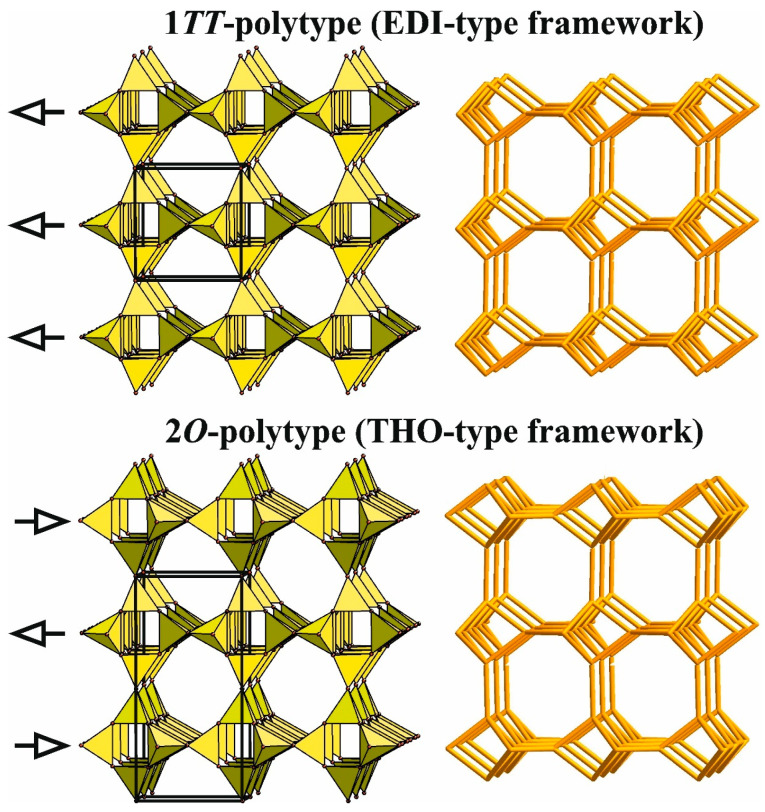
Idealized models of the 1*TT*- and 2*O*-polytypes (corresponding to **EDI**- and **THO**-frameworks) of crystal structures containing shlykovite modules and their topological differences. The arrows correspond to the arrangement of identically oriented layers.

**Figure 7:**
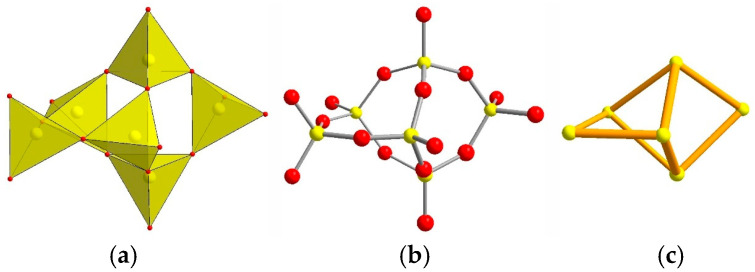
The main *compositional building unit* (CBUs) of a six-tetrahedron natrolite-like cluster, 6**T**-**nat**: polyhedral (**a**), ball-and-stick (**b**), and cluster (**c**) representation.

Zeolitic frameworks [122,123] can be built by topotactic stacking of layers, potentially leading to stacking faults and polytypism. The idealized model of tetragonal 1*TT*-polytype (Figure 6) with space group *P*-42*m*, which is characterized by one layer per unit cell, corresponds to the **EDI-**type framework topology (e.g., edingtonite, Ba[Al_2_Si_3_O_10_]∙4H_2_O [124], as well as kalborsite, K_6_[Al_4_Si_6_O_20_][B(OH)_4_]Cl [125,126], and arzamastsevite, K_6_[Al_5_Si_5_O_20_][Si(OH)_4_]Cl [127]). This framework corresponds to the following sequence of tiles: [4^3^]_2_[4^2^.8^2^]_2_[8^6^] (Table 2, Figure 7b). The orthorhombic polytype (Figure 6), with a doubled unit cell due to antiparallel stacking of polar layers, contains a framework of **THO**-type (e.g., thomsonite). Although the sequence of tiles in the **THO**-type framework is equal to that in the **EDI-**type framework ([4^3^]_2_[4^2^.8^2^]_2_[8^6^]), the local symmetry of [4^3^] and [8^6^] clusters is different, as well as the geometry of [4^2^.8^2^] tiles (which is represented by two geometric isomers with distinct four-ring orientations).

Polytypism generates frameworks with identical compositions but distinct pore geometries. More complex ordered structures (e.g., more than two layers per unit cell) are theoretically plausible.

#### 4.3.2. Hierarchical Building Units

Zeolitic frameworks can be dissected into the following hierarchical units:

1. *Primary Building Units* (PBUs): Vertex-linked *T*O_4_ tetrahedra.
2. *Secondary Building Units* (SBUs): Finite/infinite subunits (e.g., the 4 = 2 chain in **EDI**/**THO**/**NAT** frameworks [123]).
3. *Compositional Building Units* (CBUs): Recurrent clusters revealing structural homology.

**Figure 8:**
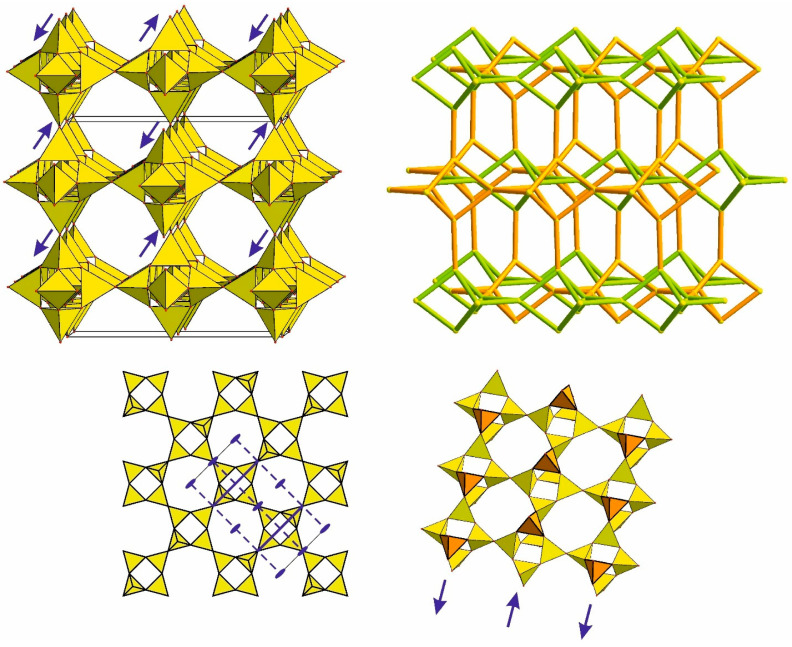
Features of the topology of the **NAT**-type framework constructed by the condensation of mountainite-type layers according to the gunterblassite principle (via “additional” tetrahedra). General view of the idealized structure with different orientations of 6**T**-**nat**. CBUs are highlighted by green lines, while the symmetry features of the mountainite-type layers are shown in dark blue.

In **EDI**/**THO**/**NAT** frameworks, the **6T-nat** CBU (a six-tetrahedron natrolite-like cluster) dominates (Figure 7). The **NAT**-type (natrolite) framework shares fundamental similarities with the **EDI** (edingtonite) and **THO** (thomsonite) framework types, as all three arise from the condensation of [4^1^8^1^] nets composed of four- and eight-membered rings through the incorporation of additional [*T*O_4_] tetrahedra (following the günterblassite principle). However, detailed analysis reveals essential differences in their formation mechanisms and resulting topologies. While **EDI**/**THO** framework types are derived from shlykovite-type layers, the **NAT** framework forms through condensation of mountainite-type layers (Figure 1d). This distinction is pivotal, as mountainite layers contain tetrahedra with mixed **u**/**d** orientations (inverted and non-inverted), unlike the uniform orientation in shlykovite layers (Figure 8). The alternating **u**/**d** arrangement of tetrahedra in mountainite layers introduces asymmetric connectivity patterns in eight-membered rings and altered pore geometry (elliptical vs. circular channels). Despite these differences, the **NAT**-type framework retains a three-tile sequence: [4^3^][4^2^.8^2^][8.9^2^]_2_ (Table 2).

#### 4.3.3. Main Types of Zeolite Frameworks Based on Apophyllite Nets

The **EDI**, **THO**, and **NAT**-type frameworks are characterized by systems of intersecting channels extending along coordinate directions. In the **EDI** and **THO** structures, these channels have an octagonal cross-section, while in the **NAT** structure, they exhibit both octagonal and nonagonal cross-sections. The intersections of these channels form large cavities of the topologies [8^6^] (**EDI**, **THO**) or [8^4^9^2^] (**NAT**). According to the rules of the International Zeolite Association (IZA) [2,128], the channel systems can be represented as {3[8^6^]<100>(8-ring), [001](8-ring)} for **EDI**; {3[8^6^][100](8-ring), [010](8-ring), [001](8-ring)} for **THO**; and {3[8^4^9^2^]<100>(8-ring), [001](9-ring)} for **NAT** [123], where square brackets indicate the channel direction, and angle brackets denote that the framework channels extend simultaneously along symmetrically equivalent coordinate directions [56]. The effective channel widths (*d_i_*) [2,128] in idealized frameworks [123] are presented in Table 3.

**Figure 9:**
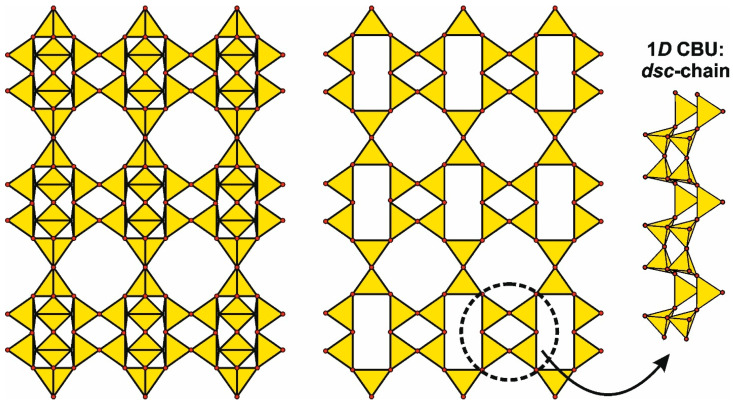
Hypothetical tetrahedral framework obtained through the direct topotactic combination of rhodesite-type double-layers.

The topology of a hypothetical model obtained by direct stacking of double-layer rhodesite-type packets (Figure 9) [119] was also analyzed. It consists of four types of tiles: [4^2^.8^2^][4^4^.6^2^][8^6^][4^4^.6^2^.8^2^]. The compositional structural unit of such a framework is a dsc-chain (“double sawtooth chain”), analogous to the unbranched three-membered double chain {***uB***,2^1^_∞_}[Si_6_O_15_] [4] in the crystal structure of epididymite, Na_2_Be_2_[Si_6_O_15_]}∙H_2_O [130]. The most similar tetrahedral frameworks in terms of topology are the **ATT-** and **OWE**-types, first identified in the structures of the compounds AlPO-12-TAMU, **|**(TMA^+^)_2_(OH)_2_**|**_2_**[**Al_6_P_6_O_24_**]**_2_ (TMA—tetramethylammonium ion) [131], and UiO-28, **|**(DETA)_2_(H_2_O)_2_**|**_2_**[**Mg_2_Al_6_P_8_O_32_**]**_2_ (DETA—diethylenetriamine) [132], respectively.

### 4.4. Multilayer Polyanions Based on Seidite-Type Layers

Despite the fact that nets with apophyllite-type topology, specifically the seidite-type stereoisomer, have not yet been observed as isolated single-layer polyanions, numerous multilayer representatives (primarily framework structures) formed by their condensation allows us to trace the formation features of these polyanions, as well as their polymorphism and topology.

Similarly to the case with shlykovite-type layers, hypothetical isolated single-layer seidite-type structures are characterized by the general formula [*T*_4_O^br^_6_Ø^ap^_4_]*^n^*^−^. However, due to stereoisomerism, the ratio of “bridging” to “terminal” vertices differs in multilayer polyanions as compared to compounds built from shlykovite- and mountainite-type layers. For instance, the formula of a double-layer polyanion in seidite-(Ce), Na_4_(Ce,Sr)_2_TiSi_8_O_18_(O,OH,F)_6_·5H_2_O [108], is [*T*_8_O^br^_14_Ø^ap^_4_]*^n^*^−^ = [*T*_8_Ø_18_].

**Figure 10:**
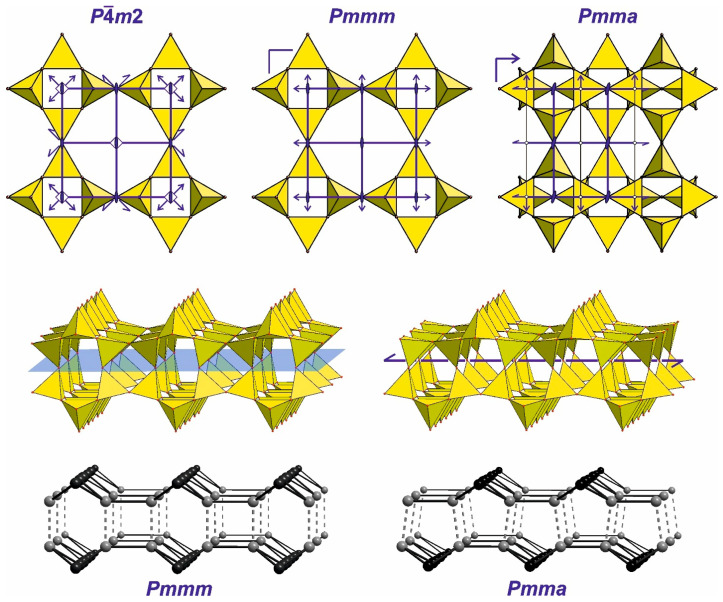
The symmetry of the seidite-type stereoisomer single layer (*P*-4*m*2) and the possible types of direct linkages of the layers with the formation of double layers of different symmetry (*Pmmm* or *Pnma*).

As with double-layer rhodesite-type polyanions, there are two fundamentally different ways of combining seidite-type layers, resulting in double-layer polyanions differing not only in symmetry but also in topology. In the first case, the double-layer polyanion is formed by reflection of the original seidite layer across a symmetry plane (symmetry of the double layer is *Pmmm*) (Figure 10). In the second case, the layers are related by a twofold screw axis, leading to a relative shift (the symmetry of the double layer is *Pmma*) (Figure 10). In both cases, the symmetry is reduced from tetragonal *P*-42*m* (symmetry of the hypothetical single layer) to orthorhombic *Pmmm* or *Pmma*, due to the specific rotation of tetrahedra in the original layer.

Condensation of layers to form multilayer polyanions and framework structures under consideration here can occur in two of the following ways:

1. Sequential layer-by-layer stacking of seidite layers (with possible preservation and/or alternation of stacking types, leading to different polytypes).
2. Connection via an “additional” tetrahedron, analogous to the “günterblassite” mechanism of combining double-layer rhodesite-type layers.
3. A combination of mechanisms 1 and 2, leading to hybrid structures.

**Figure 11:**
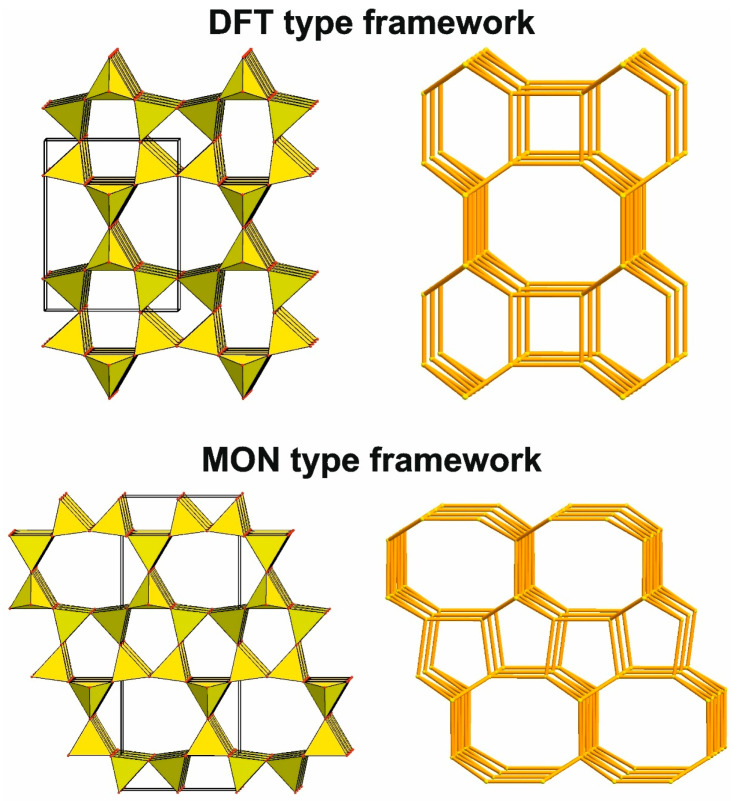
General views of the **DFT**- and **MON**-type frameworks (obtained by different symmetrical layer-by-layer stacking of seidite-type layers) and their topologies.

Examples of framework structures include zeolite-like materials with **DFT** and **MON** topologies (mechanism 1), **NAB** (mechanism 2), and **LOV**, **RSN**, and **VSN** (mechanism 3). The tetrahedral framework with **DFT**-type topology (named after the compound DAF-2, (C_2_H_10_N_2_)_2_[Co_4_P_4_O_16_] [133]), is formed by condensing seidite-type layers via a mirror plane (Figure 11), while the **MON**-type (the zeolite montesommaite, (K,Na)_9_[Al_9_Si_23_O_64_]∙10H_2_O [134]) is related via a twofold screw axis (Figure 11). This symmetry relationship allows these frameworks to be considered as polytypes of each other, similar to **EDI** and **THO**. The stacking difference is reflected in their distinct topologies: [4^2^.6^2^][6^2^.8^2^][4^2^.8^4^] for **DFT** and [4.5^2^][5^2^.8^3^] for **MON** [123]. The sequence of tiles shows a significant topological difference, as there are no common tiles or closely related stereoisomers between the two frameworks. Both frameworks feature wide channels. Due to the layer-by-layer condensation of seidite layers related by a mirror, the **DFT**-type framework has three channel systems along coordinate directions. Channels along the *a* and *b* axes are interwoven, as they are symmetrically equivalent and related by a 4_2_ screw axis, while each intersects with a channel along the *c* axis. A simplified notation of these frameworks can be written as {2[4^2^8^2^]<100>(8-ring), 1[4^2^8^2^][001](8-ring)} [135].

In contrast, the **MON**-type framework, {1[5^2^8^1^8^2/2^]<100>(8-ring)}, lacks a channel along the *c* axis due to a shift between the seidite layers, and the channels along *a* and *b* related by a 4_1_ screw axis are offset and do not intersect [135].

As noted above, apophyllite-type single layers can combine not only directly but also via “additional” tetrahedra inserted between single layers or multilayer polyanions (the “günterblassite” mechanism). Unfortunately, no finite multilayer polyanions of this type have been discovered. However, tetrahedral frameworks built based on this principle are known.

**Figure 12:**
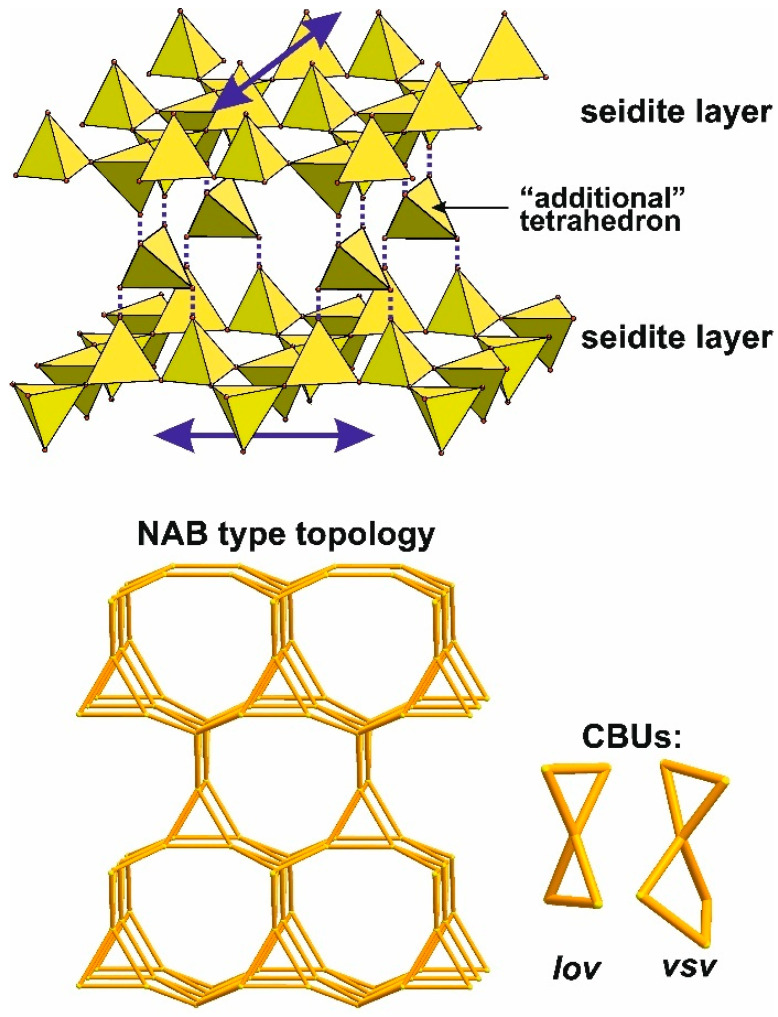
The **NAB**-type framework of tetrahedra, which contains single seidite-type layers connected via an “additional” tetrahedron, and its topological features.

The **NAB**-type framework in nabesite, Na_2_{Be(Si_4_O_10_)}(H_2_O)_4_ [136,137,138], contains single seidite-type layers connected via an “additional” tetrahedron (Figure 12), causing adjacent layers to rotate by 90° relative to each other. The topology of this framework includes the following tiles, [9^4^][3^2^.4.8.9^2^]_2_, and its compositional building units (CBUs) are 5*T*-*lov* and 6*T*-*vsv*, containing three- and four-membered rings (Figure 12). The framework features intersecting channel systems along all coordinate directions: {3[3^3^8^1^9^4^]<100>(8-ring),[001](9-ring)} [135].

**Figure 13:**
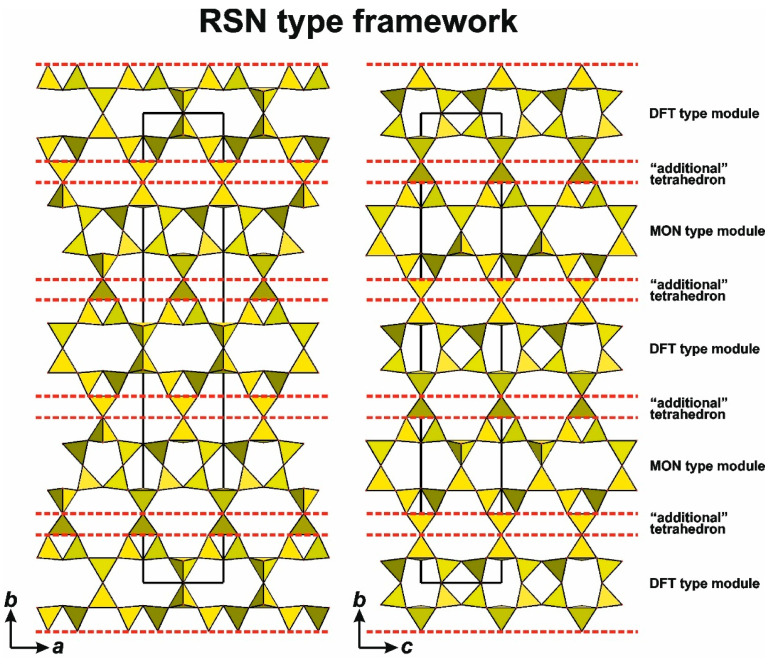
The tetrahedral **RSN**-type framework, which contains alternating modules of the **DFT-** and **MON**-types, linked via additional tetrahedra.

Frameworks of the **LOV**-type (in lovdarite, K_4_Na_12_(Be_8_Si_28_O_72_)·18H_2_O [138]) and **VSV**-type (in the compound VPI-7, Na_32_(Zn_16_Si_56_O_144_)·40H_2_O [139]) are formed by connecting double-thick seidite layers via additional tetrahedra (in **LOV**, double layers are related by a mirror plane; in **VSV**, by a twofold screw axis). A particularly interesting hybrid is the **RSN**-type framework (compound RUB-17, K_4_Na_12_(Zn_8_Si_28_O_72_)·18H_2_O [140]), where double layers of both types (mirrored and screw-related) are connected via additional tetrahedra. Thus, the **RSN** framework contains alternating modules of the **DFT-** and **MON**-types interspersed with “additional” tetrahedra (Figure 13).

For all frameworks built by seidite layers connecting via additional tetrahedra, the CBUs are 5*T*-*lov* and 6*T*-*vsv*, clusters that are similar to those in the prototype **NAB** framework (Figure 12). A comparative analysis of the different framework types is shown in Table 3.

### 4.5. Frameworks Based on Apophyllite-Type Layers

The cubic framework (space group *Im*-3*m*) with **ACO**-type topology (the compound ACP-1, (C_2_H_10_N_2_)_4_(Al_0.88_Co_7.12_P_8_O_32_)·2H_2_O [141]) is constructed through the layer-by-layer stacking of apophyllite-type layers. Due to the identical orientations of tetrahedra in the four-membered rings of these layers, their stacking forms a cubic cluster consisting of 8 tetrahedra (8*T*-*d*4*r*), which serves as the CBU of this framework (Table 3). A distinctive feature of this framework is that, despite having three types of channels extending along all coordinate directions, their arrangement is such that only two of them intersect with each other: {2[4^2^8^4^]<100>(8-ring)} [135].

### 4.6. Isolated Tetrahedral Layers and Frameworks Containing Apophyllite-Type Nets

Apophyllite-type nets are quite common in the structures of natural and synthetic compounds. Earlier, we discussed large families of structures formed by the polymerization of specific structural isomers of apophyllite-type layers of tetrahedra, which allowed us to identify crystal–chemical relationships both within and between these families. However, a significant number of compounds, whose structures contain single-, double-, or multilayer polyanions as well as tetrahedral frameworks, lack direct analogs and either only have topologically similar frameworks or identical layered polyanions. We examine such compounds in the following section.

#### 4.6.1. Double Layer Based on Carletonite-Type Layers and the Topology of a Hypothetical Framework

**Figure 14:**
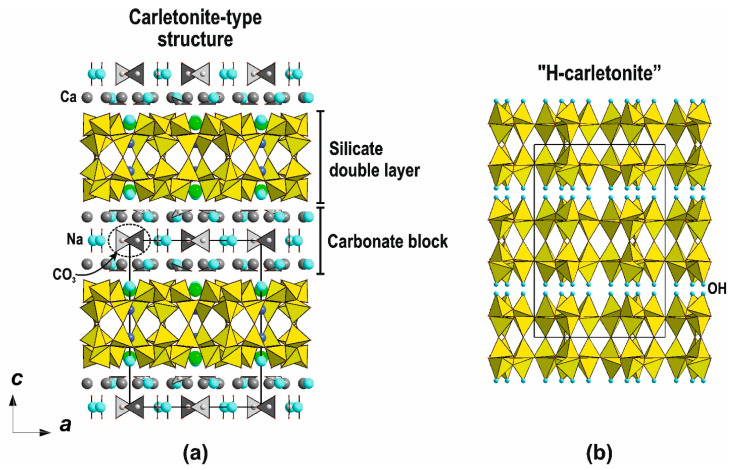
The general view of the crystal structure of carletonite-type structure (**a**) and crystalline silicic acids called “H-carletonite”, [Si_8_O_14_(OH)_4_] (**b**).

**Figure 15:**
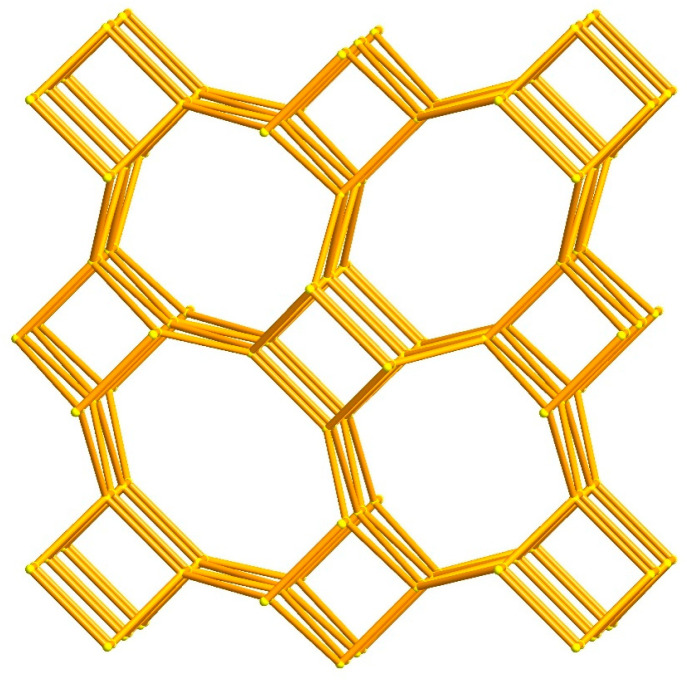
The topology of the hypothetical framework, obtained by the condensation of tetrahedral layers of the carletonite-type.

Tetrahedral nets characterized as the carletonite-type isomer (Figure 1) have not yet been observed as isolated anions. However, double layers (*n* = 2) formed by their combination via simple mirror symmetry have been found in the crystal structures of carletonite, KNa_4_Ca_4_[Si_8_O_18_](CO_3_)_4_(OH,F)·H_2_O [102] and fluorcarletonite, KNa_4_Ca_4_[Si_8_O_18_](CO_3_)_4_F·H_2_O [103] (Figure 14a) as well as crystalline silicic acids called “H-carletonite”, [Si_8_O_14_(OH)_4_] [142] (Figure 14b). Their stoichiometry is the same as that of a double layer formed from seidite-type layers [*T*_8_O^br^_14_Ø^ap^_4_]*^n^*^−^ = [*T*_8_Ø_18_]. Nevertheless, differences in tetrahedral orientations significantly affect their topological features, as is clearly seen in a hypothetical tetrahedral framework (with tetragonal symmetry, space group *I*4/*mcm*) obtained by layer-by-layer condensation of carletonite-type layers. The topology of this hypothetical framework (Figure 15) differs from the **MON**- and **DFT**-type frameworks and is represented by the tile sequence: [6^4^.8^2^][4^8^.6^4^.8^2^]. The framework’s CBU consists of “double crankshaft chains” (*dcc*-chains) (Figure 15). The framework features a system of parallel channels extending perpendicular to the layer-stacking direction (along the *c* axis): {1[6^4^8^2/2^][001](8-ring)}. Based on an optimized model of it, it was shown that the *e.c.w*. is approx. 3.3 Å × 3.3 Å [142].

#### 4.6.2. Frameworks Constructed by Combining Paracelsian-Type Layers

Composite building units in the form of *dcc*-chains also form paracelsian-type frameworks in the structure of paracelsian, Ba(Al_2_Si_2_O_8_) [143] (a feldspar group mineral [144]) and related compounds, including the borosilicate danburite Ca[B_2_Si_2_O_8_] [145] and its potassium analog, K[BSi_3_O_8_] [146], mixed zinc–gallium phosphates (e.g., (NH_4_)[(Zn,Ga)_2_P_2_O_8_] [147]), potassium lithioborophosphate, K_2_[LiBP_2_O_8_] [148], and beryllophosphates with the general formula *M*^2+^[Be_2_P_2_O_8_] (where *M*^2+^ = Ca, Sr, Pb, Ba) [149], including the mineral hurlbutite, Ca[Be_2_P_2_O_8_] [150]. The basis of this framework consists of apophyllite-type layers belonging to a structural stereoisomer not yet observed as an isolated polyanion. The specifics of tetrahedral orientation and distortions of this framework caused by extra-framework cations were analyzed [149].

**Figure 16:**
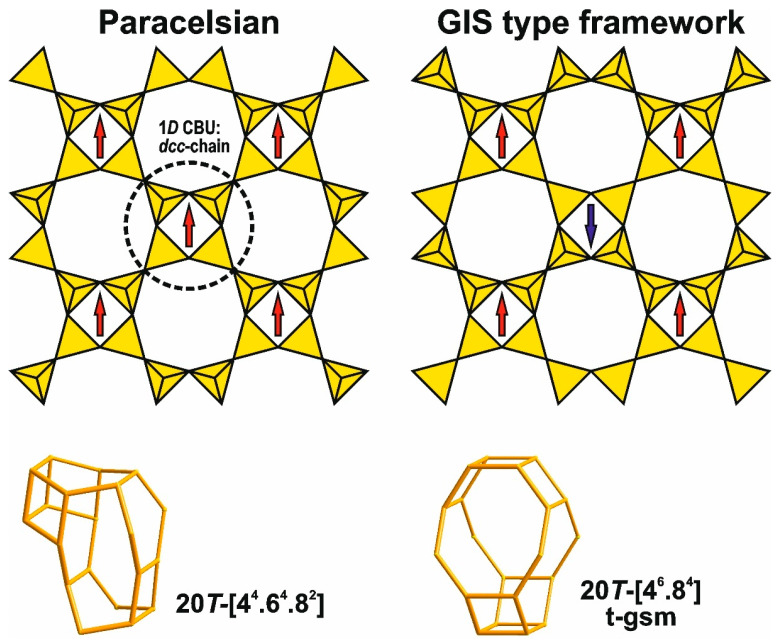
The topological relationship between paracelsian- and **GIS**-type tetrahedral frameworks represented by different orientations of the *dcc*-chains.

The topology of the paracelsian-type framework consists of a single tile type [4^4^.6^4^.8^2^] (Figure 16), closely resembling the 20*T*-*gsm*-type tile [4^6^.8^4^] (Figure 16) in **GIS**-type frameworks (the gismondine group: gismondine-Ca, gismondine-Ba, and gismondine-Sr [151,152,153,154]). Due to topological features (linked to the orientation of *dcc*-chains; Figure 16), the paracelsian-type framework has only one system of parallel channels {1[4^4^6^4^8^2/2^][001](8-ring)}, whereas the **GIS**-type framework has two intersecting channel systems: {1[4^6^8^4^][100](8-ring), [001](8-ring)} [135].

#### 4.6.3. Frameworks Constructed by Combining Phillipsite-Type Layers and Their Variants

**Figure 17:**
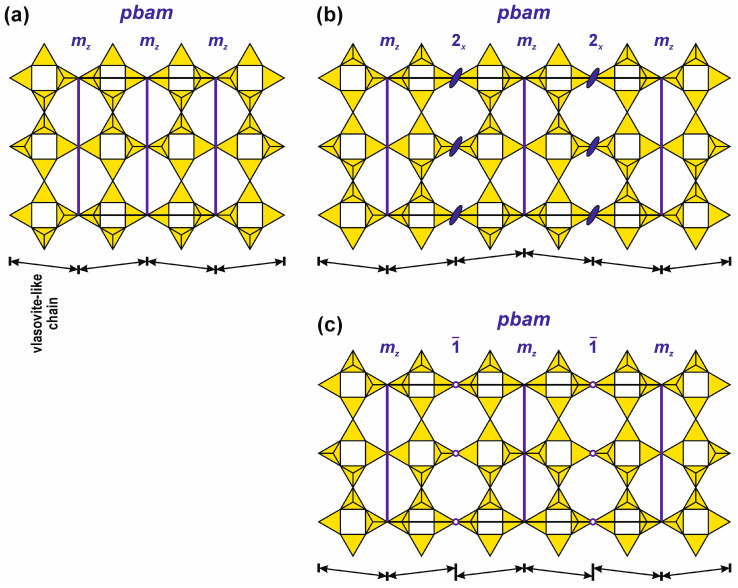
The polytypism of the apophyllite-type layer, depending on the active symmetry operations: the single tetrahedral layers in the **PHI**-type (**a**) and **SIV**-type (**b**) frameworks, and possible derivative layer (**c**). The symmetry operations are shown in dark blue.

Tetrahedral frameworks of the **PHI**-type are found in minerals containing a phillipsite group: phillipsite-Ca, Ca_3_[Al_6_Si_10_O_32_]·12H_2_O, phillipsite-Na, Na_6_[Al_6_Si_10_O_32_]·12H_2_O, phillipsite-K, K_6_[Al_6_Si_10_O_32_]·12H_2_O [155,156,157,158], flörkeite, K_3_Ca_2_Na[Al_8_Si_8_O_32_]·12H_2_O [159], and harmotome, Ba_2_[Al_4_Si_12_O_32_]·12H_2_O [160], as well as their synthetic analogs [141,161]. These frameworks contain fragments of apophyllite-type nets of tetrahedra characterized by an isomer not yet observed as an isolated layered polyanion (Figure 17). Epy layer can be obtained by condensing a loop-branched chain {***l*B**, 1^1^_∞_}[^6^*T*_8_Ø_22_] (with a periodicity of six tetrahedra [51], analogous to the vlasovite chain in the crystal structure of vlasovite, Na_4_Zr_2_{***l*B**, 1^1^_∞_}[^6^Si_8_O_22_] [162]), through sequential reflection in *m_z_* mirror planes (Figure 17a).

Alternating *m_z_* mirror planes with perpendicular 2*_x_* twofold axes results in an orthorhombic 2*O*_1_ polytype of this layer (Figure 17b), observed in the crystal structure of the compound SIZ-7 (Co_0.4_Al_0.6_)PO_4_ [163], characterized by the **SIV**-type topology. A hypothetical orthorhombic 2*O*_2_ polytype of the SIZ-7 layer can be generated by alternating *m_z_* planes with inversion centers (Figure 17c). Despite stereoisomeric differences, all layers share the same symmetry (*Pbam*), with varying tetrahedral rotations only affecting one unit cell parameter. Similar stereoisomerism variations in mica-like layers with [61] and partial [4^1^6^1^8^1^] topologies were previously discussed [164].

Differences in layer stereoisomerism significantly impact framework topology, though their relation is evident in shared tiles: [4^5^.8^3^][4^7^.8^5^] for the **PHI**-type, [4^5^.8^3^][4^6^.8^4^]_2_[4^7^.8^5^] for **SIV**-type, and [4^5^.8^3^][4^4^.6^2^8^2^][4^7^.8^5^][4^6^.6^2^.8^4^] for the hypothetical **SIV**-2*O*_2_ polytype. While **PHI** has intersecting channels {2[4^12^8^6^][100](8-ring), [001](8-ring)}, **SIV** features parallel channels {{1[4^12^8^4^8^2/2^][100](8-ring), 1[4^12^8^4^8^2/2^][100](8-ring)} [135]. The hypothetical **SIV**-2*O*_2_ polytype differs but retains two common tiles ([4^5^.8^3^] and [4^7^.8^5^]), confirming the topological relation despite variations in stereoisomerism.

#### 4.6.4. Frameworks Constructed by Combining Merlinoite-Type Layers

**Figure 18:**
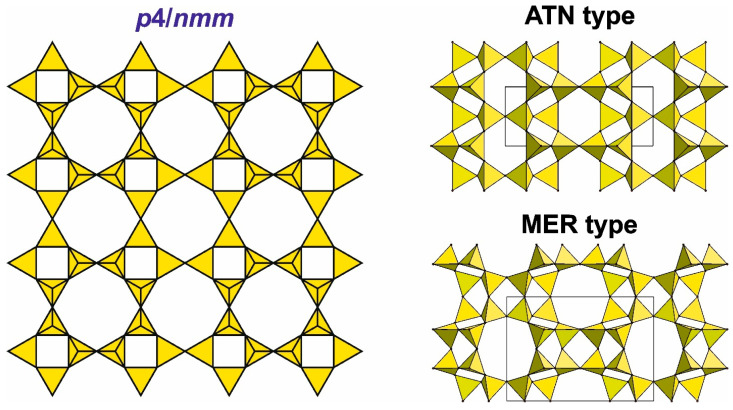
The single tetrahedral layer in the **ATN**-type and **MER**-type frameworks.

Frameworks with **ATN** (the compound MAPO-39, HAl_7_MgP_8_O_32_ [165]) and **MER** (merlinoite, (K,Na)_5_(Ca,Ba)_2_[Al_9_Si_23_O_64_]∙23H_2_O [166]) topologies can be formed by stacking layers exhibiting merlinoite stereoisomers (Figure 18), which has not been previously observed in single- or double-layer silicate structures (Figure 1, Table 1). The topological differences between the **ATN** and **MER** frameworks stem from distinct layer-stacking patterns (analogous to seidite): direct layer-by-layer stacking for **ATN** versus mirror reflection of the original layer for **MER**, with unit cells containing one and two layers, respectively. Although both frameworks share the same symmetry, their topologies differ significantly. Specifically, **ATN** and **MER** frameworks feature distinct tilings ([6^2^.8^2^]_2_[4^8^.6^4^.8^2^] and [4^2^.8^4^]_2_[4^8^.8^2^][4^12^.8^6^], respectively) that share no common tiles.

#### 4.6.5. Frameworks Constructed by Combining Cavansite-Type Layers and Hybrid Frameworks with Alternating Cavansite- and Pentagonite-Type Layers

**Figure 19:**
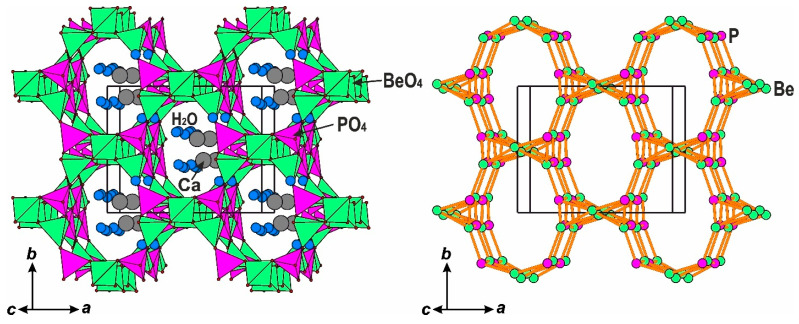
The crystal structure of weinebenite and the topology of its tetrahedral framework.

The mineral weinebenite, Ca_4_[Be_12_P_8_O_32_(OH)_8_)](H_2_O)_16_ [167], features a crystal structure (Figure 19) containing cavansite-type layers (Figure 1c) interconnected via the “günterblassite” mechanism through additional tetrahedra between adjacent layers. Unlike **NAB**-type frameworks, no 90° rotation occurs between neighboring layers.

**Figure 20:**
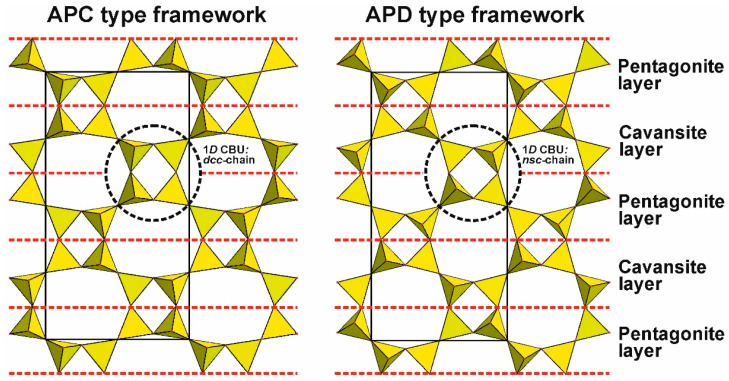
“Modular” frameworks of **APC**-type and **APD**-type, built up by tetrahedral layers of different stereoisomers.

Pentagonite, Ca(VO)[Si_4_O_10_]∙4H_2_O, is a cavansite dimorph [73,168,169] but incorporates mica-like tetrahedral nets (6^3^ topology). The tetrahedral orientation in pentagonite layers resembles that of cavansite layers (Figure 1), enabling their combination. In the **APC** (the compound AlPO-C, AlPO_4_; space group *Pbca*) and **APD** (the compound AlPO-D, AlPO_4_; space group *Pca*2_1_) [170] framework topologies, cavansite- and pentagonite-type layers alternate in a 1:1 ratio. The framework topology (APC or APD) depends on the stacking mode of these layers, with key differences manifesting in their CBUs: The **APC**-type frameworks contain *dcc*-chains (Figure 20), while APD-type frameworks feature unbranched four-membered narsarsukite-type double chains (*nsc*-chains) [51] (Figure 20).

#### 4.6.6. Influence of Dcc- and Nsc-Type CBUs on the Topology of Frameworks Containing Apophyllite-Type Nets

*dcc*-type chains form through mirror reflection of apophyllite-type layers containing pairs of similarly oriented tetrahedra in four-membered rings, whereas *nsc*-type chains arise from layers with oppositely oriented tetrahedra.

**Figure 21:**
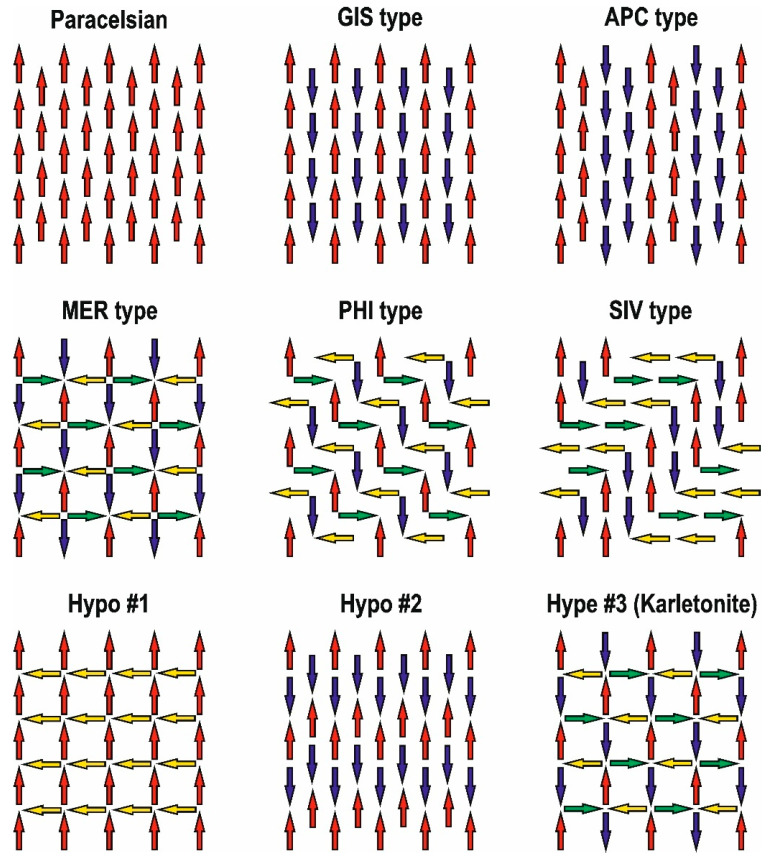
Schematic drawing of the building scheme used to construct the zeolite topologies in the family of dense double crankshaft topologies. Each arrow represents a *dcc*-chain, as projected along the chain direction, and the direction of the arrow indicates the relative rotational orientation.

As noted previously, paracelsian-type frameworks contain uniformly oriented *dcc*-chains (arrows in Figure 21) as their CBUs. **GIS-**, **APC-**, **MER-**, **PHI-**, and **SIV**-type frameworks also incorporate *dcc*-chains as CBUs, but with varying relative orientations. These framework differences, related to chain directionality, have enabled the prediction of potentially new tetrahedral framework types (Figure 21) [171]. Our detailed analysis of crystal structures containing apophyllite-type nets reveals that hypothetical frameworks with “Hypo #1” and “Hypo #3” [171] chain orientations would form through condensation of KNARSI- and carletonite-type layers, respectively (Figure 1g). This further confirms the feasibility of layer combination for framework anion formation and expands the possibilities for predicting novel topological types.

**Figure 22:**
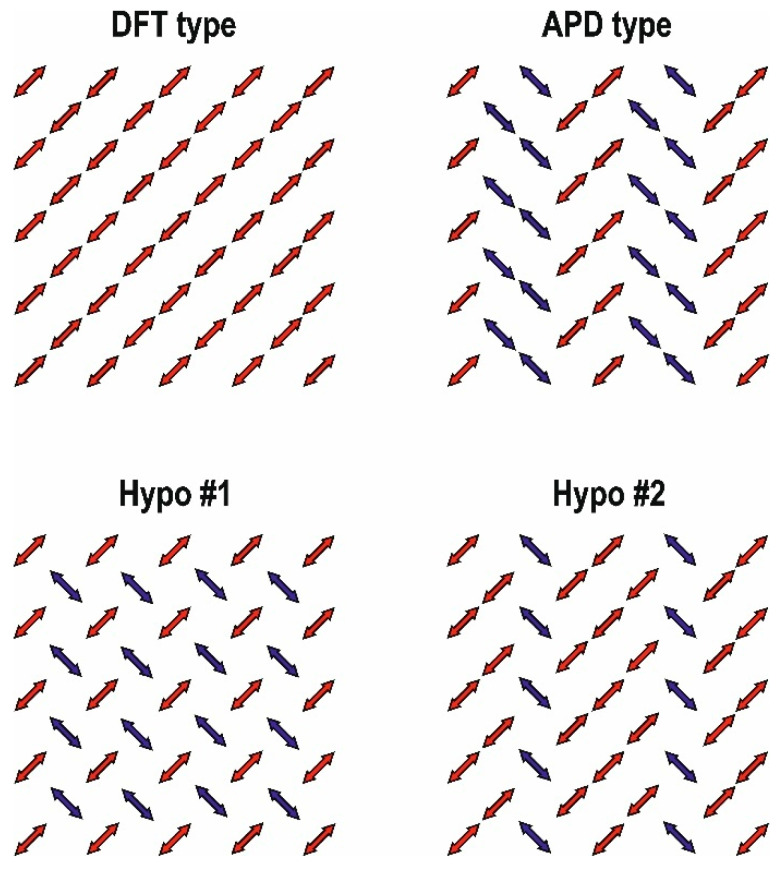
Schematic drawing of the building scheme used to construct the zeolite topologies in the family of narsarsukite topologies. Each arrow represents a *nsc*-chain, as projected along the chain direction, and the direction of the arrow indicates the relative orientation.

**The DFT**- and **APD**-type frameworks contain *nsc*-chains, with topological differences arising from their varying orientations (analogous to the *dcc*-chains discussed above). The **DFT**-type framework represents the simplest case, with all chains sharing identical orientation (Figure 22). More complex frameworks (e.g., **APD**-type) and hypothetical variants (Figure 22) can be derived following principles similar to those for *dcc*-type chains [171].

**Figure 23:**
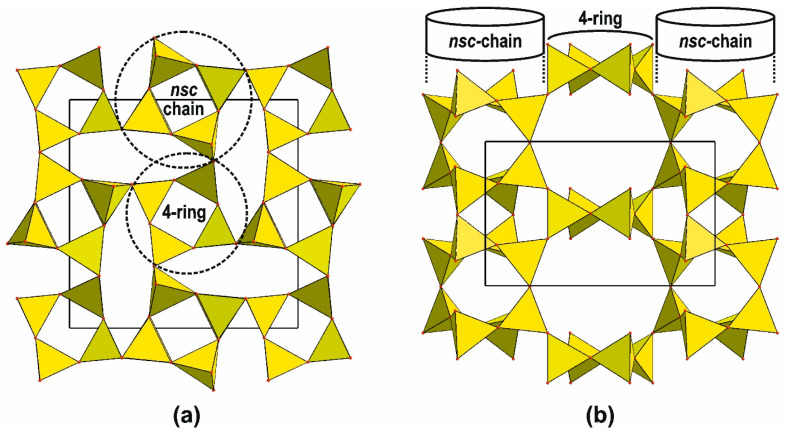
The general view of the tetrahedral framework in crystal structures of scapolite-group minerals (**a**) and the linkage of the adjacent *nsc*-chains by four-membered tetrahedral rings (**b**).

In the tetrahedral frameworks of the crystal structures of scapolite-group minerals [172,173] the adjacent distorted *nsc*-chains are linked via four-membered rings (Figure 23), forming the tetragonal–tetrahedral framework, related to **DFT**-type. However, despite the general similarity, the layers with apophyllite-type topology cannot be distinguished, due to the linkage type of the *nsc*-chains. The natural tiling of the tetrahedral frameworks in scapolite-group minerals consist of three natural tiles [174], [5^2^.8^3^]_4_[4^2^.5^4^]_2_[4^2^.8^4^], where the tile [4^2^.8^4^] is present in the framework of **DFT**-type, while the tile [5^2^.8^3^] is present in the heteropolyhedral *MT*-framework in the crystal structure of seidite-(Ce) [108] (see Section 7.3.1).

## 5. Polysomatism in Structures with Apophyllite-Type Nets

As noted above, compounds containing identical structural fragments, referred to as “recombination” structures [175], are currently described using the terminology of modular crystallography and mineralogy [49,176,177,178]. In their analysis of polysomatism in microporous phyllosilicates, G. Ferraris and A. Gula specifically identified the mero-pleisotypic rhodesite series [179]. A merotype series (from Greek *meros*, meaning “part”) means that all structures in the series share at least one invariant module, while the other(s) may vary between compounds. A pleisotype series (from Greek *plesio*, meaning “near”) means that all members contain the same number of topologically identical modules, though these may differ slightly (e.g., in chemical composition).

Ferraris and Gula proposed that compounds in this series are unified by a shared modular unit: a double-layer tetrahedral polyanion formed by combining apophyllite-type layers. Crucially, these polyanions can arise from pairs of layers belonging to different stereoisomers, i.e., isomers with distinct tetrahedral rotations and differing **ud**-matrices. Beyond the rhodesite-related minerals and their synthetic analogs, the mineral seidite-(Ce) [108,179] was later included in this series, as it satisfies the same modular criteria.

### 5.1. Polysomatic Series

The discovery of silicates containing three-layer polyanions, along with established structural relationships with framework structures, has provided new insights into the modular aspects of compounds built from apophyllite-type layers.

The extensive family of shlykovite-type layers now allows us to define not just a *mero-pleisotypic rhodesite series*, but rather a broader *edingtonite polysomatic series*. Members of this series are distinguished primarily by their degree of layer condensation (*n* = 1, 2, 3, …, ∞, where *n* is the number of layers in the tetrahedral polyanion). This polysomatic series includes the *merotypic rhodesite series* (shlykovite-type layers with *n* = 2), i.e., compounds exclusively featuring double tetrahedral layers.

**Scheme 1:**
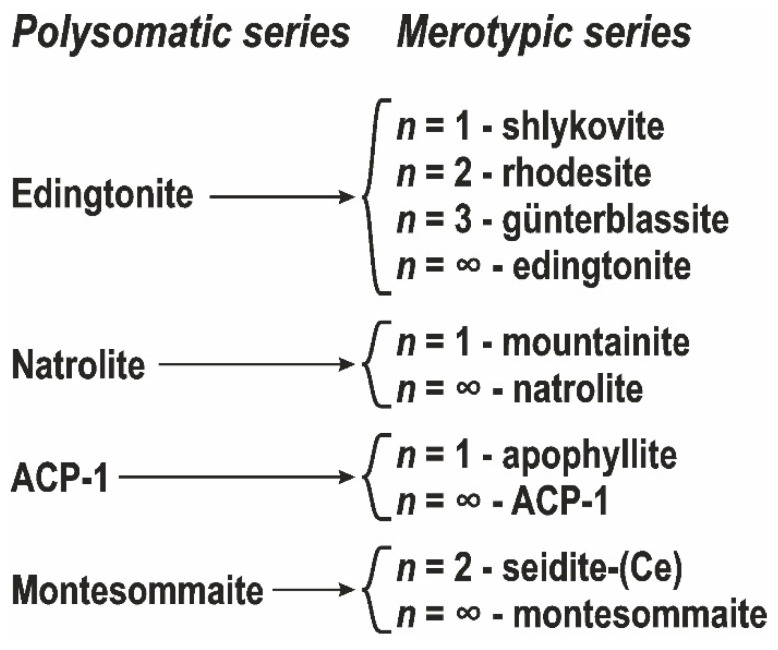
Current *polysomatic series* and their further subdivision into *merotypic series* based on the condensation degree (*n*) of apophyllite-type layers.

As demonstrated earlier, neither tetrahedral rotation can be treated as a random phenomenon, nor should compounds with layers belonging to different isomeric types be grouped together. In the case of finite framework members, this factor significantly influences topology. We suggest classifying compounds containing apophyllite-type layers (Scheme 1) based on the following:

1. Type of stereoisomer;
2. Number of layers in the tetrahedral anion.

A polysomatic series should be defined if, in addition to the endmember framework, at least one representative with a finite number of layers exists. Naming rules should be associated with the endmember structure or, in the case of polytypes, the simplest crystalline structure [180,181].

#### 5.1.1. The Edingtonite Polysomatic Series

The discovery of shlykovite and cryptophyllite [109,117] revealed that their structures are based on single layers of tetrahedra (*n* = 1) characterized by the same stereoisomer as the double layers of tetrahedra (*n* = 2) in rhodesite-related minerals and their synthetic analogs. The subsequent discovery of günterblassite [40,41] (as well as hillesheimite [121] and umbrianite [120]), which contain triple tetrahedral layers (*n* = 3) in their structures, demonstrated the possibility of further condensation of shlykovite-type layers to form multilayered polyanions composed of tetrahedra (*n* → ∞). The final stage of layer condensation is the formation of a framework of either the edingtonite (**EDI**) or thomsonite (**THO**) type [53].

Thus, the polysomatic edingtonite series includes a large number of structural varieties, and the increase in the degree of condensation of layers within the series can be represented as an alternation of shlykovite layers (*Shl*) with additional tetrahedra (*T*^add^), as follows:

(9) $$\left(Shl\right)\to {\left(Shl\right)}_{2}\to \left[{\left(Shl\right)}_{2}\left(Shl+T^{add}\right)\right]\to {\left[{\left(Shl\right)}_{2}{\left(Shl+T^{add}\right)}_{m}\right]}_{\infty \infty }={\left[{\left(Shl\right)}_{m+2}T_{m}^{add}\right]}_{\infty \infty }.$$

Using the degree of condensation (*n*), the entire polysomatic series can be expressed as follows:

(10) $${\left[{\left(Shl\right)}_{n}T_{m}^{add}\right]}_{\infty \infty },$$

where 1≤n<∞; *m* = *n* − 2, and m≥0.

In the framework structures of the **EDI**- and **THO**-types (when *n* = ∞), the ratio between shlykovite layers and additional tetrahedra is 1:1, thus (10) takes the form

(11) $${\left[\left(Shl\right)T^{add}\right]}_{\infty \infty \infty }.$$

Substituting the actual stoichiometry into (9), we obtain the following:

(12a) $$\left[T_{4}O_{10}\right]\to \left[T_{8}O_{19}\right]\to \left[T_{8}O_{19}+{\left(T_{4}O_{10}+T^{add}\right)}_{m}\right]\to \left[T_{4m+8}O_{10m+19}\right]$$

or, in a simplified form, for *n* > 2, we obtain the following:

(12b) $$\left[T_{4n}O_{10n-1}T_{m}^{add}\right]=\left[T_{4n+m}O_{10n-1}\right],$$

where 2<n<∞; *m* = *n*−2, and m≥0.

For finite-layered tetrahedral polyanions with 2 < *n* < ∞ belonging to the edingtonite polysomatic series, the number of apical Ø^ap^ vertices is always equal to 6. Moreover, in addition to the possibility of these Ø^ap^ vertices being partially or fully protonated, they form a triangular platform suitable for the attachment of external tetrahedra, thereby creating branched-type layers [51]. In this case, (9) takes the following form:

(13a) $$\left(Shl+T_{k}^{out}\right)\to \left[{\left(Shl\right)}_{2}+T_{k}^{out}\right]\to \left[{\left(Shl\right)}_{2}\left(Shl+T^{add}\right)+T_{k}^{out}\right]\to {\left[{\left(Shl\right)}_{m+2}T_{m}^{add}T_{k}^{out}\right]}_{\infty \infty }$$

or

(13b) $$\left[{\left(Shl\right)}_{n}T_{m}^{add}T_{k}^{out}\right],$$

where 2<n<∞; *m* = *n* − 2, and m≥0; 0≤k≤2.

The crystal structure of mountainite [101] established the endmembers of the natrolite polysomatic series. Unfortunately, no intermediate members of this series with a finite number of layers have been discovered to date. However, based on the relationship between mountainite and shlykovite (cryptophyllite), as well as the topological similarities between the **EDI**(**THO**)- and **NAT**-type frameworks, it can be assumed that this series is analogous to the edingtonite series. To understand the condensation of mountainite-type layers, it is sufficient to replace the shlykovite (*Shl*) layer with the mountainite (*Mau*) layer in (9) and (13a).

#### 5.1.2. The Montesommaite Polysomatic Series and Other Hypothetical Series Based on the Condensation of Seidite-Type Layers

Despite the existence of two different possible mechanisms of stacking adjacent layers (layer-by-layer and/or via additional tetrahedra), only one representative with a finite-layered polyanion formed by the condensation of seidite-type layers, namely, seidite-(Ce), is currently known [108]. Its structure consists of a double silicate layer (i.e., *n* = 2) formed by the stacking of layers with a shift due to the action of a twofold screw axis. If this stacking mode is maintained, the endmember of such a polysomatic series would be a framework of the **MON** type (Figure 11). Since the orientation of tetrahedra in a single layer of the seidite (*Sei*) isomer allows direct layer combination, the montesommaite polysomatic series (where layers are combined via a 2_1_ screw axis) can be written as follows:

(14) $$\left(Sei\right)\to {\left(Sei\right)}_{2}\to {\left(Sei\right)}_{3}\to {\left(Sei\right)}_{n},$$

where *n* → ∞.

Taking into account the actual stoichiometry, the scheme (14) can be presented as follows:

(15) $$\left[T_{4}O_{10}\right]\to \left[T_{8}O_{18}\right]\to \left[T_{12}O_{26}\right]\to \left[T_{4n}O_{10n-2(n-1)}\right]=\left[T_{4n}O_{8n+2}\right].$$

As with shlykovite-type layers, the stacking mode (via a mirror plane or a 2_1_ axis) does not affect the overall stoichiometry. Therefore, for the hypothetical DAF-2 polysomatic series (with a final framework member characterized by a **DFT**-type framework), the existence of the series (14) and (15) are expected. Thus, since these series cannot be distinguished by the stoichiometry of the tetrahedral anion alone, further subdivision based on the specific layer-stacking type seems unreasonable. Instead, newly discovered representatives should be classified under a joint montesommaite series. However, due to the significant influence of stacking methods on the topology of tetrahedral anions, it is possible to distinguish subseries (at a rank below the polysomatic series but above the merotypic series) based on local symmetry features.

If seidite layers are combined via “additional tetrahedra,” leading in the case of infinite condensation (*n* → ∞) to **NAB**-type frameworks, the sequence (14) can be rewritten as

(16a) $$\left(Sei\right)\to \left[{\left(Sei\right)}_{2}+T^{add}\right]\to \left[{\left(Sei\right)}_{3}+T_{2}^{add}\right]\to \left[{\left(Sei\right)}_{n}+T_{n-1}^{add}\right]$$

or

(16b) $$\left[T_{4}O_{10}\right]\to \left[T_{9}O_{18}\right]\to \left[T_{13}O_{26}\right]\to \left[T_{4n+n-1}O_{10n-2(n-1)}\right]=\left[T_{5n-1}O_{8n+2}\right],$$

resulting in a hypothetical polysomatic series that can provisionally be called the “nabesite polysomatic series.”

For hybrid structures, such as compounds with **LOV**-, **VSV**-, and **RSN**-type frameworks, where both layer-by-layer stacking and additional tetrahedra are involved, the schemes (16a,16b) can be written as

(17a) $$\left[{\left(Sei\right)}_{n}+T_{k}^{add}\right]$$

and

(17b) $$\left[T_{4n+k}O_{8n+2}\right],$$

where 0 < *k* < *n* − 1.

**Scheme 2:**
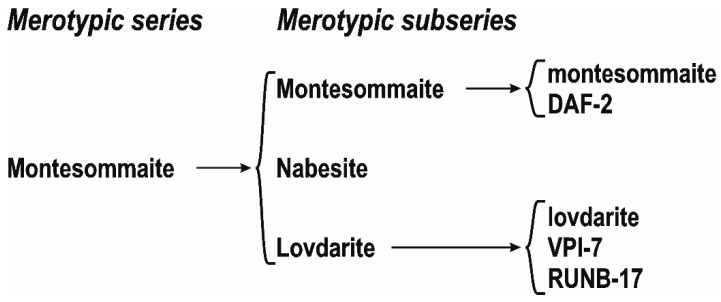
Polysomatic series and their further subdivision into subseries based on the condensation of seidite-type layers.

As with the montesommaite polysomatic series, we propose considering symmetry and topological features within separate subseries of a unified lovdarite polysomatic series. Thus, three parallel polysomatic series (two of which are currently hypothetical) can be distinguished based on the condensation of seidite-type layers (Scheme 2).

#### 5.1.3. The ACP-1 Polysomatic Series

A polysomatic series can also be defined based on the apophyllite-type layer (i.e., anapophyllite-type stereoisomer with the apophyllite-type topology) because both endmembers are known (though intermediates are still missing). By analogy with previous series, the condensation of apophyllite (*Aph*) layers can be presented as

(18a) $$\left(Aph\right)\to {\left(Aph\right)}_{2}\to {\left(Aph\right)}_{3}\to {\left(Aph\right)}_{n},$$

or, taking into account the stoichiometry, it can be presented as

(18b) $$\left[T_{4}O_{10}\right]\to \left[T_{8}O_{16}\right]\to \left[T_{12}O_{22}\right]\to \left[T_{4n}O_{10n-4(n-1)}\right]=\left[T_{4n}O_{6n+4}\right].$$

### 5.2. Complexity of Framework Structures

For quantitative assessment of crystal structures, it is convenient to use a complexity parameter based on Shannon’s information theory [180,181,182,183]. The amount of information per atom in a structure can be calculated using the following formula:

(19) IstructG=−∑i=1kpilog2pi,

where *p*_i_ is the probability of randomly selecting an atom from the *i*-th crystallographic orbit, given as

(20) $$p_{i}=m_{i}/v,$$

where *m_i_* is the multiplicity of the crystallographic orbit relative to the reduced unit cell, and *v* is the number of atoms in the reduced unit cell, given as

(21) $$v=\sum_{i=1}^{k}m_{i}.$$

The total information per unit cell can be obtained using the following equation:

(22) IstructG,total=−vIG=−v∑i=1kpilog2pi.

At the same time, increasing complexity of crystal structures makes a negative contribution to configurational entropy ($S\prime_{\mathrm{cfg}}$) [184]:

(23) $$S\prime_{\mathrm{cfg}}=k_{B}\left(\sum_{i=1}^{k}n_{i}\ln n_{i}-N\right),$$

where *n* is the number of atoms in the *i*-th crystallographic orbit, and *N* is the number of atoms in *k* crystallographic orbits (*k* > 1):

(24) $$N=\sum_{i=1}^{k}n_{i}.$$

Substituting (24) into (23) we obtain the following:

(25) $$S\prime_{\mathrm{cfg}}=S_{\mathrm{cfg}}^{max}-k_{B}NI_{G}ln2,$$

where $S_{\mathrm{cfg}}^{max}$ is the maximum configurational entropy for a crystal where all atoms are equivalent; *k*_B_ is the Boltzmann constant; and *N* is the number of atoms.

Previously, the complexities of all types of zeolite tetrahedral frameworks were calculated [110]. In general, they belong to compounds with simple (20 < *I_G_*_,total_ < 100 bits per unit cell) and intermediate (100 < *I_G_*_,total_ < 500 bits per unit cell) structural motifs. Increased framework complexity is observed in cases of polytypism: In the **EDI**→**THO** series, *I_G_*_,total_ values increase from 32.603 to 85.207 bits per unit cell, while in the **PHI**→**SIV** series, an increase from 140.078 to 368.156 bits per unit cell is observed. The **SIV**-type framework and its hypothetical 2*O*_2_-polytype have the same structural complexity value, as they are both characterized by identical unit cell parameters and space group. It should also be noted that despite the doubling of the **MON**-type framework cell compared to the **DFT**-type framework, they both have the same complexity (*I_G_*_,total_ = 46.039 bits per unit cell), which is related to increased symmetry, since the space group order of *P*4_2_/*mcc* (**MON**) is 16, while for *I*4_1_/*amd* (**DFT**), it is 32.

## 6. Crystal Chemistry of Compounds in the Edingtonite Polysomatic Series

Currently, the edingtonite polysomatic series includes natural and synthetic compounds containing single-, double- and triple-layered tetrahedral polyanions in their structures, as well as closed tetrahedral framework motifs characterized by the **EDI**- and **THO**-type topologies. Most of these compounds are minerals. Their mineralogical descriptions are given below.

### 6.1. Mechanisms of Combining Layered Tetrahedral Polyanions

**Figure 24:**
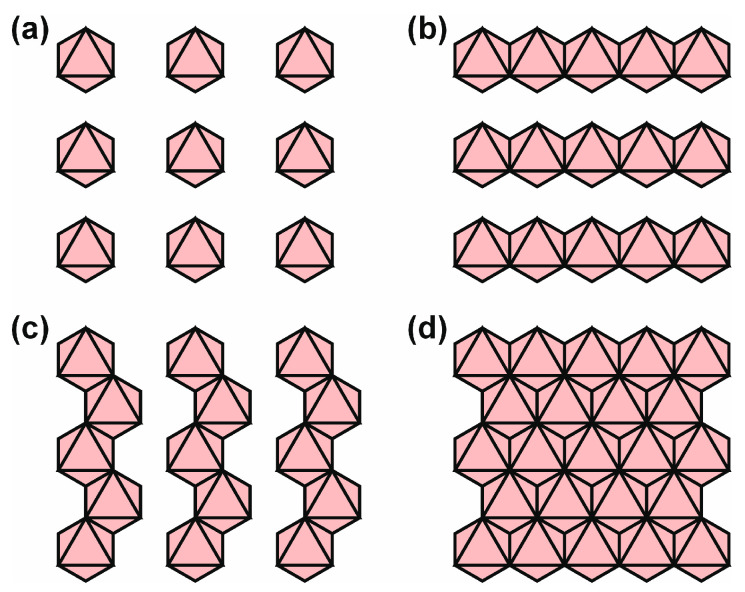
Central *O*-layer, represented by isolated octahedra (**a**), straight (**b**) and zigzag chains (**c**), and octahedral layer (**d**).

As shown above, layered tetrahedral polyanions with apophyllite-type topology can form both layered fragments of crystal structures and directly constitute tetrahedral frameworks. With significant silicon deficiency and excess of alkali, alkaline earth, and transition elements (characterized by octahedral coordination), layers of tetrahedra can alternate with layers formed by octahedra, creating three-layered *TOT* modules similar to those in mica-group mineral structures and their analogs [185]. The central layer in such modules may be built by densely packed (or partially vacant) octahedra or consist of isolated octahedra (Figure 24a), straight (Figure 24b), or zigzag (Figure 24c) chains of edge-sharing octahedra, and octahedral layers (Figure 24d).

**Figure 25:**
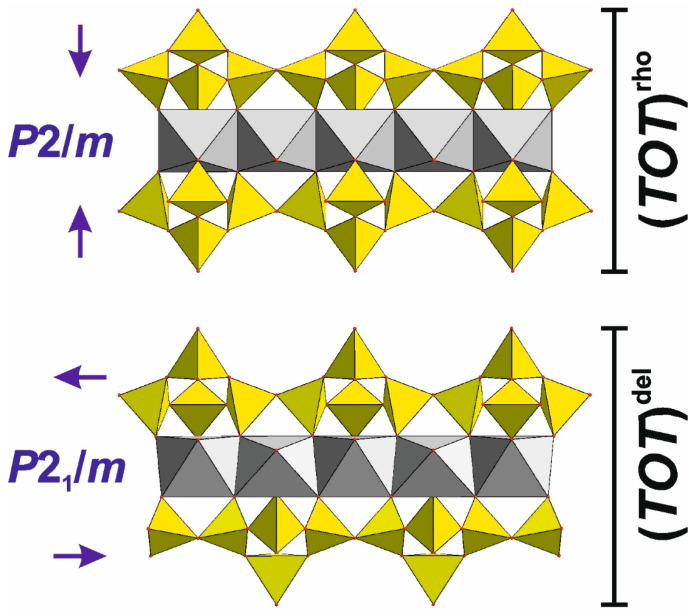
Two different possible ways of combining tetrahedral layers with octahedral ones.

In crystal structures (Table 4), there are two different ways that tetrahedral layers may be combined with octahedral layers [118], with shifts analogous to those for heteropolyhedral *H*-layers in *HOH* modules in heterophyllosilicate structures [49,87,186,187]. In the first case, layers of tetrahedra are aligned directly opposite each other, forming a three-layered (*TOT*)^rho^ module of the rhodesite-type (Figure 25). In the second case, tetrahedral sheets are offset, forming a (*TOT*)^del^ module of the delhayelite type (Figure 25). A detailed description of the symmetry features of the modules has been analyzed using the approach of OD (“order-disorder”) theory [118]. The space between such octahedral fragments (or voids within the layer) can be filled with large cations forming polyhedra with high coordination numbers.

### 6.2. Single-Layered Members: Shlykovite and Cryptophyllite

The single-layered “end-members” of this polysomatic series are represented by two minerals: shlykovite K_2_Ca_2_[Si_4_O_9_(OH)]_2_·6H_2_O and cryptophyllite K_4_Ca_2_[Si_4_O_10_]_2_·10H_2_O [109,117], hydrous potassium–calcium-layered silicates similar in chemical composition but differing in stoichiometry of the large cations (the Ca:K ratio is 1:1 in shlykovite and 1:2 in cryptophyllite), as well as water content. Both minerals were found in the Central apatite mine on Mount Rasvumchorr in the Khibiny alkaline massif (Kola Peninsula) [117,207]. The mineralogical descriptions of shlykovite and cryptophyllite are given in Appendix A.

**Figure 26:**
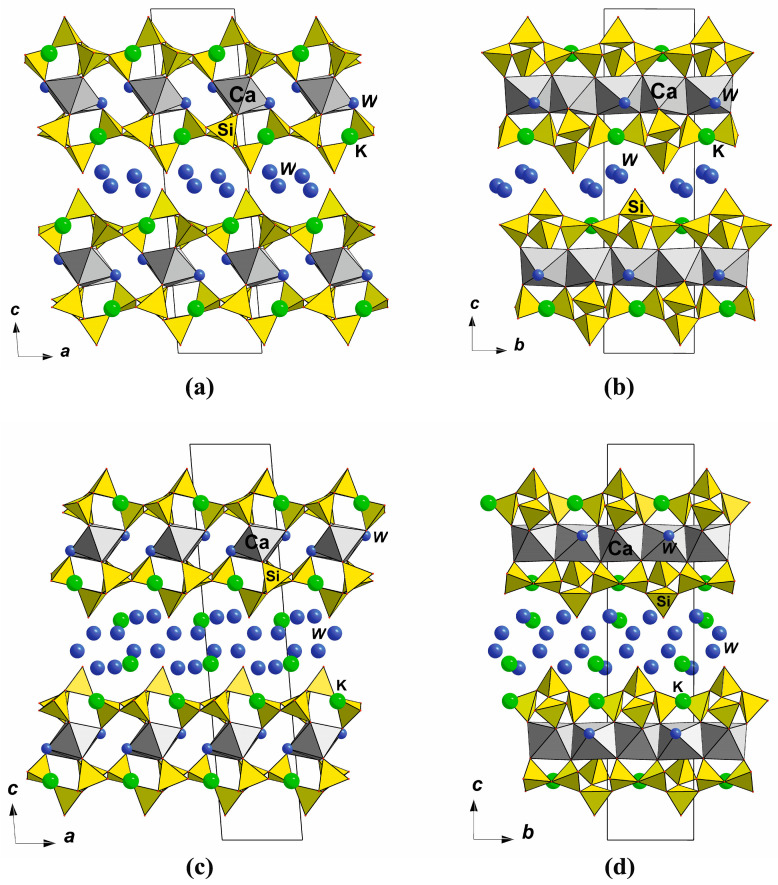
General views of the crystal structures of shlykovite (**a**,**b**) and cryptophyllite (**c**,**d**).

The crystal structures of shlykovite and cryptophyllite (Figure 26) are related and consist of three-layered delhayelite-type packets [(*TOT*)^del^ modules] [109,117,208], where the outer *T* (tetrahedral) layers are tetrahedral silicate sheets of the shlykovite-type. In cryptophyllite, it has the composition [Si_4_O_10_]^4−^_∞∞_, while in shlykovite, one of the ten oxygen atoms (the “outer” vertex of a **u**-configured tetrahedron, not involved in Si–O–Si or Si–O–Ca bridging) is protonated to form an OH group. Thus, the composition of its layer is [Si_4_O_9_(OH)]^3−^_∞∞_. The central *O* (octahedral) layer consists of straight chains of edge-sharing [CaO_5_(H_2_O)]-octahedra parallel to (010) (which represent fragments of a densely packed octahedral layer with composition (*M*Ø_2_)). The ratio between filled and vacant octahedral chains is 1:1. The identical structure of *TOT* packets in shlykovite and cryptophyllite results in very similar values of the *a* and *b* parameters and β angle of their unit cells. The *TOT* modules are connected via potassium cations and water molecules located in the intermodular space. In both minerals, potassium cations lie in the plane of the tetrahedral layer at the centers of eight-membered rings. Water molecules coordinate calcium in the octahedral O-layer and also occupy intermodular space. The excess potassium, together with additional water molecules in cryptophyllite, accumulates in the intermodular space. These differences in potassium and water content account for the substantial (5.4 Å) difference in the *c* parameter of shlykovite (26.7 Å) and cryptophyllite (32.1 Å), and consequently in their powder X-ray diffraction patterns.

### 6.3. Double-Layer Silicates: The Rhodesite-Related Minerals

The crystal structures of single-layer representatives of the shlykovite–edingtonite merotypic series are based on a three-layer *TOT* module, which serves as a fundamental building unit (**FBU** [123]). The free apical vertices of the [SiØ_4_]-tetrahedra create the prerequisites for the combination of adjacent *TOT* modules, forming a framework structure containing double-layer tetrahedral silicate polyanions that are interconnected through octahedral *O*-layers. In the rhodesite-related compounds, the *O*-layers are either dense or vacancy-containing (similar to layers in tri- and dioctahedral micas, respectively) and consist of edge-sharing octahedra or fragments of these layers (straight and zigzag chains of edge-sharing octahedra or discrete single octahedra) [179].

**Figure 27:**
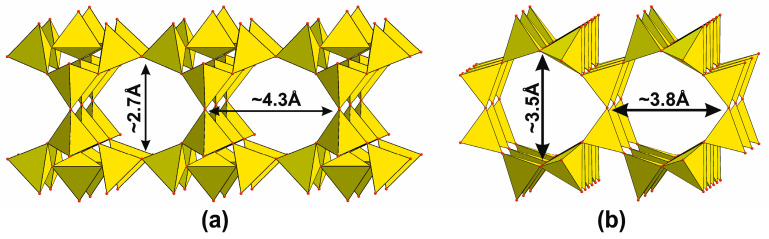
The systems of interpenetrating channels with eight-membered cross-section rings in tetrahedral double-layer structures of the rhodesite-type: channel **I** (**a**) and channel **II** (**b**).

The double tetrahedral layer [*T*_8_Ø_19_] is characterized by two systems of mutually penetrating channels extending along the two short unit cell parameters, either parallel or perpendicular to the rows of edge-sharing octahedra in the *O*-layer. Cross-sectionally, these channels are formed by eight-membered rings. The first type (Channel **I**) has a rounded cross-section with an effective channel width [2,209] diameter of 3.5–3.8 Å (Figure 27a), while the second type (Channel **II**) has an elliptical cross-section with dimensions of 2.7 × 4.3 Å (Figure 27b). The channels accommodate large cations and water molecules. The double tetrahedral layer exhibits configurational stability, and only the presence of a large cation in the *O*-layer (e.g., Sr^2+^) affects its configuration, compressing the channels along [001] [200].

In Hess et al. [116], the following general formula was proposed to describe the two-layered members of the rhodesite merotypic series:

H^[2]^_4−*v*_*A*^[6]^_2_*B*
^[6]^_2_*C*^[~8]^_0…4_*D*^[~10]^_2−*w*_{2^2^_∞_}[*T*^[4]^_8_O_19_]_2_*E*^[≥10]^_4−*x*_*F*^[≥10]^_4−*y*_*G*^[≥10]^_4−*z*_, (26)

where *A* and *B* are octahedrally coordinated medium-sized cations with charges of 1+, 2+, or 3+ and ionic radii *r*_[6]_ in the range of 0.90–1.05 Å (e.g., Ca^2+^ in rhodesite, delhayelite, fivegite, hydrodelhayelite, and macdonaldite, and Y^3+^ and Na^+^ in monteregianite-(Y)); *C* represents eight-coordinated cations of mono- and divalent elements with an ionic radius *r*_[8]_~1.2 Å (such as Na^+^, Ca^2+^, Sr^2+^); *E* includes mono- and divalent cations, while *D*, *F*, and *G* are monovalent cations, large anions like Cl^−^ or water molecules filling the channels of the double-layer tetrahedral polyanion. This formula is generally too detailed and has never been used for the systematic classification of known representatives or the description of new ones. Moreover, the formula (26) accounts only for chemical diversity, ignoring possible shifts in the double-layer polyanions relative to the octahedral *O*-layer due to polytypism and/or local disorder in layer stacking [118].

Since structural variations appear more significantly, we propose dividing the rhodesite-related compounds (containing double silicate layers) into two structural families (rhodesite and delhayelite ones) based on similar but symmetrically distinct (*TOT*)^rho^ and (*TOT*)^del^ modules, respectively (Figure 25).

#### 6.3.1. The Rhodesite Structural Family

The crystal structures of minerals and synthetic compounds belonging to the rhodesite structural family are based on three-layer (*TOT*)^rho^ modules with local symmetry *P*2/*m*, in which tetrahedral layers aligned to the central octahedral layer are oriented opposite to each other. Among two-layer silicates, this family contains the largest number of representatives, both natural and synthetic.

Rhodesite, KCa_2_[Si_8_O_18_(OH)]·6H_2_O, a white fibrous mineral, was named after Cecil John Rhodes, founder of the British mining company and Rhodes University in Grahamstown, South Africa, where the mineral was first studied [210]. It was later found in other regions, including the USA [211], Germany [116,212,213], Austria [214], and Italy [215].

The crystal structure of rhodesite was first solved in 1987 [213], and its detailed description based on three samples was published in 1992 [116] (after the structures of macdonaldite [188], hydrodelhayelite [205], and monteregianite-(Y) [189] had already been solved). However, as early as 1957, it was hypothesized (based solely on its unit cell parameters) that rhodesite is related to fibrous zeolites, particularly thomsonite-Ca, NaCa_2_[Al_5_Si_5_O_20_]∙6H_2_O [216].

**Figure 28:**
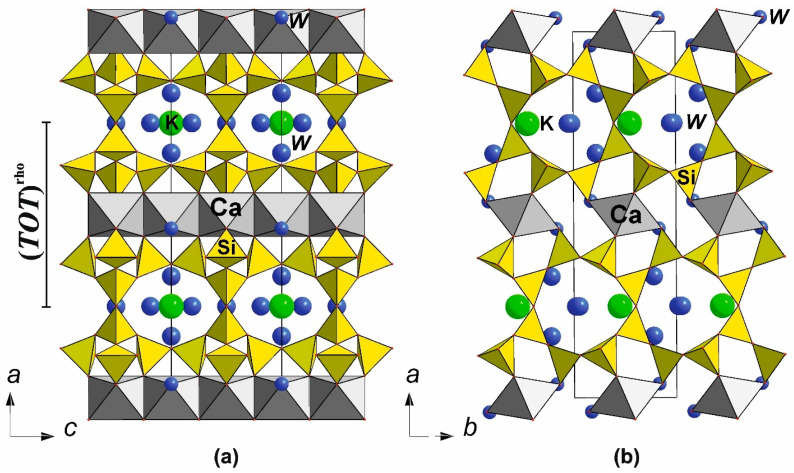
The general view of the crystal structure of rhodesite, projected onto (010) (**a**) and (001) (**b**).

In the crystal structure of rhodesite (Figure 28), the octahedral *O*-layer of the (*TOT*)^rho^ module consists of columns of edge-sharing CaØ_6_-octahedra with some vertices being protonated and corresponding to OH groups or water molecules. Adjacent (*TOT*)^rho^ modules are interconnected via common vertices of tetrahedra, forming a double tetrahedral layer of the rhodesite type and creating a heteropolyhedral framework. The wide channels in the tetrahedral layer are occupied by potassium atoms and water molecules.

Following the recommendations of the International Zeolite Association (IZA) and IUPAC, the crystal–chemical formula of ordered microporous compounds can be represented as follows [217]:

|*guest composition*|[*host composition*]_h_{*host structure*}_p_{*pore structure*} (Sym) − **IZA** (27)

The first two terms describe the chemical composition of the guest species (between boldface bars) and the host (between boldface square brackets), respectively. The next two terms (between boldface curly brackets) contain information about the structure of the host and the pores, respectively. The fifth term (between boldface round brackets) gives the symmetry of the material. If the host structure of the material belongs to a zeolite framework type, the sixth term (in boldface type, preceded by a dash) is the IZA code.

The simplified crystal–chemical formula of rhodesite (*Z* = 2) can be written as follows [1,128]:

|K(H_2_O)_4−*x*_|[Ca^[6]^_2_(H_2_O)_2_(Si^[4]^_8_O_18_OH)_∞∞_]*_h_*{3}*_p_*{2┴[8^6^][100] (8-ring)} (*Pmam*). (28)

This formula shows that the framework (“host”) consists of a heteropolyhedral structure formed by the layered silicate polyanion and calcium octahedra, characterized by two intersecting channel systems (with an eight-membered ring cross-section, forming a cavity with [8^6^] topology) perpendicular to the *a* axis of the crystal structure. The “guest” components are K^+^ cations and H_2_O molecules filling the wide channels.

Macdonaldite BaCa_4_[Si_8_O_18_(OH)]_2_·10H_2_O was first discovered at the sanbornite deposit in Big Creek, California (USA) and is named after Gordon Andrew MacDonald, an American volcanologist from the University of Honolulu [188]. Monteregianite-(Y) KNa_2_Y[Si_8_O_19_]·5H_2_O was named after its discovery locality in the alkaline massif of Mont Saint-Hilaire, Quebec (Canada). Both minerals are unstable in air, with dehydration beginning at room temperature and completing at 400 °C.

Structurally, macdonalsite and monteregianite-(Y) are similar to rhodesite but differ in terms of their composition and the structure of the *O*-layer [189]. While macdonaldite, like rhodesite, contains columns of edge-sharing Ca-centered octahedra, monteregianite-(Y) features an *O*-layer of edge-sharing [YO_6_] and [NaO_4_(H_2_O)_2_] octahedra, forming eight-membered rings consisting of two small, regular Y-centered octahedra and six large, distorted Na-centered octahedra. In macdonaldite, the large channels of the double layer with eight-membered rings are occupied by Ba-centered polyhedra, whereas in monteregianite-(Y), they contain 10-coordinated potassium cations and water molecules.

Melansonite, (Na,□)□_2_KZrSi_8_O_19_·5H_2_O, was discovered at the Poudrette (Demix) Quarry, Mont Saint-Hilaire, Québec, Canada [190]. Melansonite is paragenetically a late-stage mineral that is considered to have formed as a result of the interaction of late-stage alkaline fluids, enriched in SiO_2_ and ZrO_2_, that interacted with a marble xenolith under conditions of low *P* and *T* (below 200 °C).

Natromelansonite, Na_3_Zr[Si_7_AlO_19_]⋅4–5H_2_O, was also found at the Poudrette (Demix) quarry in a highly altered pegmatite together with a clay mineral, steacyite, polylithionite and rhodochrosite [191].

#### 6.3.2. The Delhayelite Structural Family

**Figure 29:**
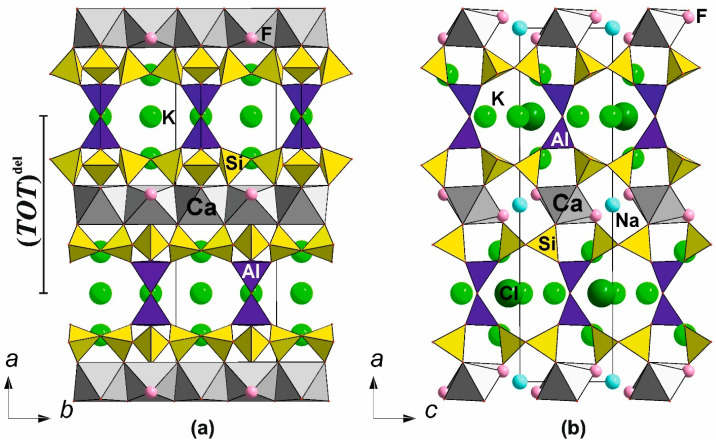
The general view of the crystal structure of delhayelite, projected onto (001) (**a**) and (010) (**b**).

The local symmetry of the (*TOT*)^del^ module is *P*2_1_/*m* [118]. In this module, the shlykovite-type layers of tetrahedra are shifted by a half-translation along the direction of the octahedral columns, analogous to the *TOT* module in the structures of shlykovite and cryptophyllite (Figure 25). In the currently known representatives of the delhayelite structural family (Figure 29), the *O*-layer consists of columns of edge-sharing [CaØ_6_]-octahedra (Ø = O^2−^, F^−^, OH^−^), with additional alkali cations possibly occupying interstitial sites. In the structure of delhayelite itself, the calcium-bearing octahedral columns alternate with columns of edge-sharing eight-coordinated [NaO_6_F_2_] polyhedra. However, due to natural ion exchange processes, sodium is leached out, and the structures of fivegite, hydrodelhayelite, and its Ba-analog are characterized by isolated [CaØ_6_]-octahedral columns within the *O*-layer.

In 1958, the description of “Mineral No. 3” from peralkaline pegmatites of the Khibiny Massif was published [218]. A year later, the name delhayelite was introduced for a new mineral found in alkaline lavas of Mount Shaheru in the Belgian Congo [219]. Subsequent comparisons of optical and X-ray diffraction data confirmed their similarity [220]. The crystal structure of delhayelite was first studied in 1969 [221] and later refined multiple times [222], leading to confusion regarding its ideal formula. Additionally, different samples of delhayelite exhibited significant compositional variations [203,220,223,224,225,226]. A recent re-examination of delhayelite from the Kirovsky Mine (Kukisvumchorr mount, Khibiny) provided new insights into its crystal chemistry and isomorphism, as well as its hydrated analog, hydrodelhayelite [203].

The chemical formula of “ideal” (“defect-free”) delhayelite can be written as K_4_Na_2_Ca_2_[AlSi_7_O_19_]F_2_Cl. However, delhayelite exhibits broad compositional variations and can also contain structural vacancies. Accounting for major isomorphic substitutions, its general written formula, when taking into account heterovalent substitutions, is (*Z* = 1): [**K**_8−*x*+*y*−*z*_(□,H_2_O)*_x_*_−*y*+*z*_]_∑8_(**Na**_4−*x*_Ca*_x_*)_∑4_**Ca**_4_{[(**Al**,Fe^3+^)_2+*y*_**Si**_2−*y*_]_∑4_**Si**_12_O_38−*z*_(OH)*_z_*}**F**_4_**Cl**_2_, where (*x*, *y*, *z*) < 1 and the elements in bold denote essential components [203,204]. Delhayelite and members of its transformation series (see below) are characterized by aluminum enrichment in the **u**-oriented tetrahedra of the shlykovite layer, forming [(Al,Si)_2_Ø_7_] groups (Ø = O^2−^, OH^−^) (Figure 28).

Fivegite was discovered in the same ultra-alkaline pegmatite body (at the Rasvumchorr mountain, Khibiny Massif) as shlykovite and cryptophyllite [204]. It is typically a secondary mineral replacing delhayelite (some samples contain relics of unaltered delhayelite within fivegite). Under late-stage hydrothermal activity, fivegite itself is replaced by aggregates of hydrodelhayelite and pectolite NaCa_2_[Si_3_O_8_(OH)]. The simplified chemical formula of fivegite (Z = 2), normalized to (Si + Al + Fe) = 8, is K_4_Ca_2_[AlSi_7_O_17_(O_2−*x*_(OH)*_x_*)]∙[(H_2_O)_2−*x*_(OH)*_x_*]Cl (*x* = 0–2), which shows that fivegite differs from delhayelite by lower Na and F content (the latter may be entirely absent) and a higher quantity of H_2_O.

Fivegite is a transformational mineral species [204,227], forming uniaxial pseudomorphs after delhayelite through structurally controlled alteration. From a crystal–chemical viewpoint, the transition from delhayelite to fivegite involves leaching of Na and F from the interlayer space, accompanied by hydration. The next-step alteration product is hydrodelhayelite. The transformation process can be represented by two reactions with water, as follows [204]:

K_4_Na_2_Ca_2_[AlSi_7_O_19_]F_2_Cl + 2H_2_O → → K_4_Ca_2_[AlSi_7_O_17_(O_2−*x*_(OH)*_x_*)][(H_2_O)_2−*x*_(OH)*_x_*]Cl + 2Na^+^ + 2F^−^ (29)

and

K_4_Ca_2_[AlSi_7_O_17_(O_2−*x*_(OH)*_x_*)]∙[(H_2_O)_2−*x*_(OH)*_x_*]Cl + (5 − *x*)H_2_O → → KCa_2_[AlSi_7_O_17_(OH)_2_]∙(6 − *x*)H_2_O + 3K^+^ + Cl^−^. (30)

Hydrodelhayelite, currently known only from the Khibiny Massif, is the alteration product of delhayelite and fivaite, involving the removal of F, Cl, Na, and most K, coupled with intense hydration. Its idealized formula is KCa_2_[AlSi_7_O_17_(OH)_2_]∙(6 − *x*)H_2_O [228]. Hydrodelhayelite is a chemical (but not structural) analog of rhodesite, differing primarily in the essential role of aluminum. In hydrodelhayelite, OH^−^ substitutes for O^2−^ at the vertices of [SiØ_4_] tetrahedra; the additional positive charge is compensated by partial potassium vacancies [203]:

K^+^ + O^2−^ → □^0^ (or H_2_O) + OH^−^, (31)

or excess of Al and/or Fe (more than 2 atoms per formula unit) in tetrahedral sites:

Si^4+^ + O^2−^ → (Al,Fe)^3+^ + OH^−^. (32)

Another compensation mechanism involves partial substitution of Na by Ca:

K^+^ + Na^+^ → □^0^ (or H_2_O) + Ca^2+^ (33)

and

Si^4+^ + Na^+^ → (Al,Fe)^3+^ + Ca^2+^. (34)

The substitution mechanisms are in agreement with compositional data, as delhayelite typically contains more than four Ca atoms per formula unit [203].

A new potential Ba-dominant analog of hydrodelhayelite (BDAH) was discovered at the Löhley quarry (Eifel paleovolcanic region, Germany) [145]. Its crystal–chemical formula (*Z* = 2) is (Ba_0.42_K_0.34_□_0.24_) (Ca_0.88_Fe_0.12_)_2_ (□_0.90_Mg_0.10_)_2_ [Si_6_(Al_0.5_Si_0.5_)_2_O_17_((OH)_0.71_O_0.29_)_2_] ∙6H_2_O. This mineral is hypothesized to form via Ba^2+^ ion exchange between hydrodelhayelite and lamprophyllite group minerals [186,229], which are abundant in Eifel rocks. This conclusion is supported by experimental data on ion exchange in natural phyllosilicates [230], where günterblassite (with a triple-layer silicate anion) was shown to readily adsorb up to 1.6 Ba atoms per formula unit from BaCl_2_ solutions. 

#### 6.3.3. Related Titanosilicates

**Figure 30:**
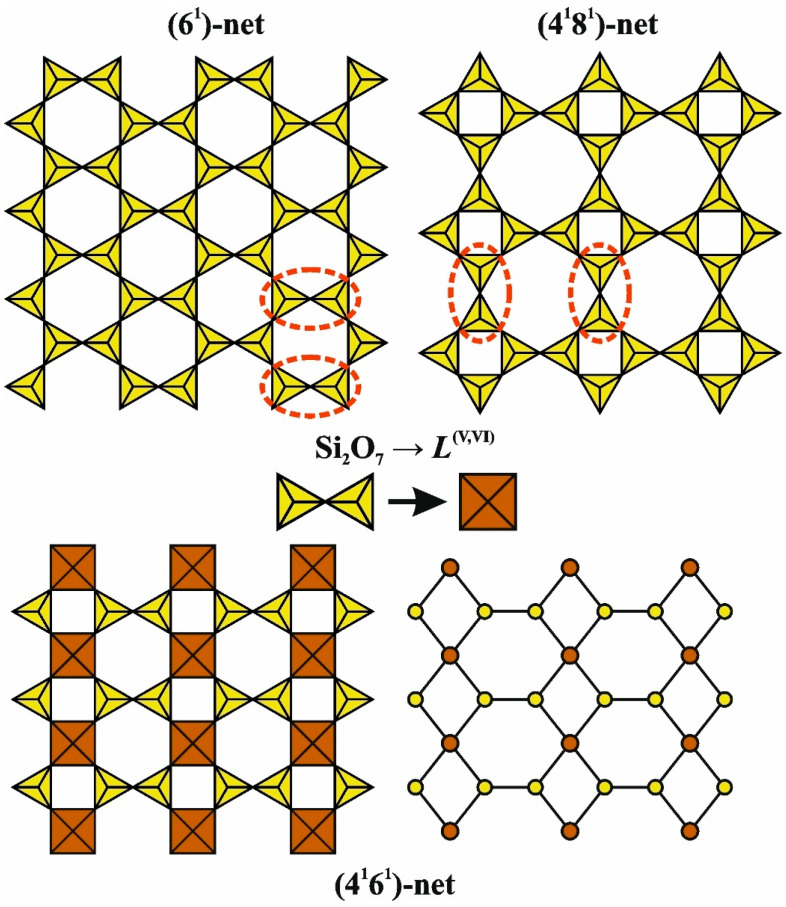
The possible transformation of tetrahedral (*T*) sheets of different topologies to mixed heteropolyhedral (*H*) sheets by functional group substitution.

Heterophyllosilicates [87,88,177] can formally be derived from pure phyllosilicates (in particular, micas [185]) by replacing tetrahedral (*T*) sheets with mixed (heteropolyhedral) *H*-sheets containing either five- or six-coordinated *L* cations (*L* = Ti, Nb, Fe, Zr) (Figure 30). The general formula of the *H*-layer is described by the following [88]:

(35) Lθ1+kSi2nO[2]3n+2O[1]2n,

where *k* = 0 or 1.

The crystal structures of heterophyllosilicates are based on the triple-layered *HOH* modules (similar to those in micas [185]), where the central *O*-sheet consists of edge-sharing *M*-centered octahedra. The stoichiometry of *HOH* modules in the crystal structures of heterophyllosilicates is described by the following general formula [88]:

(36) M3n+1Ψ2nLθ1+kSi2nO[2]3n+2O[1]2n2,

where Ψ is a [3]-connected ligand thar is coordinated only by *M*-cations. The negative charge of the *HOH* modules is compensated by the *A*-cations. Moreover, in the space between modules, additional anionic *Y*-groups [typically, *Y* = (PO_4_)^3−^, (SO_4_)^2−^, (VO_4_)^3−^, (CO_3_)^2−^], as well as water molecules can be present [179]. Taking into account the formula (44) the general formula for heterophyllosilicates can be written as follows [88]:

(37) AjYmH2OzM3n+1Ψ2nLθ1+kSi2nO[2]3n+2O[1]2n2.

Depending on the values of *n* and *k*, the *HOH* modules can be represented by different topological types (not only layered, but also framework-based) [231,232,233,234].

The heteropolyhedral *H*-layer with *n* = 1 and the ratio (*L*Θ_1+*k*_):(Si_2_O_7_) = 1:1 (in the crystal structures of the bafertisite structural family [179] or seidozerite supergroup [235]) can be obtained not only from the (6^1^)-net of the mica type, but also from the (4^1^8^1^)-net of the apophyllite type (Figure 30). This allows us to consider heterophyllosilicates as compounds related not only to micas, but also to members of the rhodesite structural family [179].

##### 2D-Titanosilicates: Jonesite

Jonesite Ba_2_(K,Na)[Ti_2_(Si_5_Al)O_18_(H_2_O)](H_2_O)*_n_* is a rare mineral discovered in 1977 from the Benitoite Gem Mine (San Benito County, California, USA) [236]. However, its crystal structure remained unstudied, primarily due to crystal quality and twinning. Advances in X-ray equipment and the introduction of two-dimensional CCD detectors allowed for the re-examination of jonesite, the interpretation and refinement of its crystal structure, as well as the characterization of the twinning features of its crystals [237]. The monoclinic unit cell parameters of jonesite are as follows: *a* = 10.618, *b* = 25.918, *c* = 8.695 Å, β = 127.633°; *V* = 1894.8 Å^3^; space group *P*2_1_/*m*; *Z* = 4.

The crystal structure of jonesite is unique and consists of double heteropolyhedral *H*-layers formed by tetrahedral diortho groups and octahedra in a ratio of (*L*Θ_2_):(Si_2_O_7_) = 1:1, which is well-known in minerals of the seidozerite supergroup [235]. These *H*-layers are connected via a disordered Al_2_O_7_ group, forming a two-layer heteropolyhedral polyanion with a topology similar to the double tetrahedral layers of minerals in the rhodesite subgroup. This similarity was noted by G. Ferraris, who considered jonesite as a mineral related to the rhodesite meropleiotypic series [179].

**Figure 31:**
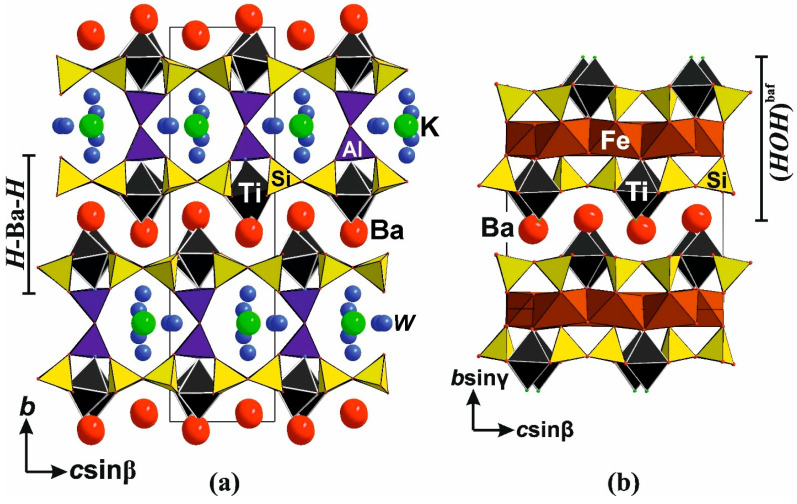
The general view of the crystal structure of jonesite (**a**) and its comparison with the crystal structure of bafertisite (**b**).

Despite having two-layer polyanions, jonesite lacks an octahedral *O*-layer, with large barium cations fulfilling this structural role [87,237]. Notably, a part of the structure (the *H*–Ba–*H* fragment) is similar to that in the structure of bafertisite, Ba_2_Fe_4_[Ti_2_O_2_F_2_(Si_2_O_7_)_2_](OH)_2_ (Figure 31) [238]. Considering that two- and three-layer representatives of the rhodesite meropleiotypic series tend to accumulate barium [120,206] and are found in association with barium-rich minerals of the lamprophyllite group [186,229], we hypothesize that jonesite may form at the contact between barytolamprophyllite Ba_2_Na(Na,Mn,Fe,Mg)_2_(Ti,Fe,Mg)[Ti_2_O_2_(Si_2_O_7_)_2_](OH,O,F)_2_ and, for example, hydrodelhayelite KCa_2_[AlSi_7_O_17_(OH)_2_]∙(6 − *x*)H_2_O [203].

##### 1D-Titanosilicates: Yuksporite

Yuksporite (Sr,Ba)_2_K_4_(Ca,Na)_14_(□,Mn,Fe){(Ti,Nb)_4_(O,OH)_4_[Si_6_O_17_]_2_[Si_2_O_7_]_3_}(H_2_O,OH)*_n_* (*n*~3) is a complex titanosilicate first described by Academician A.E. Fersman in 1923 [239] and named after its discovery site of Mount Yukspor (Khibiny, Kola Peninsula). As in the case of another titanosilicate, jonesite, the crystal structure of yuksporite remained unsolved for many years. The main obstacle was the fibrous habit of yuksporite crystals. The crystal structure of yuksporite was solved in 2004 using a set of experimental data obtained via synchrotron radiation (ESRF, Grenoble, France) [240]. The monoclinic unit cell parameters of yuksporite are as follows: *a* = 7.126, *b* = 24.913, *c* = 17.075 Å, β = 101.89°; *V* = 2966.4 Å^3^; space group: *P*2_1_/*m*; *Z* = 2.

**Figure 32:**
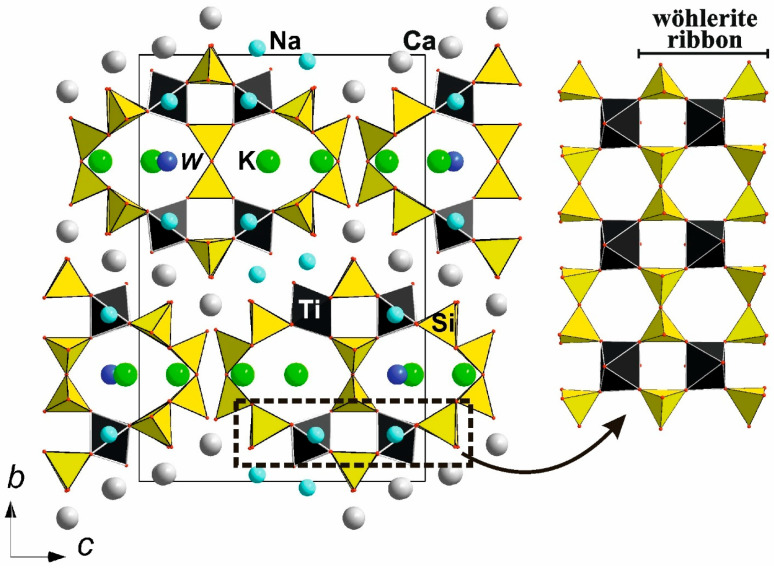
The general view of the crystal structure of yuksporite.

The crystal structure of yuksporite consists of one-dimensional extended titanosilicate polyanions with the composition {*L*^(VI)^_4_Ø_4_[Si_6_O_17_]_2_[Si_2_O_7_]_3_}_∞∞_ (*L*—Ti, Nb; Ø—O, OH), forming double tubes with an elliptical cross-section and external dimensions of 16 × 19 Å (Figure 32), which is a record for tubular fragments in mineral structures [240,241]. The tubes are formed by heteropolyhedral ribbons, which are a part of the bafertisite *H*-layer and can also be viewed as combinations of heteropolyhedral ribbons of the wöhlerite-type [242], formed by TiO_6_ octahedra and Si_2_O_7_ groups (similar to those in the structures of normandite Na_2_Ca_2_(Mn,Fe)_2_(Ti,Nb,Zr)_2_(Si_2_O_7_)_2_O_2_F_2_ [243] and janhaugite Na_3_Mn_3_Ti_2_(Si_2_O_7_)_2_(O,OH,F)_4_ [244]). A tetrahedral analog could be the unbranched three-membered double chain [Si_6_O_17_]_∞_ in the structure of xonotlite Ca_6_[Si_6_O_17_](OH)_2_ [245]. The heteropolyhedral ribbons are connected via [Si_2_O_7_]-diorthogroups at the edges and center, forming two parallel channels along the tube direction, characterized by an eight-membered cross-section and topology similar to that in the structures of minerals and synthetic compounds of the rhodesite-related compounds. The tubes are stacked along the c axis, forming a layer of parallel tubes. Adjacent tubular layers are separated by a “wall” formed by octahedra and seven-vertex polyhedra of sodium and calcium.

#### 6.3.4. Triple-Layer Silicates: The Günterblassite-Related Minerals

**Figure 33:**
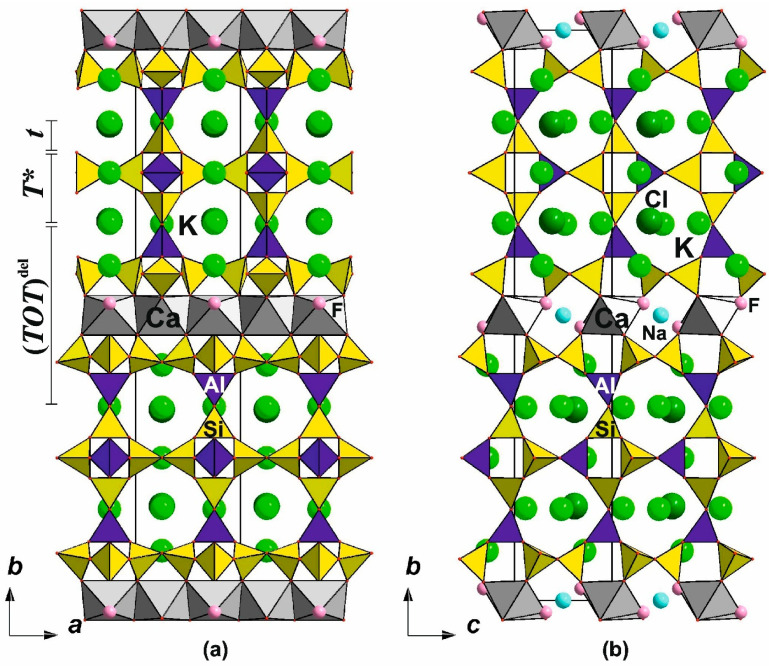
The general view of the crystal structure of umbrianite projected on (001) (**a**) and (100) (**b**).

The crystal structures of minerals belonging to the günterblassite subgroup are based on three-layer (*TOT*)^del^ modules, characterized by the local symmetry *P*2_1_/*m* and a half-translation shift in the outer tetrahedral *T*-layers relative to the central *O*-layer. Adjacent (*TOT*)^del^ modules are connected via “additional” T*-layers of the shlykovite type and *t*-tetrahedra, forming a three-layer polyanion with the general formula [*T*_13_Ø_29_] that was first described in the structure of günterblassite. The general sequence of modules in minerals of the günterblassite subgroup can be represented as (Figure 33)

…(*TOT*)^del^ + T* + *t* + (*TOT*)^del^ + T* + *t*…. (38)

Günterblassite (named after the renowned German amateur mineralogist Günter Blass) is the first silicate with a triple-layer tetrahedral polyanion [40,41]. Günterblassite samples were found in a basalt quarry on Mount Roter Kopf (Rhineland-Palatinate, Germany) as part of a late-stage association. It typically forms colorless, flattened (from thin-platy to tabular) crystals up to 0.2 × 1 × 1.5 mm in size, often showing regular (epitaxial or, more commonly, syntactic) intergrowths with nepheline and minerals of the lamprophyllite group [40].

The empirical formula of günterblassite, calculated for (Si,Al)_13_(O,OH)_29_ (based on structural data), is Na_0.15_K_1.24_Ba_0.30_Ca_0.72_Mg_0.16_Fe^2+^_0.48_[Si_9.91_Al_3.09_O_25.25_(OH)_3.75_]∙7.29H_2_O, which can be idealized as (K,Ca)_3−*x*_Fe[(Si,Al)_13_O_25_(OH,O)_4_]·7H_2_O.

**Figure 34:**
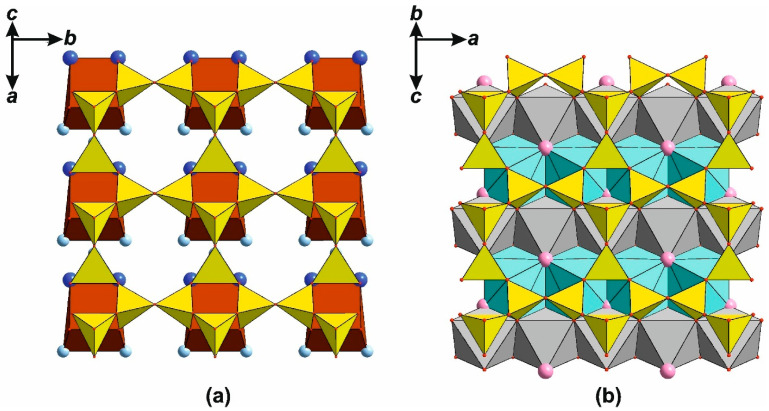
The *O*-layers in the crystal structure of günterblassite (**a**) and umbrianite (**b**).

In the structure of günterblassite [40], the *O*-layer (Figure 34) consists of highly disordered isolated seven-vertex polyhedra (coordinated by oxygen atoms of the silicon–oxygen polyanion, as well as OH groups and water molecules), primarily occupied by iron atoms (with admixtures of calcium, magnesium, and sodium). A comparison of günterblassite with other members of the group (hillesheimite and umbrianite) indicates strong cation disorder within the *O*-layer. It is likely that upon re-refinement of the structure using higher-quality samples, columns of edge-sharing [FeØ_6_] octahedra will be identified.

Hillesheimite was discovered in the active basalt quarry Graulay near the town of Hillesheim (Rhineland-Palatinate, Germany) as part of a late-stage association, where it is the latest formed and only hydrous mineral [121]. Hillesheimite forms flattened yellow to brown crystals up to 0.2 × 1 × 1.5 mm in size, with subparallel or fan-shaped aggregates of up to 2 mm across. The empirical formula of hillesheimite [121], calculated for (Si,Al)_13_(O,OH)_29_ (based on structural data), is K_0.96_Na_0.08_Ba_0.16_Ca_0.56_Mg_0.58_Fe^2+^_0.37_[Si_9.62_Al_3.32_O_23_(OH)_6_][(OH)_0.82_(H_2_O)_0.18_]∙8H_2_O, which can be idealized as (K,Ca,□)_2_(Mg,Fe,Ca,□)[(Si,Al)_13_O_23_(OH)_6_]OH·8H_2_O. As seen from the formula, the main differences between hillesheimite and günterblassite are the predominance of magnesium in the octahedral columns of the O-layer and a higher water content.

By comparison with günterblassite [40] and the transformation series delhayelite → fivegite → hydrodelhayelite [204], it is hypothesized that hillesheimite is also a transformational mineral species [121], formed through hydration and leaching of a significant portion of alkali cations, calcium, and possibly halogens from a hypothetical primary anhydrous cation-rich mineral like umbrianite [120], while preserving the original triple tetrahedral aluminosilicate layer.

Umbrianite was initially described as a “delhayelite-like mineral” due to the similarities in their chemical compositions [215]. The holotype umbrianite sample was found in melilitolites of the Pian di Celle volcano (Umbria region, Central Italy) [120] as a part of a late-magmatic association with leucite K[AlSi_2_O_6_], kalsilite K[AlSiO_4_], and fluorophlogopite KMg_3_[AlSi_3_O_10_](F,OH)_2_. Umbrianite forms rectangular, acicicular, and colorless transparent crystals of up to 0.02 × 0.03 × 0.2 mm in size. The empirical formula of umbrianite, calculated on the sum Si + Al + Fe = 13, is (K_6.45_Na_0.35_(Sr,Ba)_0.01_)_∑6.81_(Na_1.22_Ca_0.78_)_∑2.00_(Ca_1.85_Mg_0.13_Mn_0.01_Ti_0.01_)_∑2.00_[(Fe^3+^_0.34_Al_3.06_Si_9.60_)_∑13.00_O_29_]F_2.05_Cl_1.91_(OH)_0.04_. The idealized formula is K_7_Na_2_Ca_2_[Al_3_Si_10_O_29_]F_2_Cl_2_. Umbrianite differs from günterblassite and hillesheimite due to the predominance of calcium in the [*M*O_5_F] octahedra of the *O*-layer, the high content of extra-framework alkali cations (potassium and sodium) and large anions (primarily chlorine), and a reduced amount of water molecules.

**Figure 35:**
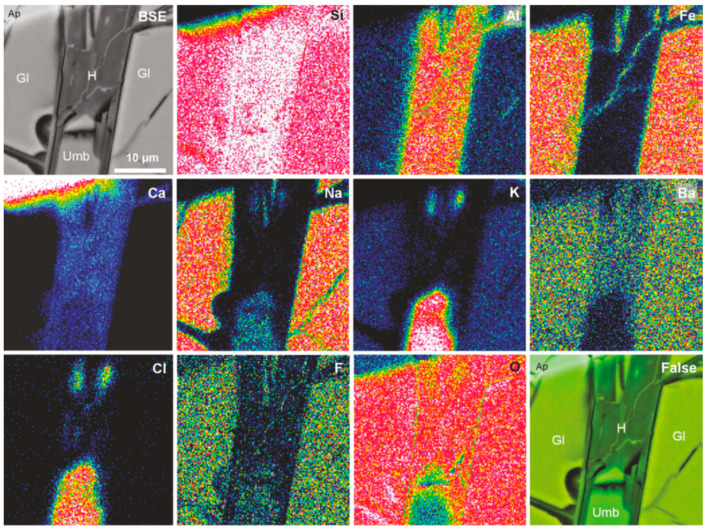
Chemical element distribution maps for umbrianite (Umb), replaced by a barium-rich hydrated phase (H); Gl—silicate glass. The figure is reproduced with permission from Sharygin et al. [120].

Umbrianite is highly unstable in post-magmatic aqueous solutions and transforms into a hydrated phase enriched in barium (BaO content varies and can reach 6.86 wt.% in samples from the Pian di Celle melilitolites) (Figure 35) [120], which retains the original crystal structure based on triple tetrahedral aluminosilicate layers. A high barium content (8.86 wt.% BaO, corresponding to 0.66 atoms per formula unit) has also been noted in a günterblassite-like minerals from the alkaline basalts of the Löhley deposit, found in association with high-barium minerals of the delhayelite series [206].

#### 6.3.5. Framework Silicates: The Edingtonite Subgroup

##### Natural and Synthetic Compounds with EDI-Type Frameworks

Edingtonite, Ba[Al_2_Si_3_O_10_]·4H_2_O, is a typical mineral of alkaline rocks, though it occurs in small quantities [246]. Depending on the ordering of Si and Al in the tetrahedral sites, tetragonal (space group *P*-42_1_*m*) and orthorhombic (space group *P*2_1_2_1_2_1_) varieties are distinguished, characterized by different unit cell parameters: *a* = 9.584 Å, *c* = 6.526 Å, *V* = 599.06 Å^3^ for the tetragonal modification [247,248,249] and *a* = 9.5341(6), *b* = 9.6446(6), *c* = 6.5108(7) Å, *V* = 598.68(8) Å^3^ for the orthorhombic modification [124,250,251,252].

The orthorhombic and tetragonal modifications of edingtonite are approximately equally common in nature and, in particular, in alkaline complexes [246]. They are not confined to specific rock types or mineral associations. For instance, tetragonal edingtonite has been reliably identified in carbonatites of the Khibiny Massif [227,249,253] and carbonatized jacupirangites of the Afrikanda massif [246] (both on Kola Peninsula), as well as in cavities in nepheline syenites of the Ice River massif (British Columbia) [254], while orthorhombic edingtonite has been found in nepheline-syenite pegmatites of the Khibiny Massif [253] and carbonatites of the Seblyavr and Kovdor Massifs [246], all located in Russia. Thus, unlike most other zeolites, which are rare or completely absent in carbonatites, edingtonite is a typical late-stage mineral, found in hydrothermally altered rocks developed on carbonatites and/or rocks closely associated with them [246].

The chemical composition of both edingtonite modifications from rocks of alkaline formations and objects of other genetic types [255] is identical and characterized by close-to-ideal stoichiometry, with the Si/Al ratio being very close to 2:3. Among the extra-framework cations, Ba predominates, and the content of impurities (Na, K, Ca, and Sr) does not exceed 0.5 wt.%. This feature of the chemical composition distinguishes edingtonite from other related minerals and, in particular, from representatives with **NAT**-type structures, in which a transition from the stoichiometrically ordered orthorhombic modification to a disordered tetragonal one leads to variability in the Si/Al ratio and the composition of extra-framework cations.

The thermal behavior of orthorhombic edingtonite was investigated in detail [256,257]. Edingtonite is a ferroelectric material of the Rochelle salt (RS) class and shows large property anomalies in the temperature range of −53 to −133 °C. It was shown that edingtonite blocks undergo symmetry lowering by the *P*2_1_2_1_2_1_ → *P*2_1_11 phase transition [257]. The crystal structure of edingtonite is characterized by the presence of two sublattices of water molecules and exhibits a complex low-temperature behavior. The most intense anomalous broad band at 220 cm^−1^ did not shift due to deuteration. According to the presented lattice-dynamical calculations, this vibration corresponds to a strong deformation of the cavity around the water molecules during the phase transition [257]. Edingtonite and thomsonite demonstrate anisotropic compression at room temperature and pressures to 6 GPa. The bulk modulus of edingtonite was found to be about 40% larger than that of thomsonite; *K*_0_^EDI^ = 73(3) GPa, *K*_0_^THO^ = 52(1) GPa [258].

Kalborsite, K_6_[Al_4_Si_6_O_20_][B(OH)_4_]Cl, is a rare mineral that was discovered on Mount Rasvumchorr in the Khibiny Massif [259], where it occurs as colorless and pale pink isometric grains (up to 2 mm) in pectolite rims around lovozerite segregations from rischorrite pegmatites. Kalborsite is endemic to the Khibiny alkaline massif, belonging to the potassium branch of ultra-agpaitic pegmatites, which host a wide range of other potassium-rich silicates and sulfides, such as wadeite, khibinskite, shcherbakovite, tinaksite, phenakite, delhayelite, djerfisherite, and prideite [246]. Its occurrence in the peripheral parts of lovozerite segregations indicate an initial association of kalborsite with the high-temperature analog of lovozerite, zirsinalite that forms substituting eudialyte upon its interaction with solutions highly saturated with alkalis [259]. Later, kalborsite was identified at the Kirovsky mine in pegmatitic veinlets [126]. Mineral relationships suggest that kalborsite forms simultaneously with lovozerite during the hydrothermal replacement of delhayelite by pectolite, inheriting K and Cl released during the decomposition of the former [246].

**Figure 36:**
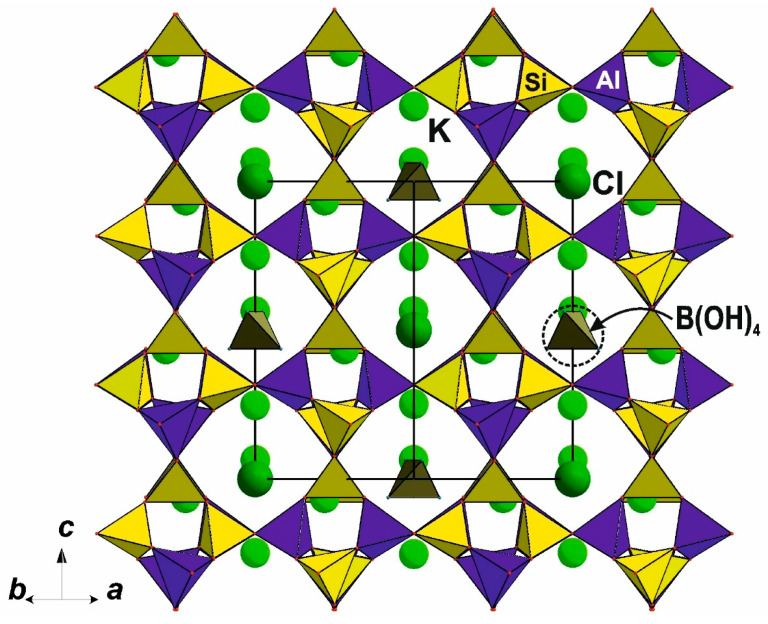
The general view of the crystal structure of kalborsite.

The crystal structure of kalborsite is based on the **EDI**-type framework; however, unlike edingtonite, kalborsite is characterized by a unique channel constituent configuration: Instead of water molecules, its structure contains two additional chloride anions, as well as a tetrahedral [B(OH)_4_]^−^ group (Figure 36) [125]. The incorporation of such anions and anionic groups into the crystal structure compensates for the excess positive charge arising from the replacement of two Ba^2+^ cations in the edingtonite structure with six K^+^ cations. These additional anions alternate in channels extending along the *c* axis which leads to a doubling of the *c* parameter to 13.1 Å compared to tetragonal edingtonite (*c* ~ 6.55 Å). IR spectroscopy data indicate that kalborsite contains a small amount of molecular water and local framework breaks, detected by the presence of a Si–OH band in the infrared spectrum [126].

Arzamastsevite, K_6_[Al_5_Si_5_O_20_][Si(OH)_4_]Cl, an analog of kalborsite with the substitution of the extra-framework [B(OH)_4_]^−^ group by the electroneutral [Si(OH)_4_] group, has been recently discovered in natrolitised sodalite syenite from the Eudialyte complex of the Lovozero alkaline massif, Kola Peninsula, Russia [127]. The substitution is controlled by the Si:Al ratio of 1:1 within the framework. The distribution of Si and Al between tetrahedral sites of the framework is disordered, which is realized in the more symmetrical space group *I*-42*m*, unlike the space group *P*-42_1_*c* for kalborsite [127].

Among synthetic analogs characterized by **EDI**-type frameworks, notable examples include synthetic edingtonite [260], tetragonal zeolites K-*F* and Na^ex^K-*F* [261], as well as RbNa-GaSi-EDI with the chemical formula Rb_7_Na[Ga_8_Si_12_O_40_]·3H_2_O [262,263] and ZnAsO_4_-EDI with the chemical formula (NH_4_)_3_[Zn_5_As_5_O_20_], also containing protonated 1,4-diaminobutane [264]. The Li-type EDI zeolite was successfully synthesized in the Li_2_O–Al_2_O_3_–SiO_2_–H_2_O system [265]. The aluminum cobalt phosphates ACP-EDI2 and ACP-EDI3 with the chemical formulas [NH_3_CH_2_CH(NH_3_)CH_3_]_2_[AlCo_4_P_5_O_20_] and [NH_3_CH_2_C(CH_3_)_2_(NH_3_)]_2_[AlCo_4_P_5_O_20_], respectively, as well as gallium cobalt phosphate GCP-EDI2 [NH_3_CH_2_CH(NH_3_)CH_3_]_2_[GaCo_4_P_5_O_20_] with the **EDI**-type frameworks, were also obtained using an amine-assisted synthesis approach with either 1,2-diaminopropane or 1,2-diamino-2-methylpropane [266]. It was shown that the success in synthesizing the new edingtonite-related materials is largely due to the realization of the importance of host–guest charge density matching in the cooperative inorganic–organic self-assembly processes [266]. Hydrothermally synthesized propane-1,3-diammonium bis (zinc phosphate), [H_3_N(CH_2_)_3_NH_3_][Zn_2_P_2_O_8_] is also an analog of edingtonite [267].

##### Natural and Synthetic Compounds with THO-Type Frameworks

Thomsonite-Ca, Ca_2_Na[Al_5_Si_5_O_20_]·6H_2_O, and thomsonite-Sr, (Sr,Ca)_2_Na[Al_5_Si_5_O_20_]·6-7H_2_O, form a continuous isomorphic series [246,268]. Thomsonite-Ca is widespread in various geological settings (including alkaline complexes), while thomsonite-Sr is an extremely rare mineral species found in hydrothermally altered rocks of only three alkaline massifs: Khibiny, Lovozero, and Afrikanda [246]. The main features of the chemical composition of minerals from the thomsonite series from alkaline massifs are as follows [246]:

1. The ratio of Si and Al is very stable, and the Si/Al ratio typically deviates only slightly from unity.
2. The Na: (Ca+Sr) ratio of ~0.5 also fluctuates little.
3. There is a strictly inverse relationship between the contents of Ca and Sr, and the Ca/Sr ratio varies widely: The strontium content ranges from trace to 19.4% SrO (Sr/Ca = 0.00–3.45), recorded in thomsonite-Sr from Mount Rasvumchorr in Khibiny, with no significant gaps observed in this isomorphic series.
4. Impurities of other cations (K, Ba, Mg, Fe) are insignificant.

Thomsonite-Sr is the most strontium-rich among all natural aluminosilicate zeolites [246,268], and minerals of the thomsonite series often act as the main concentrators of Sr in low-temperature alkaline hydrothermal rocks [246].

The symmetry of the **THO** framework type is orthorhombic, with space group *Pmma* with the unit cell parameters *a* ≈ 14.0, *b* ≈ 7.0, and *c* ≈ 6.5 Å [123]. However, the Si/Al-ordering and extra-framework content lead to the general symmetry of thomsonite describable with the space group *Pncn* and the unit cell parameters *a* ≈ 13.09, *b* ≈ 13.05, and *c* ≈ 13.23 Å [269,270,271]. Based on the significant number of reflections violating the two *n*-glides, different possible space groups (*Pmc*2_1_, *P*2*cm*, or *Pmcm*) have also been proposed [271]. The recent results on the crystal structure refinement and the analysis of the low-temperature structural evolution of natural “disordered” thomsonite with the chemical formula (Na_5.68_Ca_6.08_)[(Al,Si)_40_O_80_]·22.64H_2_O from Terzigno, Somma-Vesuvius volcanic complex, Naples Province, Italy, showed that the crystal of thomsonite is orthorhombic (space group *Pbmn*) with the unit cell parameters *a* = 13.0809(3), *b* = 13.0597(3), *c* = 6.6051(1) Å, *V* = 1128.37(14) Å^3^ [272]. The evolution of the unit cell volume with temperature (*T*) exhibits a continuous and linear trend between 98.0 and 295.5 K, without any evident thermo-elastic anomaly, with thermal expansion coefficient α*_V_* = *V*^−1^⋅∂*V*/∂*T* = 20(2)·10^−6^ K^−1^ [272]. Accurate structural analysis of the dehydration effect of natural thomsonite with the chemical formula Ca_3.34_Na_2.66_[Si_11_Al_9_O_40_]·12H_2_O (unit cell parameters: *a* = 13.0930(3), *b* = 13.0515(5), *c* = 6.5999(2) Å, *V* = 1127.81(7) Å^3^; space group *Pbnm*) by means of in situ single crystal X-ray diffraction indicated that the dehydration starts at 348 K [273]. Up to 498 K, thomsonite gradually releases four H_2_O molecules. From 498 to 573 K, an additional four H_2_O molecules are lost, and the space group changes from orthorhombic (*Pbmn*) to monoclinic (*P*2_1_/*n*). This partially hydrated phase is characterized by a unit cell volume contraction of 3% with respect to the initial room temperature phase and by a rearrangement of the extra-framework cations in the pores [273].

Among synthetic analogs, characterized by **THO**-type frameworks, there are synthetic thomsonite [260], as well related materials, in particular, the compound Rb-GaGe-THO with the chemical formula Rb_4_[Ga_4_Ge_4_O_16_]·3H_2_O, characterized by the acentric orthorhombic space group *Pn*2*n* [262].

Using two different doubly protonated derivatives of 1,3-diaminopropane and 1-amine(ethylene)-2-methylamine (*R*1 = [H_3_N(CH_2_)_3_NH_3_]^2+^, and *R*2 = [CH_3_(NH_2_)(CH_2_)_2_NH_3_]^2+^, respectively), the following monoclinic phosphate-organic complexes with **THO**-type frameworks have been obtained [141]: (*R*1)_2_[AlCo_4_P_5_O_20_] (ACP-THO1), (*R*1)_2_[GaCo_4_P_5_O_20_] (GCP-THO1, GCP-THO3), (*R*1)_2_[GaCo_4_P_5_O_20_](H_2_O) (GCP-THO4), (*R*2)_2_[AlCo_4_P_5_O_20_] (ACP-THO2), (*R*2)_2_[GaCo_4_P_5_O_20_] (GCP-THO2), as well as (*R*1)_2_[AlZn_4_P_5_O_20_] (AZP-THO3). Despite the differences in the stereochemical features of protonated amine and methylamine, the compounds ACP-THO1, ACP-THO2, GCP-THO1, and GCP-THO2 are characterized by the space group *C*2/*c* with the unit cell parameters *a* ≈ 14.1, *b* ≈ 13.2, *c* ≈ 13.9 Å, β ≈ 90.6º for *R*1 and *a* ≈ 13.8, *b* ≈ 13.2, *c* ≈ 13.8 Å, β ≈ 91.0º for *R*2. The compounds GCP-THO3 and AZP-THO3 are described by acentric space group *P*2_1_ with the unit cell parameters *a* ≈ 9.3, *b* ≈ 14.2, *c* ≈ 9.8 Å, β ≈ 95.3º, whereas a water-containing compound GCP-THO4 is characterized by centrosymmetrical space group *P*2_1_/*n* with the unit cell parameters *a* ≈ 12.9, *b* ≈ 14.3, *c* ≈ 14.2 Å, β ≈ 92.8º. The mixed hydrous phosphate of cobalt, zinc, and organic cation *R*1, (*R*1)_4_[Zn_(10−*x*)_Co*_x_*(PO_4_)_8_(HPO_4_)_2_](H_2_O)_3_ (*x* ≈ 3.45) (ZCP-THO; monoclinic, space group *Pn*), has also been obtained [274], while for protonated 1,4-diaminobutane there is a compound ZnAlAs-THO with the chemical formula [NH_3_(CH_2_)_4_NH_3_]_2_[Zn_4_AlAs_5_O_20_], characterized by the space group *P*2_1_/*n* [264]. In addition, the zinc phosphates, containing protonated tetraethylenepentamine (TEPA) and 1,2-diammonium propane cations with the chemical formulas [C_8_N_5_H_28_][Zn_5_P_5_O_20_]·H_2_O [275] and [CH_3_CH(NH_3_)CH_2_NH_3_][Zn_2_(PO_4_)_2_] [276], respectively, were also synthesized.

## 7. Polysomatic Series of Natrolite, Acp-1, and Montesommite

Based on the structural stereoisomerism of tetrahedral layers with the apophyllite-type topology, in addition to edingtonite polysomatic series, there are also the natrolite and ACP-1 polysomatic series, which currently consist of only two endmembers (mauntainite and natrolite as well as apophyllite and ACP-1), characterized by single-layer and framework tetrahedral polyanions, respectively. The polysomatic series of montesommaite has only one studied endmember (framework-type one), but the features of this series suggest the future discovery of a single-layer representative in addition to the double-layer representative, seidite-(Ce).

The structure of carletonite contains a double-layer tetrahedral polyanion formed by the condensation of nets with apophyllite-type topology. Thus, this mineral can be considered an intermediate member of another merotypic group.

### 7.1. Polysomatic Series of Mountainite

#### 7.1.1. Single-Layer Silicates: Mountainite

Mountainite, KNa_2_Ca_2_[Si_8_O_19_(OH)]·6H_2_O, is a hydrated silicate first discovered in a rhodesite sample as well-formed white crystals up to two millimeters in length [216]. It was named in honor of Edgar Donald Mountain, who first described rhodesite. Later, mountainite was found in the Yubileinaya pegmatite (Karnasurt Mountain, Lovozero, Kola Peninsula) [101], a notable geological formation where several new minerals with microporous heteropolyhedral structures were discovered, including the sodium titanosilicate zorite, the prototype of the microporous material ETS-4 with unique properties [277]. The crystal structure of mountainite was studied using material from the Yubileinaya pegmatite [101].

**Figure 37:**
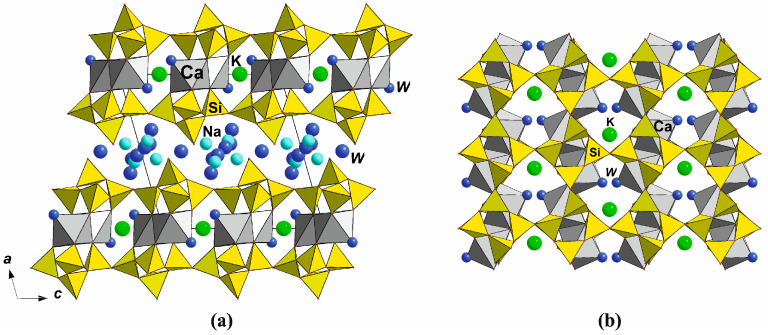
The general view of the crystal structure of mountainite projected on (010) (**a**) and the features of the *O*-layer, consists of zigzag columns of the CaO_5_(OH) octahedra (**b**).

In the tetrahedral layer of mountainite, with composition [Si_4_O_9_(O,OH)], one tetrahedron in each four-membered ring has an apical vertex oppositely oriented to the other three tetrahedra. However, unlike shlykovite and cryptophyllite [109], where all four-membered rings have the same orientation, neighboring rings in mountainite have opposite orientations along both the *b* axis (Figure 1e) and the *c* axis. This difference can be expressed as an orientation matrix: (**dddduddu**)(**dduddudd**) [**=**(**d^4^ud^2^u**)(**d^2^ud^2^ud^2^**)]. These minerals also differ in their *O*-layer structure: in mountainite, it consists of zigzag columns of the CaO_5_(OH) octahedra, and the silicate rings are alternately connected to one or another octahedron (Figure 37). Large cavities in the heteropolyhedral pseudo-framework contain K^+^ cations (coordinated with CN = 10) and water molecules.

Infrared spectroscopy confirms the presence of silanol groups (Si–OH) and a small amount of hydronium ions in mountainite, leading to the suggestion that a dynamic equilibrium exists: Si–OH^+δ^ + H_2_O ↔ Si–O^−δ’^ + H_3_O^+^. This phenomenon is common in acid salt hydrates [278,279,280].

In principle mountainite, shlykovite, and cryptophyllite could be grouped into a “mountainite structural family” (single-layer silicates with [4^1^8^1^] topology) because the differences between them are minor, as the mountainite layer is analogous to the silicate layers in shlykovite and cryptophyllite [101]. However, given the unique configuration of mountainite’s tetrahedral layer, which differs from shlykovite and resembles the layers in cavansite, ekanite, gillespite, cuprorivaite, and effenbergerite, we do not classify mountainite as part of the edingtonite polysomatic series.

#### 7.1.2. Framework Silicates: The Natrolite Subgroup

**Figure 38:**
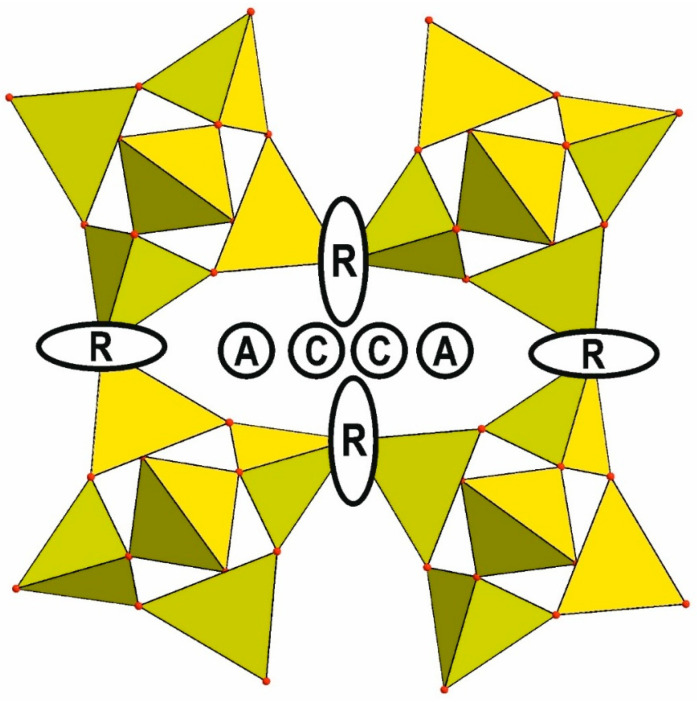
Schematic of the extra-framework subsystem based on the |**C**_2_**R**_2_**A**_2_|[*T*_5_O_10_] stoichiometry for the **NAT**-type structure. The positions along the *z*-axis are given in *c*/8 fractions.

The natrolite mineral series encompasses minerals with tetrahedral frameworks of the **NAT**-type topology: natrolite, Na_2_[Al_2_Si_3_O_10_]·2H_2_O, gonnardite, (Na,Ca)_6-8_[(Si,Al)_20_O_40_]·12H_2_O, paranatrolite, Na_2_K_0.25_[Al_2.25_Si_2.75_O_10_]·3H_2_O, scolecite, Ca[Al_2_Si_3_O_10_]·3H_2_O, and mesolite, Na_2_Ca_2_[Al_6_Si_9_O_30_]·8H_2_O. Compounds of the **NAT**-type topology can be represented by the crystal–chemical (positional) formula |**C**_2_**R**_2_**A**_2_|[*T*_5_O_10_] (Figure 38), where medium-sized cations Na^+^ and Ca^2+^ are located in **C**-positions, in proximity to the center of the channel; the large K^+^ cations and H_2_O molecules are situated in **R**-positions, in eight-membered rings where two [*T*_5_O_10_] chains join; “additional” **A**-positions may be occupied by H_2_O molecules [281]. The complete occupancy of all the positions was found in the structure of a high-pressure superhydrated natrolite phase [282].

Natrolite, Na_2_[Al_2_Si_3_O_10_]·2H_2_O, is the most common post-magmatic zeolite in intrusive alkaline complexes, being a rock-forming mineral in pegmatites and hydrothermalites, as well as in altered feldspathoid rocks. In the hydrothermalites of effusive alkaline rocks, the role of natrolite is significantly lower compared to other zeolites [246]. Due to the widespread distribution of this mineral, it is not possible to provide a detailed overview of all its known localities.

Natural natrolite is orthorhombic (space group *Fdd*2) with the unit cell parameters *a* = 18.29, *b* = 18.64, and *c* = 6.59 Å. The crystal structure of natrolite was first proposed by L. Pauling [283] and then was determined by W.H. Taylor [284]. Since that time, many other refinements have been reported [285]. Natrolite is characterized by a high (up to complete) degree of ordering of Si and Al over the tetrahedral sites. The difference between the *a* and *b* unit cell parameters is used to assess the degree of disorder in their distribution: this difference equals zero for a completely disordered tetragonal analog of natrolite and reaches 0.33 Å for the maximally ordered variety.

Paranatrolite, Na_2_K_0.25_[Al_2.25_Si_2.75_O_10_]·3H_2_O, is a rare zeolite, which was originally described in samples from miarolitic cavities and pegmatitic dykes in nepheline syenites (Mont St Hilaire, Québec, Canada) [286]. Paranatrolite is monoclinic with unit cell parameters *a* = 6.5952(12), *b* = 19.204(3), *c* = 9.955(2) Å, β = 107.737(12)°; space group *Cc*; *Z* = 4 [287]. For better comparison with natrolite, the unit cell may be transformed to a pseudo-orthorhombic unit cell with the parameters *a* = 18.971(4), *b* = 19.204(3), *c* = 6.5952(12) Å, β = 91.601(18)°; space group *F*1*d*1; *Z* = 8 [287]. In the crystal structure of paranatrolite, the dominant Na^+^ cations are situated near the sodium positions in the natrolite structure. Additional positions, occupied by K^+^, are located in the eight-membered rings. H_2_O molecules are located in four independent positions, two being occupied statistically [287].

To date, paranatrolite has been described in hydrothermalites from alkaline massifs [246]. Regarding its chemical composition, an essential K content is noteworthy, while Ca and Sr contents are less characteristic (their significant amounts have been noted only in samples from the Vishnevye Mountains). The special role of potassium as a factor stabilizing the paranatrolite structure was first noted by A.P. Khomyakov, based on a comparison of stable K-bearing paranatrolite from the Khibiny Massif with an unstable low-potassium analog from the Mont Saint-Hilaire Massif. This fact was confirmed by experimental data; when a Khibiny sample was treated with water, K leaching occurs much faster than Na leaching, after which the mineral loses stability and transforms into a low-hydration phase (with ~2.1 H_2_O molecules per formula unit) while retaining monoclinic symmetry [227]. Most probably, during potassium leaching, the charge balance is ensured by the entry of large hydronium cations into structural positions that are occupied by potassium in the original mineral. If this assumption is correct, then hydronium cations, along with potassium cations, can play the role of a factor stabilizing the monoclinic structure. Paranatrolite almost always forms later than natrolite, and the typical sequence of zeolite formation in the natrolite series for the Khibiny and Lovozero complexes is as follows [227]:

natrolite → paranatrolite → Ca-poor gonnardite (“tetranatrolite”).(39)

Gonnardite, (Na,Ca)_6-8_[(Si,Al)_20_O_40_]·12H_2_O, is less common than natrolite. It plays a significant role in late zeolite parageneses in many alkaline massifs [246]. As a rule, gonnardite crystallizes after other zeolites belonging to the natrolite series. It often overgrows natrolite, sometimes epitaxially. This mineral forms its most significant accumulations in agpaitic complexes. The value of the Na/Ca ratio, as well as the K and Sr contents, are indicators of the chemistry of the mineral-forming environment, primarily its alkalinity [246]. Gonnardite is also found in late hydrothermal formations associated with miaskites of the Vishnevye and Ilmen mountains (both in Southern Urals, Russia), nepheline syenites of Saint-Amable, Canada, pegmatites of Narsaarsuq, Greenland, alkaline basaltoids of Hegau in the Eifel region, Germany, carbonatites of the Tiruppattur complex in Tamil Nadu, India, and various other localities [246].

Gonnardite is tetragonal with unit cell parameters *a* = 13.21 and *c* = 6.62 Å; space group *I*-42*d* [285]. In line with the symmetry, there is only one type of channel, which is characterized by an “average” configuration intermediate between the Na-channel in natrolite and the Ca-channel in scolecite. This channel contains disordered (Ca,Na) atoms at two metal sites [285]. Gonnardite exhibits an enlarged framework charge that may be compensated by either the insertion of Ca^2+^ instead of Na^+^ or the presence of additional potassium cations in the mineral [227,271,288]. K-substituted gonnardite, extracted from a paranatrolite aggregate (Khibiny Mountains), with the chemical formula K_2.18_Na_0.04_Ca_0.02_[Al_2.26_Si_2.74_O_10_]·2.2H_2_O (unit cell parameters *a* = 13.65409(16), *c* = 6.56928(11) Å, *V* = 1224.74(2) Å^3^; space group *I*-42*d*) is characterized by the “excess” of the extra-framework cations [288]. In contrast to the original gonnardite, which undergoes a phase transition to the high-hydrate paranatrolite phase under conditions of elevated humidity, this K-substituted form is incapable of overhydration even under high pressure. Furthermore, in the range of 3.5–4 GPa, an anomaly in compressibility is observed, which can be interpreted as a phase transition to a monoclinic modification [289].

Scolecite, Ca[Al_2_Si_3_O_10_] 3H_2_O, occurs in vugs of basic volcanic rocks (basalt, andesite, and dolerite), rarely of metamorphic rocks (gneiss, and skarn), and amphibole gabbro. It has been described in association with other zeolites (natrolite, mesolite, thomsonite, heulandite, stilbite, laumontite, chabazite, levyne, and epistilbite), as well as prehnite, calcite, quartz, and pyrite [290]. Scolecite is quite rare for alkaline rocks; its findings were noted (without analytical data) in the Afrikanda intrusion of alkaline and ultrabasic rocks, Kola Peninsula [246] and Vishnevy Mountains, South Urals [291], but it has been reliably identified only in hydrothermalites related to peralkaline pegmatites of the Kovdor massif, Kola Peninsula [246].

**Figure 39:**
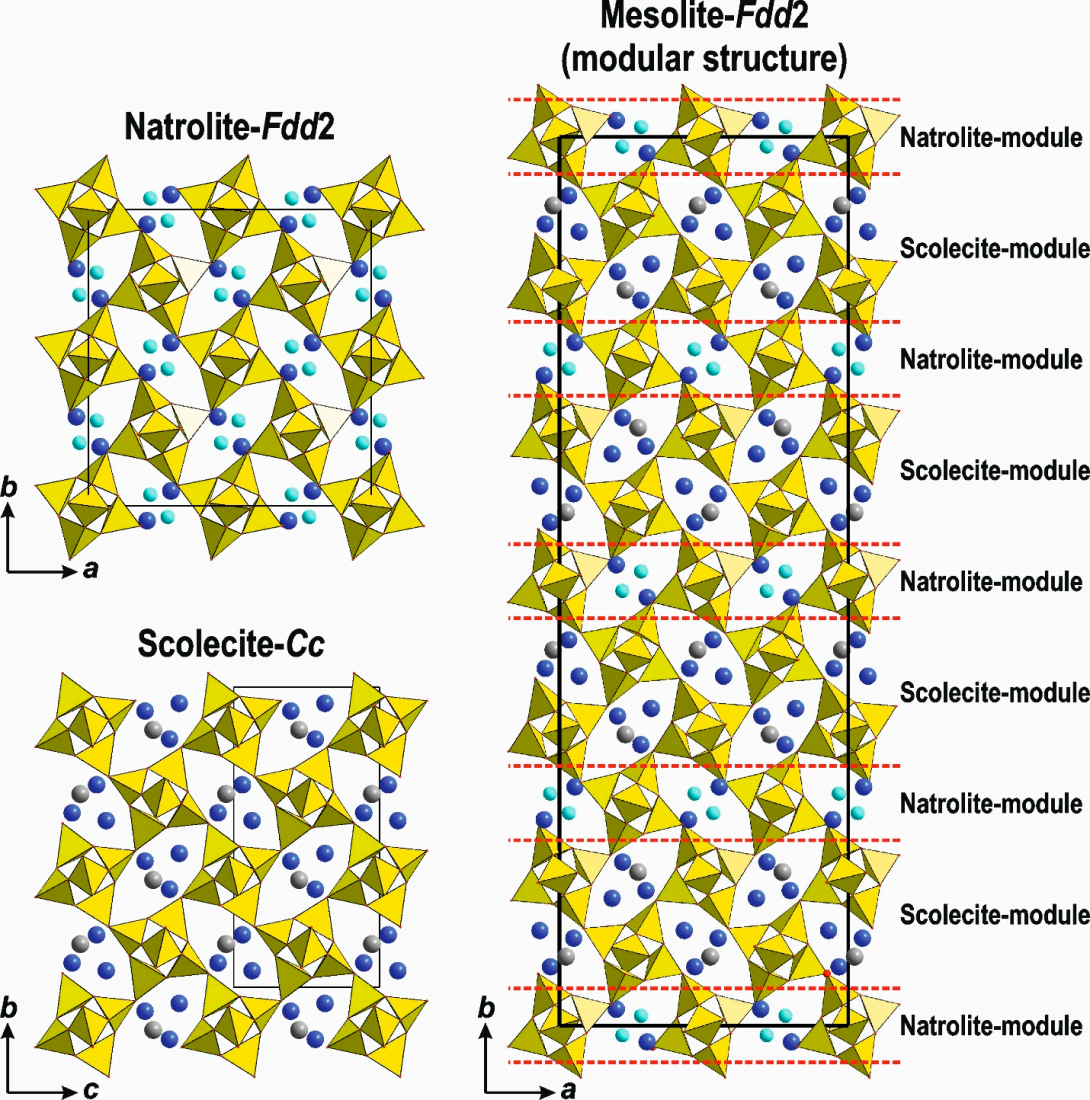
The crystal structures of natrolite, scolecite, and mesolite.

The crystal structure of scolecite is monoclinic *F*1*d*1 with unit cell dimensions (*a* = 18.50–18.55 Å, *b* = 18.90–18.97 Å, *c* = 6.52–6.53 Å, and β = 90.44–90.65°) correlating with the extra-framework cation contents [292,293,294]. The reduction in symmetry from orthorhombic (*Fdd*2) to monoclinic (*F*1*d*1) is related to the symmetry of the distribution of the channel cations. The 2_1_ axes in natrolite do not occur in scolecite because there is only one Ca per structural channel [285]. The other extra-framework cation site (the Na site in natrolite) is replaced by an H_2_O molecule, so each channel of scolecite contains one Ca site and three H_2_O molecules in accordance with the heterovalent substitution scheme Ca^2+^ + H_2_O → 2Na^+^ (Figure 39).

Mesolite, ideally Na_2_Ca_2_[Al_6_Si_9_O_30_]·8H_2_O, has been identified in hydrothermalites of alkaline massifs, including the Khibiny, Lovozero, and Vishnevye mountains, among others. Unfortunately, in most cases analytical data were not provided, with identification based only on optical constants [246]. Mesolite has been reliably identified in the Kovdor Massif, in vein-like feldspathoid syenites of the Phlogopite deposit, where it is associated with cancrinite. Furthermore, colorless, transparent mesolite crystals from Kovdor pegmatites have been studied; these are part of complex epitaxial overgrowths where mesolite crystallized after natrolite but before scolecite [295]. The samples also exhibited uniaxial overgrowths of mesolite on thomsonite (the [001] directions of crystals from both zeolites coincide), as well as independent druses of small crystals on fracture walls in feldspathoid rocks [246]. In the volcanic rocks of the Karadag massif (Crimean Mountains), mesolite occurs in amygdules and in calcite-zeolite veins, forming spherulites and later radiated aggregates [296]. Furthermore, through cationic exchange of Ca^2+^ for Na^+^, various pseudomorphs of natrolite after mesolite (accompanied by the formation of calcite) are formed according to the following scheme [296]:

Na_2_Ca_2_[Al_6_Si_9_O_30_]·*n*H_2_O + 2Na_2_(CO_3_)_solution_ → → Na_6_[Al_6_Si_9_O_30_]·*n*H_2_O + 2Ca(CO_3_) (40)

The crystal structure of mesolite (with almost ideal composition) has been accurate refined using a sample from Poona, India [297]. It was found that the mesolite structure has complete Si/Al order and the unit cell parameters have been determined as follows: *a* = 18.4049(8), *b* = 56.655(6), *c* = 6.5443(4) Å, *V*= 6823.94 Å^3^; space group *Fdd*2. Later, the distribution of Si and Al over tetrahedral sites has been analyzed by 1D (^29^Si, ^27^Al) and 2D (^27^Al) multiquantum (MQ) solid-state NMR, and it was shown that natural mesolite can be dealuminated under mild conditions using oxalic acid [298]. The accurate crystal structure reinvestigation of natural mesolite from Haldorsvik, Streymoy Region, Faroe Islands has been made using a multi-methodical approach, based on single-crystal neutron diffraction and in situ single-crystal synchrotron X-ray diffraction, using a diamond anvil cell [299]. The extra-framework population is represented by atomic sites occupied by Na, Ca and H_2_O, so that mesolite can be considered to be composed of 1/3 natrolite (ideally, |Na_2_(H_2_O)_2_| [Al_2_Si_3_O_10_]) and 2/3 scolecite (ideally, |Ca(H_2_O)_3_|[Al_2_Si_3_O_10_]) (Figure 39) [299].

Moreover, there are intermediate members of the natrolite–mesolite and mesolite–scolecite series (including in Kovdor) with a disordered distribution of Si and Al, which are reflected in the broadening and poor resolution of the IR spectrum bands related to framework vibrations [300].

The response of the mesolite crystal structure to dehydration was evaluated as a function of temperature and partial pressure of water [301,302]. The dehydration of mesolite occurs in two main steps, the first of which is generally considered to be the low-temperature extra-framework cation order–disorder phase transition. The resulting structure, metamesolite, is characterized by a random distribution of Na^+^ and Ca^2+^ cations within the channels. The second (high-temperature) step of dehydration may proceed via two different transition paths, depending on the partial pressure of water, *P*_H_2_O_ [301,302]. A structural transition from the Na/Ca-disordered mesolite variety (“metamesolite”) to a dehydrated phase (“x-metamesolite”) and amorphous *T*_5_O_10_ is observed at low-*P*_H_2_O_ environments, while a phase transition from Na/Ca-disordered metamesolite to pure amorphous *T*_5_O_10_ occurs at high-*P*_H_2_O_ conditions.

### 7.2. Polysomatic Series of ACP-1

#### 7.2.1. Single-Layer Silicates: Apophyllite Group

Apophyllite was named in 1806 by Rene Just Haüy from the Greek for “away from” (ἀπό, apo) and “leaf” (φύλλον, phyllos), in allusion to the way it exfoliates upon heating. The chemical composition of the members of the apophyllite group are described by the general formula *AB*_4_[Si_8_O_22_]*X*·8H_2_O where *A* = K, Na, NH_4_, Cs; *B* = Ca (in apophyllite-rootname members) or Sr (in hydroxymcglassonite-(K)); *X* = F, OH [303,304,305,306]. This group combines four mineral species: with *B* = Ca and *X* = F (fluorapophyllite-(K) [307], fluorapophyllite-(Na) [308], fluorapophyllite-(Cs) [309], and fluorapophyllite-(NH_4_) [310]), one species with *B* = Ca and *X* = OH (hydroxyapophyllite-(K) [305]) and one with *B* = Sr and *X* = OH (hydroxymcglassonite-(K) [311]). Members of the apophyllite group are hydrous sheet-structured silicates, which are frequently found associated with zeolites in rocks affected by low-temperature hydrothermal activity. Among them fluorapophyllite-(K), KCa_4_[Si_8_O_22_]F·8H_2_O, is the most common and widespread member of the group.

**Figure 40:**
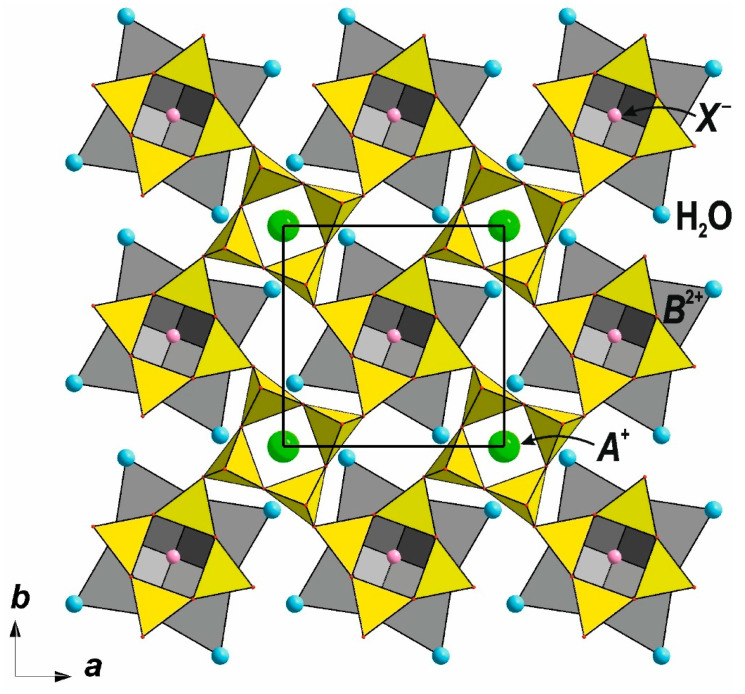
The general view of the crystal structures of apophyllite group minerals.

The crystal structure of fluorapophyllite-(K) was solved for the first time by W.H. Taylor and St. Naray-Szabo [312] and thereafter structures of apophyllite group minerals were refined many times using different samples and techniques [307,313,314,315]. The structures are based on silicate tetrahedral layers (characterized by the apophyllite-type stereoisomer), which are connected through the interstitial monovalent *A*^+^ and divalent *B*^2+^ cations (Figure 40). Most apophyllite group minerals are tetragonal (space group *P*4/*mnc*) with the unit cell parameters *a* = 8.96 and *c* = 15.8 Å [316]. The only exception is fluorapophyllite-(Na) [308], which is characterized by orthorhombic (pseudo-tetragonal) symmetry (sp. gr. *Pnnm*) with unit cell parameters *a* = 8.875(4), *b* = 8.881(6), and *c* = 15.79(l) Å. The symmetry reduction occurs due to the substitution of large K^+^ cation by a smaller Na^+^ cation at the *A* site. This observation also suggests that the apophyllite-type structure is capable of a hypothetical ordered orthorhombic structure with the Ca1 and Ca2 sites occupied by two different *B*^2+^ cations [311].

A synthetic compound AES-18 is isotypical with members of the apophyllite group and is characterized by the reported chemical formula KSr_4_[Si_8_O_12_(OH)_8_](OH)_9_, with the tetragonal unit cell parameters *a* = 9.12738(3), *c* = 16.28120(8) Å, sp. gr. *P*4_2_/*mnc* [113]. The crystal structure of AES-18 was elucidated by the charge-flipping method using powder X-ray diffraction data, and the obtained structure was refined by a combination with the Rietveld method and the maximum entropy method (MEM). The accurate analysis of the geometrical features crystal structure of AES-18 with the analysis of bond-valence sums allows us to conclude the real crystal structure of AES-18 should be similar to hydroxymcglassonite-(K), K(Sr,Ca)_4_Si_8_O_20_(OH)·8H_2_O [311], which is characterized by the comparable unit cell parameters *a* = 9.0792(2), *c* = 16.1551(9) Å, *V* = 1331.70(9) Å^3^.

During compression of the apophyllite group minerals in a water-containing medium, the effect of pressure-induced hydration is absent [317,318]. The pressure-induced hydration requires, in addition to a certain partial water pressure in a compressive medium, the presence of developed diffusion mobility of H_2_O molecules in the structure of a compound, or its development during the compression. Experimental results show that fluorapophyllite-(K) undergoes a crystalline–crystalline phase transition from a tetragonal structure (*P*4/*mnc*) to an orthorhombic structure (*Pnnm*) and then amorphization occurs under high-pressure conditions [319]. Raman peaks in the hydroxyl vibration region 2600–3800 cm^−1^ indicate a crystalline–crystalline phase transition occurring around 5.3 ± 0.9 GPa, and amorphization begins around 10 ± 0.9 GPa [319].

**Figure 41:**
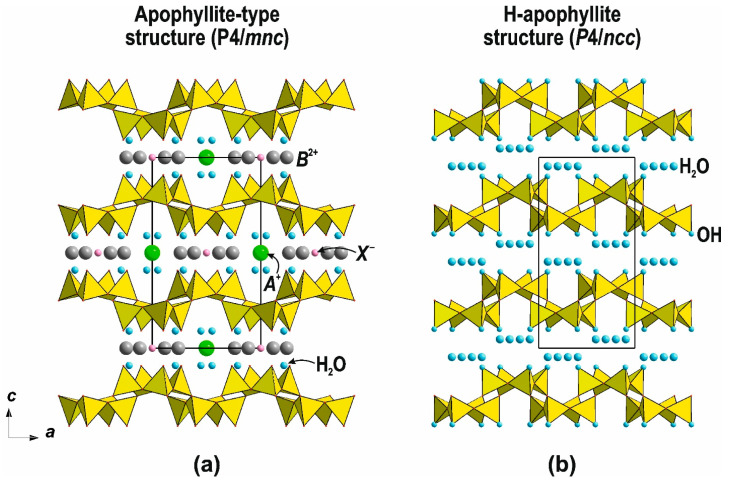
The relations between adjacent tetrahedral layers of the apophyllite-type stereoisomer: comparison of the apophyllite-type structure (**a**) with that of H-apophyllite (**b**).

Hydrated tetragonal H-apophyllite (HH-Apo) with the chemical formula H_16_Si_16_O_40_·8–10H_2_O has been recently synthesized at 0 °C by leaching an apophyllite single crystal in a large surplus of 1.2 molar hydrochloric acid [142]. It is characterized by the tetragonal symmetry (sp. gr. *P*4/*ncc*) with the unit cell parameters *a* = 8.4872(2), *c* = 16.8684(8) Å and *V* = 1215.08(7) Å^3^ [142]. In the crystal structure of HH-Apo, univalent *A*^+^ and divalent *B*^2+^ cations are absent, while the “outer” Ø-ligands of tetrahedra are fully protonated to compensate the negative charge. The tetrahedral layers are linked by strong hydrogen bonding. The main distinguishing structural feature of HH-Apo is the shift in the adjacent silicate layers by 1/2**a** + 1/2**b** in comparison with the apophyllite-type structure (Figure 41) [142].

The gillespite-group minerals and related synthetic compounds are described by the general formula *AB*[Si_4_O_10_], where *A* = Ca, Sr, Ba, and *B* = Mg, Fe, Cu, Cr [52,320,321,322,323]. Among them the following minerals are known: gillespite, BaFe^2+^[Si_4_O_10_][112], cuprorivaite, CaCu[Si_4_O_10_] [324], wesselsite, SrCu[Si_4_O_10_] [325], and effenberberite, BaCu[Si_4_O_10_] [326]. These compounds have had an importance in ancient art and technology being found as synthetic blue pigments in both pre-dynastic Egypt, 3600 BC, (Egyptian blue, CaCu[Si_4_O_10_]), but also from China, from the Western Zhou period, 1200 BC, (Han blue, BaCu[Si_4_O_10_]) [52,327,328,329,330,331]. Moreover, gillespite-type minerals are also characterized by the different physical properties (i.e., dielectric, luminescent) [332,333,334], due to unique structural features.

**Figure 42:**
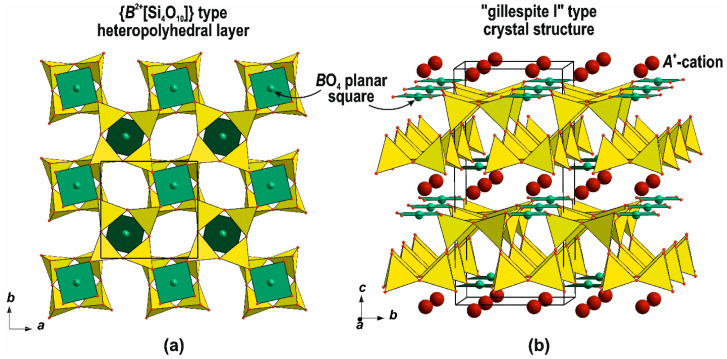
The apophyllite-type layers decorated by the *B*O_4_ square planar group, forming heteropolyhedral {*B*[Si_4_O_10_]}^2−^ layers (**a**) and the general view of the “gillespite I” type crystal structure (**b**).

The tetragonal so-called “gillespite I” type structure with the unit cell parameters *a* ≈ 7.5, *c* ≈ 16 Å (space group *P*4/*ncc*; *Z* = 4) is based on apophyllite-type layers (of the same type of stereoisomer), where apical oxygens of tetrahedra form the square planar coordination for *B*-cation (Figure 42a). The alkaline earth *A*-cations, located in a distorted cube environment, link the adjacent heteropolyhedral {*B*[Si_4_O_10_]}^2−^ layers (Figure 42b). The crystal structure exhibits a *c*/2 subcell, but the alternate left- and right-handed rotations of the *B*O_4_ square planar group and the corresponding anti-phase rotations of the connected four-membered silicate tetrahedral rings results in a doubling of the c axis length [323].

The “gillespite I” type structure is unstable under high pressures and undergoes a structural phase transition with the color change to the orthorhombic “gillespite II” type structure, characterized by the space group *P*2_1_2_1_2 [112]. Unlike the case with *B* = Fe^2+^ for gillespite, the presence of strong Jahn–Teller ions (Cr^2+^, Cu^2+^) in the square planar *B*-site result in a different high-pressure structure: tetragonal with the space group *P*42_1_2, or tetragonal with 3*a* × 3*a* × *c* supercell (space group is unknown) [335]. Using group theoretical arguments, it is possible to derive a hypothetical prototypic “gillespite 0” type structure with *c*/2 and space group *P*4/*nmm*. The “gillespite I” type structure can be derived from the “gillespite 0” aristotype by an instability at the Z-point of the Brillouin zone with the condensation of a soft-mode phonon with irreducible representation *Z*_3_^+^ [323].

#### 7.2.2. Framework Silicates: ACP-1

**Figure 43:**
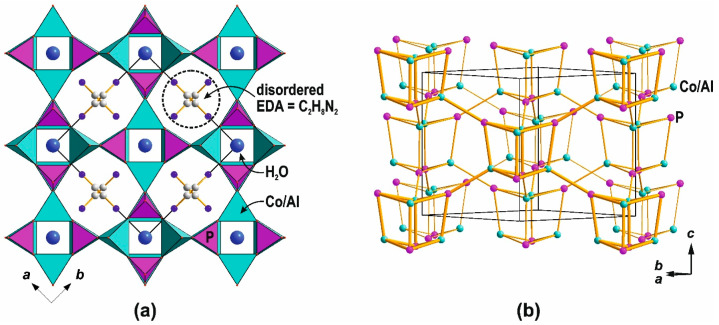
The general view of the crystal structure of compound ACP-1 (**a**) and the topological features of the tetrahedral framework of ACO type (**b**).

Organically templated aluminum cobalt phosphate ACP-1 with the chemical formula **|**(EDA)_16_(H_2_O)_8_**|[**Al_0.88_Co_7.12_P_8_O_32_] [where EDA is ethylenediamine, H_2_N(CH_2_)_2_NH_2_)] is the only representative characterized by the **ACO**-type framework [141]. The crystal structure of ACP-1 is tetragonal (space group *I*-42*m*) with the unit cell parameters *a* = 10.221 and *c* = 9.585 Å, while the idealized ACO-type framework is cubic (Table 3). Ethylenediamine molecules are located inside 8-ring channels and adopt two statistical orientations, while the water molecules center each D4R unit (Figure 43). Each water molecule is 2.31 Å away from four metal T atom sites of the D4R unit.

### 7.3. Polysomatic Series of Montesommaite

The montesommaite merotypic group includes only two structural representatives: the double-layer seidite-(Ce) and the framework montesommaite (and its synthetic analog). Nevertheless, by analogy with the rhodesite series, the discovery of other representatives can be expected in the future, since the rotation of tetrahedra in a layer of the seidite-type stereoisomer (Figure 1d) allows for the possibility of their direct layer-by-layer stacking Furthermore, the possibility of combining layered polyanions with isolated octahedra allows for the prediction of the future appearance of multilayer representatives (by analogy with the günterblassite subgroup).

#### 7.3.1. Double-Layer Silicates: Seidite-(Ce)

Seidite-(Ce), Na_4_(Ce,Sr)_2_{Ti(OH)_2_(Si_8_O_18_)}(O,OH,F)∙4.5H_2_O, is a microporous titanosilicate first described in the Yubileinaya pegmatite on Mount Karnasurt in the Lovozero Massif on the Kola Peninsula [336]. Seidite-(Ce) is a hydrothermal mineral formed during the final stages of ultra-agpaitic pegmatite crystallization from silicate-salt fluids enriched with alkalis, volatiles, and rare elements. Relics of these fluids are likely represented by glasses with the composition (Na,Ce,Th,Ti,Si,C)*_x_*O*_y_*∙*n*H_2_O [227].

**Figure 44:**
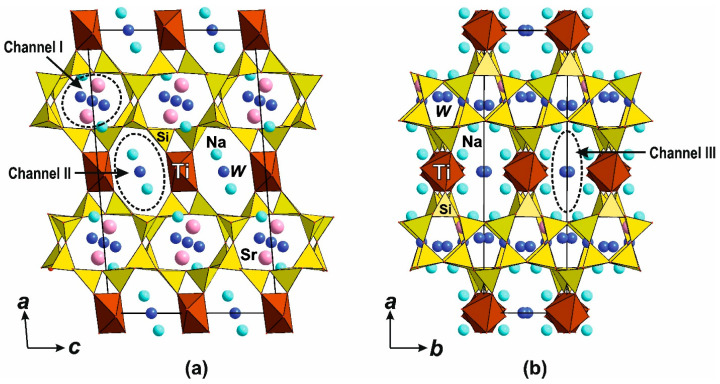
The general view of the microporous crystal structure of seidite-(Ce), projected on (010) (**a**) and (001) (**b**).

**Figure 45:**
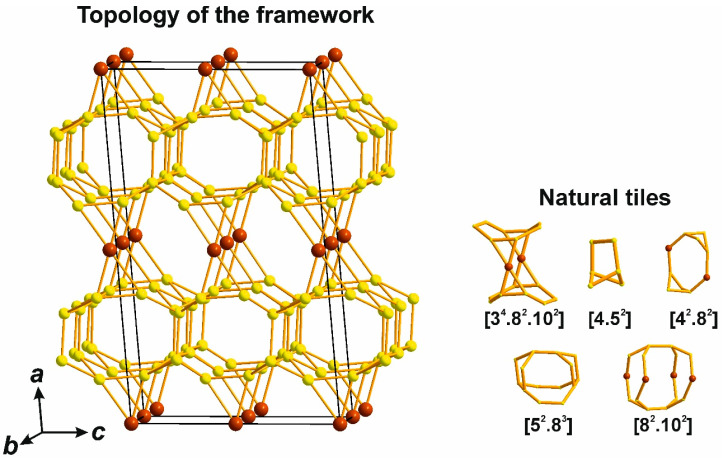
Topological features of the heteropolyhedral framework in the structure of seidite-(Ce).

Despite the typically fibrous nature of seidite-(Ce), a crystal suitable for determining its crystal structure was found [108]. The crystal structure is based on double-layer tetrahedral polyanions of composition [Si_8_O_18_]. These are built by combining layers with an apophyllite-type topology; however, they belong to a different structural isomer (Figure 1d) than those found in the merotypic series of shlykovite–edingtonite and mountinite–natrolite. These double-layer polyanions are connected via isolated TiØ_6_ octahedra (Ø = O, OH), forming a microporous heteropolyhedral framework {TiØ_2_(Si_8_O_18_)} (Figure 44). The topology of this framework (Figure 45) can be described by the tile sequence [4.5^2^]_2_[4^2^.8^2^][8^2^.10^2^][5^2^.8^3^]_2_[3^4^.8^2^.10^2^]. The foundation of the double-layer tetrahedral polyanion consists of [4.5^2^]- and [5^2^.8^3^]-tiles: specifically, **t-euo** and **t-kaj** tiles, respectively. The framework complexity parameters for the structure of seidite-(Ce) are *I*_G_ = 3.892 bits/atom and *I*_G,total_ = 225.763 bits/unit cell.

The heteropolyhedral framework in the structure of seidite-(Ce) features three systems of wide channels running along (001) (Channels **I** and **II**) and (001) (Channel **III**) (Figure 44). Channel I is located within the double-layer tetrahedral polyanion. It has an octagonal cross-section with effective cross-sectional dimensions of 3.0 × 5.3 Å and is occupied by sodium, strontium, and cerium cations, as well as water molecules. Channel II has an octagonal cross-section formed by six tetrahedra and two octahedra, with the effective dimensions 2.9 × 5.0 Å, and is occupied by sodium atoms and water molecules. Channel **III** has a highly elongated, elliptical 10-sided cross-section formed by eight tetrahedra and two octahedra, with the dimensions 0.9 × 6.2 Å and is occupied by water molecules. The wide Channels I and II are open channels that allow cation exchange. Ln contrast channel III contains water molecules; cation exchange cannot occur through it. The presence of open (unblocked) channels is confirmed by data on cation exchange in seidite-(Ce), which relates it to classical zeolites [108].

According to the IZA recommendations [2], the crystal–chemical formula of seidite-(Ce) can be written as follows:

(41) Na4Ce,Sr2H2O4.5Ti6OH2Si84O18∞∞3h1[5283/2][010](8−ring)2⊥821021008−ring10−ringp(C2/c)

in which it is shown that the “host” structure is formed by a heteropolyhedral framework. This framework consists of a layered silicate polyanion and titanium octahedra and is characterized by three types of channel systems. One channel extends along the b axis, while the other two intersect each other (forming an [8^2^10^2^]-cavity) and are perpendicular to the a parameter of the crystal structure. The “guest” species in the structure are Na, Sr, and Ce cations, along with H_2_O molecules, which occupy these wide channels.

#### 7.3.2. Framework Silicates: The Montesommaite Subgroup

**Figure 46:**
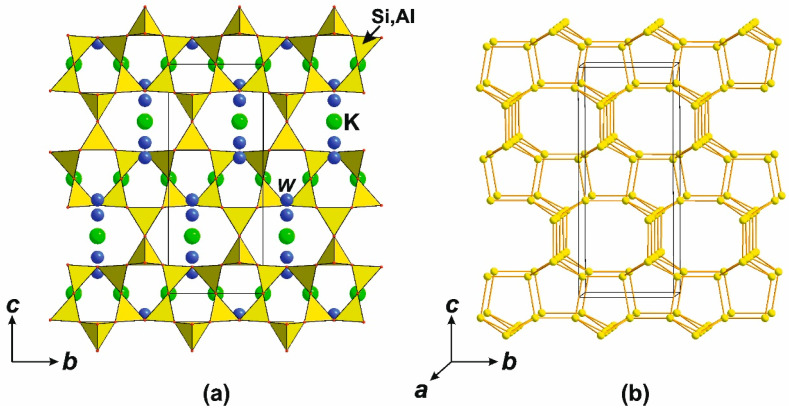
The general view of the crystal structure of montesommaite (**a**) and topology of the **MON**-type framework (**b**).

The endmember of the merotypic series formed by the condensation of tetrahedral layers of the seidite type (Figure 1d) is a tetrahedral framework of the **MON**-type [123], named after the mineral montesommaite in which it was first described (Figure 46a) [134]. Montesommaite, (K,Na)_9_(Al_9_Si_23_O_64_)∙10H_2_O, was found in rocks of the Monte Somma-Vesuvius volcanic complex, Valle d’Aosta, Campania, Italy in association with dolomite, calcite, chabazite, and natrolite. Besides montesommaite, this subgroup includes its synthetic germanium analog with the composition K_12_(Al_12_Ge_20_O_64_)∙8H_2_O [337].

The idealized **MON**-type tetrahedral framework (Figure 46b) is characterized by tetragonal symmetry (space group *I*4_1_/*amd*) and unit cell parameters *a* = 7.135 Å, *c* = 17.809 Å; *V* = 906.6 Å^3^. Its topology can be described by the following sequence of tiles: [4.5^2^][5^2^.8^3^] (corresponding to **t-euo**- and **t-kaj**-tiles, analogous to those in the structure of seidite-(Ce)). The framework density is 18.1 *T*/1000 Å^3^, and its complexity parameters are as follows: *I*_G_ = 1.918 bits/atom, *I*_G,total_ = 46.039 bits/unit cell.

The **MON**-type framework contains a system of identical, intersecting channels, {1[5^4^8^2^8^2/2^]<100>(8-ring)} [123]. These channels are topologically identical to Channel **I** in the structure of seidite-(Ce). They have an octagonal cross-section with the effective dimensions of 4.4 × 3.5 Å. The largest sphere that the framework can accommodate (*D*_P_) is 4.24 Å, and the largest sphere that can pass through the channels (*d_a_*) is 3.5 Å.

#### 7.3.3. Hypothetical Structures of Single-Layer and Multilayer Members of the Montesommaite Polysomatic Series

The existence of intermediate and endmembers in the polysomatic series allows for the modeling of hypothetical crystal structures for the endmember and intermediate members built by the condensation of tetrahedral layers of the seidite type.

**Figure 47:**
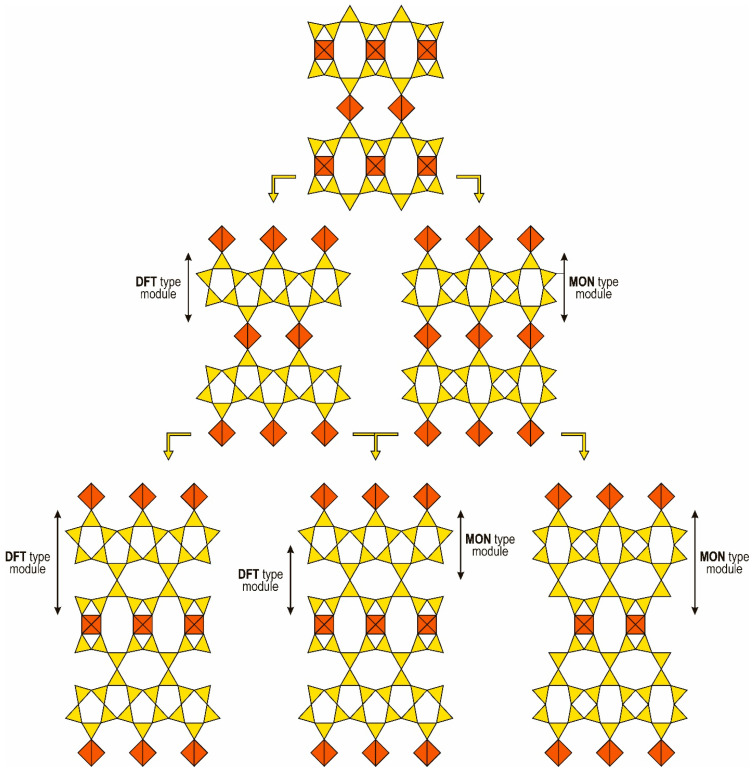
The hierarchical tree of the formation framework structures based on single-, double-, and three-layer polyanions with the layer of seidite-type stereoisomer.

By analogy with the structure of seidite-(Ce) [108], Figure 47 presents a hypothetical endmember structure of this polysomatic series, formed by single-layer tetrahedral polyanions [*T*_4_Ø_10_]. These are connected into a framework through isolated *M*φ_6_-octahedra (a similar connection via *M*φ_5_ square pyramids or *M*φ_4_ squares is also possible). The formula for such a heteropolyhedral framework would be {(*M*φ*_n_*)[*T*_4_O_10_]}, where its charge depends on the type of tetrahedral and *M*-cations, as well as the type of φ-ligand; *n* defines the coordination of the *M*-cation (0—square; 1—square pyramid; 2—octahedron). The rotation of tetrahedra in the seidite-type layer does not hinder layer-by-layer condensation, which could lead to the formation of structures with multilayer tetrahedral polyanions. For instance, a heteropolyhedral framework based on three-layer polyanions [*T*_12_Ø_26_] (=[*T*_12_O^br^_22_Ø^ap^_4_]), connected through additional octahedra, would have the formula {(*M*φ*_n_*)[*T*_12_O_26_]}. Both models are characterized by microporous structures and the presence of several systems of wide channels.

In reality, there are no crystal–chemical constraints forcing layered polyanions to combine into heteropolyhedral frameworks. The structures of shlykovite, cryptophyllite, and mountainite [101,109] demonstrate possible ways of preserving the overall layered motif.

## 8. Properties of Multilayer Silicates (Natural Analogs of 2D Zeolites)

Natural two-layer and three-layer silicates often exhibit high mobility of interlayer cations, which is manifested in the processes of leaching and ion exchange occurring under mild conditions. These minerals are characterized by high affinity for large uni- and divalent cations (NH_4_^+^, Ag^+^, Rb^+^, Cs^+^, Pb^2+^, Ba^2+^).

The ability of delhayelite to enter into ion exchange reactions with rubidium and ammonium salts was first noted by M. Chigarov [338]. In experiments on ion exchange of delhayelite with 1 N aqueous solutions of salts of various metals at temperatures of 80–90 °C and atmospheric pressure, it was shown [339] that this mineral is capable of accumulating up to 11.25 wt.% BaO, up to 11.89 wt.% Rb_2_O and up to 11.26 wt.% Cs_2_O within 3 h. At the same time, the contents of Na, K, F and Cl decrease. The IR spectra of the ion exchange products in all cases indicate parallel hydration while maintaining the double tetrahedral module.

An interesting case of the ion exchange process occurring in natural conditions (in miarolitic cavities in basalt) [206] is as follows: a Ba-dominant analog of hydrodelhayelite (i.e., Al-containing analog of mcdonaldite) with the crystal–chemical formula (Ba_0.42_K_0.34_□_0.24_)(Ca_0.88_Fe_0.12_)_2_(□_0.90_Mg_0.10_)_2_[Si_6_(Al_0.5_Si_0.5_)_2_O_17_(OH_0.71_O_0.29_)_2_]·6H_2_O was formed as a result of ion exchange of delhayelite with solutions containing barium due to the decomposition of an associated barium mineral of the lamprophyllite group [186]. As in the laboratory experiments, the ion exchange was accompanied by hydration. It should be noted that barium-containing (but not barium-dominant) members of the rhodesite and günterblassite groups have been repeatedly found by us in the Eifel basalts (Germany), and in all cases they were found in association with the decomposition products of barium minerals of the lamprophyllite group.

Delhayelite as an ion exchanger is inactive in reactions with lead salt solutions; however, our experiments have shown that günterblassite is capable of absorbing up to 10% PbO from dilute solutions of Pb(NO_3_)_2_ [339]. In addition, it has been shown [230] that at 30 °C günterblastite easily extracts barium (in quantities of up to 1.6 atoms *pfu*) from a 1 M BaCl_2_ solution.

Ion exchange properties of günterblassite in dilute solutions of various salts were studied [340]. In these experiments, 0.1 M aqueous solutions of AgNO_3_, RbCl_2_, CsCl_2_, CaCl_2_, CuSO_4_ and Pb(NO_3_)_2_ were used as electrolytes. The experiments were carried out at room temperature for one hour. Even under such mild conditions, günterblassite actively enters into ion exchange reactions with solutions of salts of large monovalent cations (Ag^+^, Rb^+^, Cs^+^), but exhibits significantly lower activity with respect to divalent cations (Pb^2+^, Ca^2^, Cu^2+^). The reactions proceed almost uniformly throughout the volume of the crystal. In this case, mainly H^+^, K^+^ and Na^+^ ions undergo substitution, while the atomic contents of the remaining components change insignificantly.

The degree of substitution of extra-framework cations and protons of silanol groups in gunterblassite is 55% for Ag, 52% for Rb, 45% for Cs, 7.2% for Pb and 2.7% for Cu, which corresponds to the following contents of the components in the reaction products: Ag_2_O 12.24, Rb_2_O 9.74, Cs_2_O 12.63, PbO 3.19, and CuO 0.43 wt.%, respectively. The calcium content in günterblassite in contact with the CaCl_2_ solution remained virtually unchanged.

Table 5 shows the composition of the product of natural ion exchange of günterblastite with Ba^2+^ ions that passed into the aqueous solution as a result of partial leaching from crystals of associated lileyite, Ba_2_(Na,Fe,Ca)_3_MgTi_2_(Si_2_O_7_)_2_O_2_F_2_, [341], a mineral of the lamprophyllite group [186]. The leaching and ion exchange processes occurred over a long period (tens of thousands of years have passed since the last eruption) and, therefore, the concentration of barium in the aqueous solution remained constantly at a very low level. Despite this, about half of the K+Na was replaced by barium, which indicates a high sorption capacity of günterblastite with respect to Ba^2+^.

In the search for new microporous materials for technological applications, compounds with the rhodesite structural type were obtained (Table 5). Synthetic analogs of rhodesite group minerals attract attention due to their important technological properties [179,189,197,342], since their channel system with an effective pore radius of about 3.2 Å is capable of passing an N_2_ nitrogen molecule [209] and exhibits catalytic and sorption properties [197,343]. As shown by IR spectroscopy data, the dynamic equilibrium Si–OH^+δ^ + H_2_O ↔ Si–O^−δ′^ + H_3_O^+^, noted above for mountainite, is also realized in two- and three-layer -silicates. Thus, the catalytic activity of these compounds may be associated not only with their microporous structure, but also with the presence of silanol groups, Si–OH.

A rhodesite-type compound with rare earth elements was characterized by the presence of photoluminescent properties [342,343]. Similar Na- and Ca-containing bilayer compounds are considered as potential components of cements and concretes [344,345,346].

It was shown experimentally that dehydrated synthetic analogs of monteregianite (AV-1), KNa_2_Y[Si_8_O_19_]·5H_2_O, and rhodesite (AV-2), KCa_2_[Si_8_O_18_(OH)]·6H_2_O, can be rehydrated [192,200]. The same compounds were used as catalysts for the isomerization of d-glucose to d-fructose [343,347]. In addition, synthetic rhodesite-type compounds are able to exchange Na and Ca for ammonia [348]. The dehydration of AV-1 is essentially complete at *ca*. 450–500 °C, and the total mass loss is 11.4%. AV-1 materials calcined at 650 °C and rehydrated in air, overnight at room temperature [192]. Three stages of dehydration occur, at 20–80 °C (loss of disordered zeolitic water molecules adsorbed within the channels), 80–220 °C [loss of water W(4), W(5), and W(6)], and 200–450 °C [loss of water W(1) and W(2)]. The dehydration of AV-2 lends at about 500 °C, and the total mass loss is 11.2%. Three stages of dehydration also occur, at 20–160 °C, 240–310 °C, and 310–430 °C. The AV-2 can be rehydrated reversibly in air, overnight at room temperature, after it is calcined at temperatures up to 500 °C [192].

AMH-3 type compounds like Na_2_Sr_2_[Si_8_O_19_]·4H_2_O [202] contain Sr, alkali metal cations and water molecules in their channels and, due to their resistance to acids and high temperatures, find application in polymer-silicate composites suitable for use as membranes and micro- and mesoporous materials [349,350,351]. Proton-exchanged AMH-3 was prepared by ion exchange of interlayer cations of the layered silicate AMH-3 using an aqueous solution of amino acid, in particular, dodecylamine [352]. Swollen AMH-3 was prepared by sequential intercalation of primary amine molecules following proton exchange [352]. Nafion-based composite membrane containing delaminated AMH-3 (D-AMH-3) layer was prepared by solution casting and hot pressing. It was shown that the D-AMH-3 layer is potentially suitable to be used as a permselective barrier for reducing vanadium crossover and improving cell performance [353]. Investigation of the transport of gas molecules (in particular helium, hydrogen, nitrogen, and oxygen) in a polydimethylsiloxane/(AMH-3 porous layered silicate) nanocomposite system has been studied as a function of its composition [354]. The transport properties were found to be strongly influenced by the molecular sieving properties of the AMH-3 layer.

The compounds **TR09**, Na_2_Sr_2_[Si_8_O_19_]·4H_2_O, and **TR10**, Na_4_Sr[Si_8_O_19_]·4H_2_O, were synthesized by the hydrothermal method at 503 K, and their crystal structures were solved by direct methods and refined to *R* = 2.1% and 3.3% [201]. The presence of large Sr^2+^ cation in the *O*-layer leads to a strong ellipsoidal deformation of the channels extending along [001]. Water molecules in the channels tend to be disordered along [100], i.e., across the channel. With close values of the parameters of monoclinic unit cells, the structures of these compounds are characterized by different space groups and different structures of the *O*-layer due to its different composition. **TR09** contains equal amounts of Na and Sr (two atoms per formula unit (*pfu*) with Si = 8) while **TR10** contains five such atoms *pfu* (Na_4_Sr). As a result, the *O*-layer in the **TR10** structure is flat and consists of edge-linked Na-octahedra, while the analogous layer in **TR09** contains polyhedra of different volumes—Na-centered octahedra (with Na–O distances of 2.3–2.5 Å) and 10-vertex Sr-centered polyhedra (with Sr–O distances of 2.5–3.1 Å). These polyhedra are linked by edges into six-membered rings, forming a highly corrugated layer.

The silicate material **AES-19**, K_8_Sr_8_[Si_32_O_76_]·16H_2_O, was obtained by hydrothermal synthesis at 150 °C [119]. This material exhibits adsorption and ion exchange properties similar to those of zeolites. The structure of **AES-19** was solved using powder diffraction data and refined by the Rietveld method. Sr atoms form octahedra, which are connected by edges in a zigzag chain running along [001]. K atoms and disordered water molecules are located in the channels of the structure. Sr atoms are easily removed from the structure by treatment with a weak acid, but in this case, the stacking of silicate layers and the expected formation of a three-dimensional zeolite framework are not observed. **AES-19** is chemically similar to **AMN-3** and differs from **AES-17** in the alkali cation (K instead of Na).

The compounds **TR03**, KNaCa_2_[Si_8_O_19_]·5H_2_O, and **TR04**, KNa_3_Sr[Si_8_O_19_]·4.3H_2_O, synthesized by the hydrothermal method at 500 K, are of mineralogical interest, since they are structurally related to delhayelite and hydrodelhayelite [209].

Lanthanide-based materials are also finding important applications in the field of fiber optics [343,355]. Microporous silicates containing stoichiometric amounts of *Ln*(III) with the general formula Na_2_K*Ln*[Si_8_O_19_]·*n*H_2_O (*Ln* = Eu, Tb, Er, Nd, Gd) are merged into the *Ln*-AV-9 family of materials [194,195,196,197] and are examples of microporous lanthanide silicates exhibiting both zeolite-like properties and photoluminescence (PL) in the visible region. The PL integrated intensity of Tb-AV-9 is 60% of the integrated intensity of the Gd_2_O_2_S:Tb^3+^ standard X-ray scintillator. The 1.54 μm emissions of hydrated Er-AV-9 are strongly enhanced with dehydration at 553 K [195].

## 9. Conclusions

Materials related to apophyllite are of significant industrial and technological interest because they are analogs of natural single-layer silicates. The varying orientation of tetrahedra relative to the tetrahedral layer plane, which determines the formation of structural isomers, creates prerequisites for their combination into multilayer tetrahedral polyanions (up to zeolite-type tetrahedral frameworks). Among compounds containing such multilayer polyanions, the most representative are those based on layers with the apophyllite-type topology. These layers combine either directly through layer-by-layer (topotactic) stacking or via additional tetrahedra linking adjacent layers. Furthermore, several compounds feature tetrahedral frameworks in which layers with the apophyllite-type topology can also be distinguished. The discovery of the first three-layer silicate, gunterblassite, made it possible to establish a fundamental crystal–chemical relationship between microporous silicate minerals and synthetic materials containing silicate layers with apophyllite-like topology.

The most representative family consists of silicates containing tetrahedral layers [Si_4_O_9_(OH)]^3−^ and [Si_4_O_10_]^4−^, found in the crystal structures of the mineral shlykovite and cryptophyllite. Such layers were first discovered as part of double layers in the structures of minerals of the rhodesite group and their synthetic analogs. The discovery of shlykovite and cryptophyllite confirmed the general topological and stereoisomeric features of these layers, as well as the possibility of their condensation to form multilayer silicate polyanions. The formation of double-layer polyanions can be represented as the polycondensation of shlykovite-type layers through free apical vertices (Ø^ap^) of tetrahedra oriented in opposite directions, leading to the formation of rhodesite-type double layers.

Further direct condensation of shlykovite-type layers is unlikely (or extremely rare), therefore polycondensation mechanisms were not considered for a long time. Possible transformation pathways, using the synthetic K,Sr-analog of rhodesite (AES-19) as a model, involved the extraction of alkaline earth elements from the original structure followed by topotactic condensation of rhodesite-type double layers. Based on the assumptions described above, a model of a hypothetical framework structure was proposed; however, to date, it has not been realized in either natural or synthetic compounds.

The determination of the crystal structure of the mineral gunterblassite revealed for the first time a three-layer polyanion retaining the apophyllite-type topology. This structure is formed by inserting an additional *T*O_4_ tetrahedron between the single [*T*_4_O^br^_6_Ø^ap^_4_] layers of the shlykovite/cryptophyllite type and the double [*T*_8_O^br^_13_Ø^ap^_6_] layers of the rhodesite type, leading to the formation of a triple layer [*T*^add^*T*_12_O^br^_23_Ø^ap^_6_] of the gunterblassite type. The günterblassite condensation mechanism (insertion of additional tetrahedra) suggests a pathway toward silicates with extended tetrahedral blocks (with more than three shlykovite-type layers) and, ultimately, zeolite-like frameworks (**EDI**, **THO** or **NAT** types).

The prepared review details the significant potential of apophyllite-type layers as versatile building blocks for creating both two-dimensional and three-dimensional zeolitic materials. The unique reactivity of these natural single-layer silicates, expressed in their ability to form hybrid organosilicate polymers with tunable hydrophobic–hydrophilic properties, underscores their industrial importance. Moreover, the discovery of multilayer analogs, such as gunterblassite, was key, as it revealed a fundamental crystal–chemical relationship unifying different families of microporous minerals and synthetic zeolites.

Based on the identified patterns, a classification scheme is presented, founded on the topological and symmetrical differences in apophyllite-type layers. This system provides a much-needed method for categorizing and classification known materials and serves as a predictive tool for the targeted synthesis of new zeolites. The modular nature of these layers, demonstrated in various synthesis strategies, opens up a vast design space for hierarchical porous materials with predefined diffusion pathways and active site distributions.

## Figures and Tables

**Table 2:** Topological types and main characteristics of tiles composing the **EDI-**, **THO-**, and **NAT**-type frameworks [123].

**Face Symbol**	**[4^3^]**	**[4^2^.8^2^]**	**[4^2^.8^2^]**
Figure	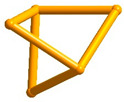	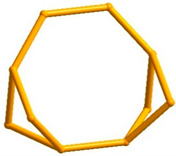	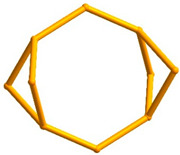
(V, E, F)	(5, 6, 3)	(10, 12, 4)	(10, 12, 4)
Symmetry	*mm*2	*mm*2	2/*m*
Tile label	**t-kzd**	**t-kdt**	**t-kds**
**Face symbol**	**[8^6^]**	**[8^6^]**	**[8.9^2^]**
Figure	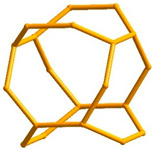	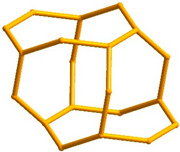	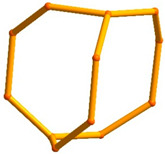
(V, E, F)	(20, 24, 6)	(20, 24, 6)	(10, 13, 3)
Symmetry	$\bar{4}2m$	.2/*m*.	.2.
Tile label	**t-krq**	**t-krr**	**t-nat**

Footnote. The values of V, E, and F are equal to the number of vertices, edges, and faces in the tile, respectively.

**Table 3:** The main topological features of frameworks composed by layers of the apophyllite-type topology [123].

Framework Type	Mineral/ Compound	Idealized Unit Cell Parameters (Å); *V* (Å^3^); sp. gr.*	*D_P_*/*d_a_*/*d_b_*/*d*_c_ (Å) **	FD ***	CBU	Tiles	Complexity Parameters		
							v (Atoms)	*I*_G_ (Bit/Atom)	*I*_G,total_ (Bit/Unit Cell)
Shlykovite-type stereoisomer									
**EDI**	Edingtonite (1*TT*-polytype)	*a* = 6.926 *c* = 6.410 *V* = 307.5 *P*-4*m*2	5.72/ 3.2/ 3.2/ 3.44	16.3	6*T*-nat	**t-kdt** **t-krq** **t-kzd**	15	2.174	32.603
**THO**	Thomsonite (2*O*-polytype)	*a* = 14.000 *b* = 7.000 *c* = 6.482 *V* = 635.2 *Pmma*	5.15/ 3.26/ 3.0/ 3.69	15.7	6*T*-nat	**t-kds** **t-kdt** **t-krr** **t-kzd**	30	2.840	85.207
Mountainite-type stereoisomer									
**NAT**	Natrolite	*a* = 13.850 *c* = 6.420 *V* = 1231.5 *I*4_1_/*amd*	4.52/ 2.99/ 2.99/ 4.38	16.2	6*T*-nat	**t-kds** **t-kzd** **t-nat**	30	2.174	65.207
Seidite-type stereoisomer									
**DFT**	DAF-2 (2*TT*-polytype)	*a* = 7.075 *c* = 9.023 *V* = 451.7 *P*4_2_/*mmc*	5.1/ 3.07/ 3.07/ 3.65	17.7	*nsc*-chain	**t-kaa** **t-lov** **t-ste**	24	1.918	46.039
**MON**	Montesomaite (4*TT*-polytype)	*a* = 7.135 *c* = 17.809 *V* = 906.6 *I*4_1_/*amd*	4.24/ 3.54/ 3.54/ 3.54	17.6	–	**t-euo** **t-kaj**	24	1.918	46.039
**NAB**	Nabesite	*a* = 7.184 *c* = 12.043 *V* = 621.4 $I\bar{4}m2$	4.31/ 3.57/ 3.57/ 3.57	16.1	5*T*-lov 6*T*-vsv	**t-nab-1** **t-nab-2**	15	2.174	32.603
**LOV**	Lovdarite	*a* = 7.163 *c* = 20.876 *V* = 1071.1 *P*4_2_/*mmc*	5.15/ 3.78/ 3.78/ 3.77	16.8	5*T*-lov 6*T*-vsv	**t-kaa** **t-lov** **t-nab-1** **t-nab-2** **t-ste**	54	2.755	148.764
**VSV**	VPI-7	*a* = 7.156 *c* = 41.842 *V* = 2142.7 *I*4_1_/*amd*	4.31/ 3.77/ 3.77/ 2.9	16.8	5*T*-lov 6*T*-vsv	**t-euo** **t-kaj** **t-nab-1** **t-nab-2**	54	2.755	148.764
**RSN**	RUB-17	*a* = 7.156 *b* = 41.910 *c* = 7.160 *V* = 2147.5 *Cmmm*	5.15/ 3.77/ 2.9/ 3.77	16.8	5*T*-lov 6*T*-vsv	**t-euo** **t-kaa** **t-kaj** **t-lov** **t-nab-1** **t-nab-2** **t-ste**	54	4.014	216.764
Apophyllite-type stereoisomer									
**ACO**	ACP-1	*a* = 9.905 *V* = 971.8 *Im*-3*m*	4.58/ 3.56/ 3.56/ 3.56	16.5	8*T*-*d4r*	**t-cub** **t-ste**	24	1.459	35.020
Paracelsian-type stereoisomer									
**GIS**	Gismondine	*a* = 9.801 *c* = 10.158 *I*4_1_/*amd*	4.97/ 3.32/ 3.32/ 3.32	16.4	20*T-*gis	**t-gsm**	24	1.585	38.039
Phillipsite-type stereoisomer									
**PHI**	Phillipsite	*a* = 9.890 *b* = 14.064 *c* = 14.046 *Cmcm*	5.4/ 3.69/ 3.11/ 3.31	16.4	24*T*-phi	**t-phi** **t-oto**	48	2.918	140.078
**SIV**	SIZ-7	*a* = 9.8768 *b* = 14.0754 *c* = 28.1314 *Cmcm*	5.38/ 3.73/ 3.31/ 3.37	16.4	20*T-*gis 24*T*-phi	**t-gsm** **t-phi** **t-oto**	96	3.835	368.156
Merlinoite-type stereoisomer									
**ATN**	MAPO-39	*a* = 13.071 *c* = 5.256 *I*4/*mmm*	5.91/ 1.94/ 1.94/ 4.11	17.8	24*T*-*atn*	**t-kaa** **t-ocn**	24	1.918	46.039
**MER**	Merlinoite	*a* = 14.012 *c* = 9.954 *I*4/*mmm*	6.65/ 3.12/ 3.12/ 4.2	16.4	16*T*-*d8r* (16*T pau*)	**t-opg** **t-pau** **t-ste**	48	2.252	108.078

* Parameters of the unit cell of the idealized silicon–oxygen framework [123], obtained by optimization of the real geometry. ** The maximum diameter of a sphere that can accommodate the framework (*D_P_*) and the maximum diameter of a sphere (*d_a_*_,*b*,*c*_), that can pass through channels extending along the unit cell parameters *a*, *b*, and *c*, respectively [129]. *** The framework density (FD) is defined as the number of tetrahedrally coordinated atoms (*T* atoms) per 1000 Å^3^.

**Table 4:** Minerals and synthetic compounds belonging to the edingtonite merotype series, containing shlykovite-type layers with the apophyllite-type topology.

Mineral/Compound	Chemical Formula	Sp. Gr. (*Z* = 2)	Unit Cell Parameters			References
			*a*, Å α, °	*b*, Å β, °	*c*, Å γ, °	
Single layer (*n* = 1)						
Shlykovite	K_2_Na_2_[Si_8_O_18_(OH)_2_]·6H_2_O	*P*2/*c*	6.4897	6.9969 94.597	26.714	[109]
Cryptophyllite	K_4_Ca_2_[Si_8_O_20_]·10H_2_O	*P*2_1_/*n*	6.4934	6.9919 94.680	32.087	[109]
Double layer (*n* = 2)						
*Rhodesite series*						
Rhodesite	KCa_2_[Si_8_O_18_(OH)]·6H_2_O	*Pmam*	23.416	6.555	7.050	[116]
Makdonaldite	BaCa_4_[Si_8_O_18_(OH)]_2_·10H_2_O	*Cmcm*	14.081	13.109	23.560	[188]
Monteregianite-(Y)	KNa_2_Y[Si_8_O_19_]·5H_2_O	*P*2_1_/*n*	9.512	23.956 93.85	9.617	[189]
Melansonite	(Na,□)□_2_KZr[Si_8_O_19_]·5H_2_O	*Pmma*	24.063	6.982	6.526	[190]
Natromelansonite	Na_3_Zr[Si_7_AlO_19_]⋅4–5H_2_O	*P*2_1_/*m*	6.516	24.061 90.453	6.976	[191]
AV-1	KNa_2_Y[Si_8_O_19_]·5H_2_O	*P*2_1_/*n*	9.595	23.956 93.850	9.583	[192]
AV-2	KCa_2_[Si_8_O_18_(OH)]·6H_2_O	-	23.797	7.031	6.598	[192,193]
AV-5	KNa_2_Ce[Si_8_O_19_]·5H_2_O	*P*2_1_/*n*	9.6906	24.055 93.683	9.5759	[193]
Eu-AV-9	KNa_2_Eu[Si_8_O_19_]·5H_2_O	*C*2/*m* *	23.973	14.040 90.352	6.5655	[194]
Tb-AV-9	KNa_2_Tb[Si_8_O_19_]·5H_2_O	*C*2/*m* *	23.945	14.019 90.288	6.5542	[194]
Er-AV-9	KNa_2_Er[Si_8_O_19_]·5H_2_O	*C*2/*m* *	23.951	14.013	6.550	[195]
Nd-AV-9	KNa_2_Nd[Si_8_O_19_]·5H_2_O	*Pmma*	23.902	6.998	6.547	[196,197]
Nd-AV-9	KNa_2_Nd[Si_8_O_19_]·2.8H_2_O	*Pmma*	23.923	6.995	6.547	[196]
Gd-AV-9	KNa_2_Gd[Si_8_O_19_]·5H_2_O	-	-	-	-	[197]
EMS-6	K_4_Na_8_Gd_4_[Si_32_O_76_]·17H_2_O	*P*2_1_/*n*	9.6595	23.9862 93.757	9.5275	[198]
CAS-1	K_2_Ca_2_[Si_8_O_19_]·4H_2_O	*C*2	24.158	7.0160 95.19	6.4816	[199]
AES-19	K_8_Sr_8_[Si_32_O_76_]·16H_2_O	*Pnna* *	13.9368	23.4754	6.7631	[119]
TR03	KNaCa_2_[Si_8_O_19_]·5H_2_O	*Pn*2_1_*m*	6.585	23.776	7.025	[200]
TR04	KNa_3_Sr[Si_8_O_19_]·4.3H_2_O	*P*2_1_/*m*	6.6599	23.7225 91.81	7.0225	[200]
TR09	Na_2_Sr_2_[Si_8_O_19_]·4H_2_O	*C*2/*c*	22.7681	6.9352 92.578	13.5789	[201]
TR10	Na_4_Sr[Si_8_O_19_]·4H_2_O	*P*2/*c*	22.5102	7.0292 92.538	13.3140	[201]
Phase **I**	KNa_2_Tm[Si_8_O_19_]·4H_2_O	*P*2/*m* **	6.5315	6.9935 90.383	11.9430	[118]
AMH-3	Na_2_Sr_2_[Si_8_O_19_]·4H_2_O	*C*2/*c*	22.783	6.9395 92.5935	13.581	[202]
*Delhayelite series*						
Delhayelite	K_4_Na_2_Ca_2_[AlSi_7_O_19_]F_2_Cl	*Pmmn*	24.579	7.0575	6.5811	[203]
Fivegite	K_4_Ca_2_[AlSi_7_O_17_(O_2−*x*_,OH*_x_*)] [(H_2_O)_2−*x*_OH*_x_*]Cl	*Pm*2_1_*n*	24.335	7.0375	6.5400	[204]
Hydrodelhayelite	KCa_2_[AlSi_7_O_17_(OH)_2_]·(6−*x*)H_2_O	*Pn*2_1_*m*	6.648	23.846	7.073	[205]
Ba-analog of delhayelite	Ba_0.5_Ca_0.5_[AlSi_7_O_17_(OH)_2_]·6H_2_O	*Pmmn*	23.953	7.052	6.606	[206]
Triple layer (*n* = 3)						
Günterblassite	(K,Ca)_3−*x*_Fe[(Si,Al)_13_O_25_(OH,O)_4_]·7H_2_O	*Pm*2_1_*n*	6.970	37.216	6.528	[40,121]
Umbrianite	K_7_Na_2_Ca_2_[Al_3_Si_10_O_29_]F_2_Cl_2_	*Pmmn*	7.062	38.420	6.574	[120]
Hillesheimite	(K,Ca,□)_2_(Mg,Fe,Ca,□) [(Si,Al)_13_O_23_(OH)_6_]OH·8H_2_O	*Pmmn*	6.979	37.182	6.530	[121]

* *Z* = 4; ** *Z* = 1.

**Table 5:** Chemical composition of günterblassite and its natural ion exchange product, “günterblassite-Ba”.

Component	Content (wt.%)	
	Günterblassite	“Günterblassite-Ba”
Na_2_O	0.40	0
K_2_O	5.18	2.85
MgO	0.58	0.81
CaO	3.58	2.98
BaO	4.07	8.84
FeO	3.06	3.45
Al_2_O_3_	13.98	13.56
SiO_2_	52.94	53.59
H_2_O	15.2	No data
Total	98.99	86.08

## Data Availability

No new data were created.

## Appendix A

Shlykovite is named in memory of Russian geologist Valery Georgievich Shlykov, a scientist and teacher at the Geological Faculty of Moscow State University. The name cryptophyllite comes from the Greek words κρυπτοζ (hidden) and φυλλον (leaf) due to the foliated character of the crystals. Both minerals were found in the Central apatite mine on Mount Rasvumchorr in the Khibiny alkaline massif (Kola Peninsula) [117,207]. Their crystals are transparent, colorless or faintly yellowish, visually indistinguishable from each other.

Shlykovite is more common than cryptophyllite and forms both intergrowths with it and monomineral segregations. The most common shlykovite individuals are leaflets or plates up to 0.02 × 0.2 × 0.5 mm (very rarely up to 0.03 × 0.3 × 1 mm), flattened along {001}. Cryptophyllite forms plates up to 0.02 × 0.1 × 0.2 mm, usually curved and/or split. It is found only in intergrowths with shlykovite, oriented such that individuals of both minerals grow together along {001}. Typically, shlykovite predominates in biminerallic aggregates, constituting 60–90% of their volume. Shlykovite and cryptophyllite aggregates sometimes contain microinclusions of unidentified K,Na-zeolites and potassium, sodium and titanium silicates.

The empirical formula of shlykovite, calculated on 13 O atoms, is (K_0.96_Na_0.09_)_∑1.05_Ca_1.00_Si_4.07_O_9.32_(OH)_0.68_∙3H_2_O; the OH/H_2_O ratio was calculated from charge balance. The charge-balanced empirical formula of cryptophyllite calculated on (Si,Al)_4_(O,OH)_10_ (based on the fact that protonation of apical **u**-inverted vertices of SiØ_4_ tetrahedra was established in structurally related shlykovite, and the OH/H_2_O ratio was calculated from charge balance) is (K_1.80_Na_0.17_)_∑1.97_Ca_0.99_Al_0.01_Si_3.99_O_9.94_(OH)_0.06_∙5.07H_2_O.

A distinctive feature of late-stage products in the pegmatite where shlykovite and cryptophyllite were found is their unusually low alumina content. At the same time, potassium activity in these solutions was probably significantly higher than calcium [117], since instead of the widespread apophyllite K_2_Ca_4_[Si_4_O_10_]_2_Ø∙8H_2_O (Ø = OH, F) with K:Ca ratio of 1:2, shlykovite (with K:Ca = 1:1) and cryptophyllite (with K:Ca = 1:2) formed. Cryptophyllite is probably less stable than shlykovite, as noted above, it has only been found in intergrowths with the latter.
